# Phylogenetic signal from rearrangements in 18 Anopheles species by joint scaffolding extant and ancestral genomes

**DOI:** 10.1186/s12864-018-4466-7

**Published:** 2018-05-09

**Authors:** Yoann Anselmetti, Wandrille Duchemin, Eric Tannier, Cedric Chauve, Sèverine Bérard

**Affiliations:** 1 ISEM, Université de Montpellier, CNRS, IRD, EPHE, Montpellier, France; 2 Univ Lyon, Université Lyon 1, CNRS, Laboratoire de Biométrie et Biologie Evolutive UMR5558, 43 Boulevard du 11 novembre 1918, Villeurbanne cedex, 69622 France; 3 INRIA Grenoble - Rhône-Alpes, 655 Avenue de l’Europe, Montbonnot-Saint-Martin, 38330 France; 4 Department of Mathematics, Simon Fraser University, 8888 University Drive, Burnaby, V5A1S6 BC Canada

**Keywords:** Scaffolding, Comparative genomics, Mosquito genomics

## Abstract

**Background:**

Genomes rearrangements carry valuable information for phylogenetic inference or the elucidation of molecular mechanisms of adaptation. However, the detection of genome rearrangements is often hampered by current deficiencies in data and methods: Genomes obtained from short sequence reads have generally very fragmented assemblies, and comparing multiple gene orders generally leads to computationally intractable algorithmic questions.

**Results:**

We present a computational method, ADseq, which, by combining ancestral gene order reconstruction, comparative scaffolding and *de novo* scaffolding methods, overcomes these two caveats. ADseq provides simultaneously improved assemblies and ancestral genomes, with statistical supports on all local features. Compared to previous comparative methods, it runs in polynomial time, it samples solutions in a probabilistic space, and it can handle a significantly larger gene complement from the considered extant genomes, with complex histories including gene duplications and losses. We use ADseq to provide improved assemblies and a genome history made of duplications, losses, gene translocations, rearrangements, of 18 complete *Anopheles* genomes, including several important malaria vectors. We also provide additional support for a differentiated mode of evolution of the sex chromosome and of the autosomes in these mosquito genomes.

**Conclusions:**

We demonstrate the method’s ability to improve extant assemblies accurately through a procedure simulating realistic assembly fragmentation. We study a debated issue regarding the phylogeny of the *Gambiae complex* group of *Anopheles* genomes in the light of the evolution of chromosomal rearrangements, suggesting that the phylogenetic signal they carry can differ from the phylogenetic signal carried by gene sequences, more prone to introgression.

**Electronic supplementary material:**

The online version of this article (10.1186/s12864-018-4466-7) contains supplementary material, which is available to authorized users.

## Background

The promises of using genes as evolutionary markers for phylogeny, introduced half a century ago by Zuckerkandl and Pauling [1], have been unfulfilled so far [2, 3]. At every scale of evolution, gene histories can differ from organisms tree-like phylogeny due to non-tree like evolutionary mechanisms such as incomplete lineage sorting, horizontal gene transfer, hybridization, symbiosis, among others. This happens for example in the *Gambiae complex*, composed of several African *Anopheles* mosquito species, whose phylogeny is important to shed light on the origin of malaria transmission to humans [4], but is difficult to trace because of apparent extensive gene introgression within this complex [5, 6]. Chromosomal rearrangements have been recognized, for an even longer time [7], as valuable phylogenetic markers, due to several reasons, including their lower occurrence rate compared to sequence evolution. They have proved to be of great interest for understanding mosquito evolution for example [8, 9], due to the fact introgression of whole chromosomes is much less frequent than introgression of genes [10]. Moreover, in terms of functional and ecological implications, chromosomal rearrangements have also been shown to be involved in important adaptation processes [11–13].

However chromosome evolution is still challenging to study, especially from short reads sequence data, and current methods have severe limitations that we outline now. First, some methods are limited to consider only chromosomal regions which are highly similar and whose differences are detected by genetic mapping, polytene chromosome banding, *in situ* hybridization or targeted sequencing [8]. Among methods which can handle whole genome sequence data [14–19], some consider only a small number of markers (often genes) with simple evolutionary histories (typically duplication-free histories and one-to-one orthologous gene families), and most of them require fully assembled extant genomes, aside of the recently published method DESCHRAMBLER [20]. As a consequence existing methods are hardly applicable to currently available genomic data, characterized by very short sequencing reads that can not resolve genomic repeats [21, 22], leading to highly fragmented genome assemblies, often in the form of hundreds or thousands of contigs or scaffolds, where evolutionary breakpoints can not be distinguished from assembly artifacts.

Various types of data can help to improve the contiguity of genome assemblies obtained from short reads. For example Third-Generation Sequencing (TGS) technologies generate long, albeit noisy, sequencing reads that can resolve ambiguities due to repeats [23]; alternatively, chromosome conformation capture technologies [24, 25] or genome maps [26–28] have also been used successfully for scaffolding large genomes. However in the absence of long range sequence data or genome maps, the most widely used approach to scaffold contigs is the comparative approach, using one or several related genomes to guide the scaffolding. The principle of comparative scaffolding is to align contigs of a fragmented genome assembly onto one, or a set of, assembled reference genome(s) and to deduce contig adjacencies from the contiguity of the corresponding alignments along the reference(s). Most comparative scaffolding methods rely on a single reference genome, assumed to be closely related enough that contiguity along the reference can confidently imply contiguity in the newly assembled genome [29–35]. Such methods have mostly been used to assemble pathogen genomes using closely related assembled references, but have also been shown to be applicable in wider contexts, such as scaffolding an antelope genome using a cow genome as reference [34]. There exists few methods that can handle several reference genomes at once, that can be distinguished between methods that do require that the phylogenetic relation between the considered genomes are provided [36–39] and methods that do not need such information [40]. Moreover, only two methods make use of sequencing data that might not appear in the contigs to be scaffolded but can provide a valuable scaffolding signal that complements the comparative signal [35, 37]. All such methods are also limited to handle contigs containing repeats and discard repeated contigs.

An important feature of most of these methods is that they assume a hypothesis of genome rearrangement parsimony or near-parsimony to transfer contiguity information from the reference(s) to the genome of interest, this hypothesis being either explicit [30, 31, 33, 38] or implicit [36, 39]. This points at the fact that the comparative approach is a kind of conundrum: to scaffold genomes, comparative methods rely, at least implicitly, on a framework to compare genomes and detect conserved synteny and chromosomal rearrangements, while whole genome evolution methods do not fare well when provided with fragmented genome assemblies.

We introduce a new computational method, ADSEQ, that addresses the issues raised above, regarding both genome evolution by rearrangements and comparative genome scaffolding; we apply it to simultaneously study the chromosome evolution and improve the scaffolding of 18 *Anopheles* genomes, 16 of them recently sequenced by Neafsey et al. [4], including several important malaria vector species. The method ADSEQ does not need a fully assembled reference genome, as is required by most comparative scaffolding methods, but takes as input a set, that can be arbitrarily large, of fragmented genome assemblies, together with a species phylogeny. It also takes advantage of sequencing data such as paired-end reads, for species for which it is available. From this input, ADSEQ computes ancestral genome segments, as well as extant scaffolding adjacencies. Additionally, ADSEQ allows the user to infer evolutionary scenarios in terms of gene duplication, gene loss, gene displacement and genome rearrangement along each branch of the species phylogeny. A Gibbs-Boltzmann probabilistic framework based on the cost of adjacencies gain and breaks in evolutionary scenarios provides a statistical support on all ancestral and extant inferred adjacencies, with sequencing data used to define a prior on extant gene adjacencies. To handle genes whose history involves duplication and loss, ADSEQ relies on the use of reconciled gene trees, in terms of gene duplication and losses, which allows to use a much larger gene set than existing comparative scaffolding methods relying on one-to-one orthologous genes or gene families with simple duplication/loss histories. We present, together with the ADSEQ method, a validation procedure for the extant genome scaffolding aspect of ADSEQ relying on a realistic framework to generate artificially fragmented genome assemblies.

Using ADSEQ, we provide an analysis of whole genome evolution in a large set of *Anopheles* species with an unprecedented precision, being able to quantify duplications, losses, gene displacements between chromosomes, and chromosomal rearrangements. We work at a much finer scale than cytogenetics methods [41–47], rely on a larger gene complement than traditional genome rearrangement methods based on rothologous genes, and we provide a refined evolutionary analysis compared to [4] due to the improvement of extant genome scaffolding. In particular we find that gene displacements between chromosomes are much more frequent for genes belonging to families with duplication/loss histories, that previous studies handling only one-to-one orthologous genes had mostly outlooked. We also use our method to compare two alternative *Anopheles* phylogenies. We find that *Anopheles* genomes are compartmentalized between autosomes and sex chromosome according to duplications and chromosomal rearrangements, just as they were found to be according to gene sequences. We provide an alternative hypothesis to the conclusions of Fontaine et al. [5] about introgression of the major part of the genome. Indeed our measures of rearrangements and duplications are in favour of the phylogeny supported by most genes.

## Methods

We first describe the main methodological contribution of our work, the ADSEQ tool, followed by its application to the specific *Anopheles* genomes data set we analyze in detail. We begin by introducing some simple but important terminology. A gene, extant or ancestral, is seen as a directed DNA segment with two *extremities*. Genes are parts of larger segments containing one or several genes, which are contig, super-contigs, scaffolds or even chromosomes for well assembled genomes. Two genes that are contiguous along such a segment define an *adjacency* between one extremity of each gene, that we call a *gene adjacency*. Thus *segments of genes*, either extant or ancestral, are encoded by linearly organized sets of gene adjacencies. For extant genomes, such segments, corresponding to contigs, scaffolds or chromosomes, are observed, while for ancestral genomes, segments are reconstructed and are to be considered as hypothetical as they are not directly supported by sequence data. Ancestral segments have previously been termed Contiguous Ancestral Regions (CARs) [19, 48, 49]. However, to stress the similarity between scaffold in extant genomes and CAR in ancestral genome, we use the generic terminology “segment” for both throughout this paper.

### Assembly recovery through detection of Coevolution with sequencing data (ADSEQ)

ADSEQ builds upon a family of methods aimed at reconstructing the evolutionary history of gene adjacencies introduced with the DECO algorithm [50] and extended along several lines in [51, 52]. It is implemented within the package DECOSTAR [53]. The aim of ADSEQ is to jointly scaffold extant genomes and reconstruct ancestral gene orders, through the joint analysis of phylogenomics and sequencing data.

ADSEQ takes as minimal input a species tree and a set of extant genomes data:gene adjacencies, homologous gene families and their associated reconciled gene trees (see for example [54] for background on reconciled gene trees). Reconciled gene trees implicitly yield the gene content of ancestral genomes. A key feature of ADSEQ is that the extant gene adjacencies can originate from genomes in various state of assembly, from fully assembled genomes —where gene adjacencies encode the gene order along the chromosomes—, to ambiguously assembled genomes represented as scaffolding graphs, through fragmented genomes assembled into contigs or scaffolds. Each extant gene adjacency is assigned a *prior* score in [0,1], expected to represent the confidence that the adjacency actually occurs in the genome of interest. This prior can be obtained from sequencing data or genome maps for example; so adjacencies between genes in fully assembled genomes will have a high prior score, while a potential gene adjacency in a poorly assembled genome and that is not supported by a strong signal in terms of sequencing data will likely be assigned a low prior score.

**Fig. 1:**
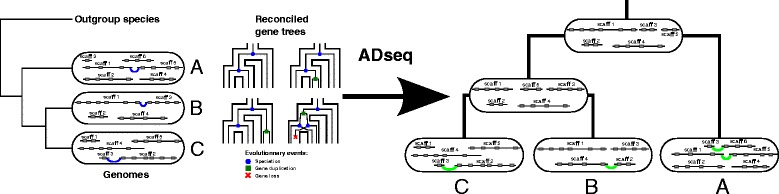
Input and output of ADSEQ. (Left) Input data: (1) a species tree with extant genomes (A, B and C) containing observed adjacencies (black link) and scaffolding gene adjacencies with a *prior* score (blue link); each grey box represents a gene. (2) reconciled gene trees representing evolutionary histories of gene families annotated by evolutionary events. (Right) Typical output: gene order across ancestral and extant genomes including new extant gene adjacencies with a *posterior* score (green link) between genes located at fragments extremities in the initial genome assemblies

ADSEQ processes independently all pairs of gene families for which at least one extant adjacency is observed between genes from these families. A *solution* of ADSEQ on such an instance is a set of extant and ancestral gene adjacencies between extant and ancestral genes of the two considered families, that are consistent with the given reconciled gene trees. Taking the obtained solutions over all pairs of gene families defining ADSEQ instances, ancestral adjacencies link ancestral genes extremities into ancestral genome segments, while extant adjacencies improve the scaffolding of the provided extant genomes and reduce their fragmentation. This, together with the reconciled gene trees, in turn provides an important input material for studying whole-genome evolution through mechanisms such as gene duplication, loss and transfer, introgression, or genome rearrangement. Figure 1 provides a high-level overview of the ADSEQ algorithm.

An important feature of ADSEQ is that, for each considered instance, it does not compute a single solution, but samples solutions from the, often large, associated search space. In order to sample solutions, ADSEQ relies on a *score*, function of the number of evolutionary events and the prior score of adjacencies. Any solution indeed yields a number of gains and breakages of gene adjacencies, that model genome rearrangement events consistent with the provided reconciled gene trees (see a description of the propagation rules that allow to infer gains/breakages from reconciled gene trees in the DECO algorithm [50]). The *score* of a solution *S* is defined as *n*(*S*)=*g*(*S*)+*b*(*S*)+*c*(*S*) where *g*(*S*) is the number of gene adjacency gains scaled according to a user-defined unit cost of a gain; *b*(*S*) is the number of gene adjacency breakages again scaled according to a user-defined unit cost; *c*(*S*) is the cost of including or discarding extant adjacencies, based on their prior score: for an adjacency of *prior* score *p*, including the adjacency in a solution costs −*kT*_0_ log(*p*) while discarding it from the solution costs −*kT*_0_ log(1−*p*), where *kT*_0_ is a pseudo-temperature that we discuss in details in Additional file 1.

A polynomial time and space Dynamic Programming (DP) algorithm samples solutions for a given instance with a probability proportional to their score. More precisely, ADSEQ can sample solutions under a Gibbs-Boltzmann probability distribution defined as follows: the Gibbs-Boltzmann score of a solution *S* is equal to *exp*^−*n*(*S*)/*kT*^, where *kT* is a user-defined pseudo-temperature, and this score defines implicitly a probability distribution over the set of all solutions. Tuning the pseudo-temperature *kT* provides a control over the probability to sample parsimonious solutions: a low pseudo-temperature tends to increase the probability to sample most parsimonious solutions while a large pseudo-temperature skews the Gibbs-Boltzmann distribution toward the uniform distribution over the set of all solutions (we refer to [51] for more details on the Gibbs-Boltzmann framework applied to the DECO algorithm).

In the present work, the *prior* scores of extant adjacencies are either 1.0 for adjacencies that are observed in a contig or scaffold, or a scaffolding score obtained from sequencing data using the scaffolding software BESST. Pairs of genes located at the extremities of contigs and for which sequencing data do not provide any evidence for a scaffolding adjacency receive a small prior score as described in [52] (see also Additional file 1). The *posterior* scores are defined as the frequency out of a sample of 100 solutions with temperatures *kT*=*kT*_0_=0.1 that skews the Gibbs-Boltzmann distribution toward optimal solutions.

#### Linearization of extant and ancestral components

After ADSEQ is applied to all considered pairs of gene families, the obtained result is a set of ancestral and extant gene adjacencies, each adjacency being assigned a *posterior* score. False positives – i.e. pairs of genes predicted inaccurately to be contiguous in an extant or ancestral genome–, can be due to errors in the data (for example errors in gene families or reconciled gene trees) or to errors in the inference process (for example the parsimony assumption might be wrong for some gene adjacencies). As a result, it is possible that a given gene (or gene extremity) belongs to more than two (more than one) adjacencies, which is incompatible with the expected linear structure of chromosomes.

**Fig. 2:**
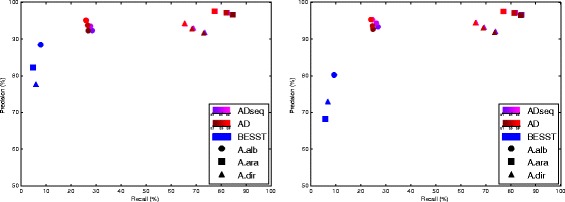
Precision and recall statistics for scaffolding adjacencies on three artificially fragmented genomes (A.alb: *A. albimanus*, A.ara: *A. arabiensis* and A.dir: *A. dirus*). **Left graph:** results with 50% of reads. **Right graph:** results with all reads. The different methods results are plotted with the precision on the Y axis and the recall on the X axis. For ADSEQ and AD, results for three different adjacency support thresholds (0.1, 0.5 and 0.8) before genome linearization are plotted and represented with a color gradient. Note: A True Positive (TP) adjacency requires the proper orientation of both genes

To address this issue, we process the set of ADSEQ ancestral and extant adjacencies in such a way that they define linear ancestral and extant segments. To do so, we apply, independently for each species, a method used both in ancestral genome reconstruction [55] and scaffolding algorithms [56]. It consists in extracting, for each species, a Maximum-Weight Matching (MWM) in the graph whose vertices are gene extremities and edges are gene adjacencies, weighted by their posterior score. This MWM can still include circular segments, that are linearized by removing the least-weight edge of each such circular segment. Moreover, prior to this linearization step, we discard adjacencies whose posterior score is below a user-defined threshold, that was set to 0.1 after simulations aimed at measuring the accuracy of the ADSEQ algorithm (see Fig. 2). This linearization step is done independently for each species, ancestral or extant.

### Application to the *Anopheles* data set

We now describe how we generated the data for the 18 *Anopheles* genomes.

#### Species trees

**Fig. 3:**
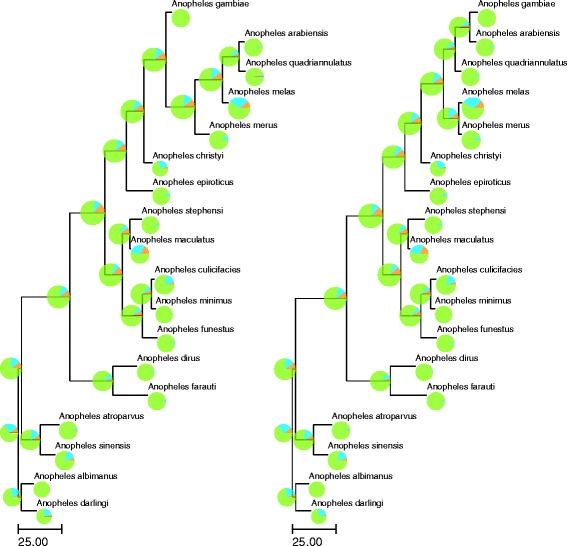
A. species trees (left: X phylogeny, right: WG phylogeny) with rearrangements per adjacency as branch lengths (× 10^−3^). The pie chart for a given species represents the adjacency degree of the genes of this species: orange represents genes with no adjacency, light blue genes with adjacency degree of 1 and green genes with adjacency degree of 2. Moreover, the diameter of each chart is proportional to the number of genes in the corresponding species

The main species phylogeny we considered was taken from [5]. We call it the “X phylogeny” as it is based on the X chromosome genes. It is the species tree used by default unless another is specified. We also considered an alternative “Whole Genome (WG) phylogeny”, that was introduced in [5] as the most frequently observed among trees built using sequences from the autosomes (Fig. 3).

#### Genomic data

Most genomic data we used were produced by the *Anopheles 16 Genomes project* described in [4] and retrieved from VectorBase (https://www.vectorbase.org): genome assemblies (contigs, scaffolds, or chromosome arms), gene annotations, and gene sequences (CDS). For 16 out of the 18 considered *Anopheles* species, we retrieved from the NCBI Sequence Read Archive (SRA) paired-end Illumina libraries with an insert size of 180bp (’fragment’ libraries) and mate-pair libraries with an insert size of 1.5kbp (’jump’ libraries), both obtained from a single female individual. Additional low-coverage long-range sequencing libraries (’Fosill’ libraries) were obtained from pools of individuals. Details are available in Additional file 17: Table S1.

#### Gene families and trees

We retrieved homologous gene families from orthologous gene groups of 21 *Culicidae* species recovered from the OrthoDBmoz2 database (http://cegg.unige.ch/orthodbmoz2) generated using OrthoDB [57]. We generated a multiple sequence alignment for each family, used RAXML [58] to compute draft gene trees with bootstrap supports, and then corrected these draft gene trees using PROFILENJ [59]. PROFILENJ contracts branches with low bootstrap support and, using the species tree, resolves the polytomies in a way that minimizes the number of duplications and losses in a reconciliation. This resulted in 14,940 gene trees containing 183,680 genes. We refer to Additional file 1, Additional file 2: Figure S1, Additional file 3: Figure S2, Additional file 4: Figure S3, and Additional file 5: Figure S4, and Additional file 18: Table S2 for a description of our preprocessing pipeline (including preprocessing of these gene families), a comparison of the newly inferred gene trees with the original ones computed by the *Anopheles* consortium and for statistics on the inferred gene duplications and losses in these gene trees. As a species phylogeny is required to reconcile gene trees, we repeated the process described above for both the X phylogeny and the WG phylogeny.

#### Extant adjacencies and prior scores from sequencing data

Sequencing reads from all libraries were filtered to discard low quality reads and trimmed to 75bp length using TRIMMOMATIC [60], then mapped onto the contigs or scaffolds of the considered species using BOWTIE2 [61]; for reads with multiple mappings, all of them were conserved. The scaffolding software BESST [62, 63] was then used to detect potential scaffolding adjacencies between pairs of contigs containing at least one annotated gene. Scaffolding adjacencies that were not supported by at least four pairs of reads were discarded. For remaining scaffolding adjacencies, we assigned a score defined as the arithmetic mean of the two scores computed by BESST, the link variation score and the link dispersity score. Detailed statistics on the scaffolding adjacencies so obtained are available in Additional file 6: Figure S5 and Additional file 7: Figure S6.

### Genome fragmentation simulations for measuring the accuracy of ADSEQ for extant scaffolding

We developed a validation protocol of ADSEQ to measure its ability to propose reliable extant scaffolding adjacencies (see Additional file 8: Figure S7 for an illustration of the protocol and Additional file 19: Table S3 for assembly statistics at different steps of the protocol). The key element is to provide to ADSEQ a genome whose assembly is more fragmented than the reference assembly, in order to verify that ADSEQ can retrieve the lost adjacencies. Moreover our simulation framework aims at generating a realistic fragmentation, as relying on a random fragmentation, as used in other validation protocols [16, 52], generates data that are in general easy to scaffold using comparative methods.

To avoid this pitfall we simulated a fragmented assembly by re-assembling the considered genomes using KMERGENIE [64] and MINIA [65]. MINIA was chosen due to its stringency in handling repeats, that leads to more conservative and fragmented assembly compared to other contig assemblers. We applied this protocol to a randomly chosen either all or half of the raw sequence reads, independently three times, with the species *A. albimanus*, *A. arabiensis* and *A. dirus*, whose positions in the species tree allow to consider various evolutionary contexts.

We ran ADSEQ as described above on these new assemblies and compared its results (scaffolding adjacencies) with the reference assemblies. We call a True Positive (TP) adjacency an adjacency inferred by ADSEQ and present in the initial genome assembly. A False Positive (FP) adjacency corresponds to an adjacency inferred by ADSEQ and not present in the reference genome assembly. A FP can however be a true adjacency not found by the reference assembly (e.g., connecting two scaffolds), so we call Certain False Positive (CFP) a FP adjacency which extremities are not scaffold or contig extremities in initial genome assembly. Finally a False Negative (FN) is a pair of gene extremities that are contiguous in the initial assembly but are not inferred as an adjacency by ADSEQ. From these values we compute the usual *precision* and *recall* statistics, but using CFP for the false positives count.

### Gene order evolution analysis

#### Assignment of chromosome segments

To compare the evolution of *Anopheles* chromosomes, especially the apparent differences between the X chromosome and the autosomes described in [4], it is necessary to assign extant and ancestral chromosome segments to either the X chromosome or the autosomes. As *A. gambiae* is the only fully assembled genome in our data set, this is also the only genome for which such information is readily available; in all other species the genomes are assembled into scaffolds with no indication of whether this scaffold belongs to the X chromosome or an autosome, unless additional data is available, such as genome maps. We assigned extant and ancestral genes and segments to the X chromosome or autosomes using the following probabilistic method. For each gene *g* (ancestral or extant), a set of *An. gambiae* orthologs is defined as all *An. gambiae* genes from the same gene family than *g* whose last common ancestor with *g* in the reconciled gene tree is a speciation node. Note that this set might be empty, and that this definition includes the case where *g* is an ancestor of a *An. gambiae* gene. The probability of *g* being on the X chromosome is then defined as the frequency of orthologs located onto *An. gambiae* X chromosome, or, if no ortholog is present on this X chromosome, by a background probability, defined as the global frequency of *An. gambiae* genes on the X chromosome. Then each segment is given a probability to be located on the X chromosome as the mean of probabilities for all genes it contains. Each gene then inherits the probability of being on the X chromosome from the segment it belongs to.

Very recently an assignment of *An. albimanus* genes to chromosomes was published together with a new assembly [66] that we could use to verify that our assignment method is accurate: out of 8840 genes assigned to a chromosome in the novel assembly, we correctly predicted the autosomal/X placement of 8837 genes (comparing the assignment of higher probability and the assignment in the new assembly).

#### Gene movements (translocations)

For every couple of genes for which one is a direct descendant of the other, we inferred a gene movement (between the X chromosome and an autosome) if the probability of the ancestral gene being on the X chromosome is ≤0.2 while the probability of the descendant gene being on the X chromosome is ≥0.8, or conversely.

#### Detecting chromosomal rearrangements

For every branch of the species tree, genes with exactly one exemplar in their family both in the ancestor and descendant species were selected. Then conserved adjacencies were computed, which are adjacencies present between ancestral genes and descendant homologous genes. In order to discard gene displacements, which are counted elsewhere, we filtered also genes which are not involved in any common adjacency. A *rearrangement* (gain or breakage of a gene adjacency) is counted every time two gene extremities are contiguous on a segment (with respect to the reduced selected set of genes) of the ancestor, but not on the descendant, or conversely. When they are not contiguous, in order to detect a certain genome rearrangement, we require also that they are not both the extremities of their segments, to avoid counting as a rearrangement a potentially undetected scaffolding adjacency. Rearrangements were not directly counted as gains and breakages output from ADSEQ because this count can be blurred by adjacencies gained or broken by gene duplications and gene losses. As a consequence, gene duplications and losses are not counted as rearrangements, that are limited to synteny breakages due to balanced rearrangements (inversions, transpositions, large translocations), that do not change the gene content.

We stress that this method to detect genome rearrangements is conservative and underestimates rearrangement counts, as it does not detect the rearrangements hidden by the assembly fragmentation of the considered genomes. Moreover, this underestimation can be biased by the degree of fragmentation of the compared species, so two numbers of rearrangements are not necessarily comparable in biological terms, even for species closely located in the species phylogeny. However, given the same genomes in the input, the numbers of rearrangements for two different phylogenies are comparable, as are the number of rearrangements in sex chromosomes and autosomes.

## Results

The results are organized in three parts. First, we describe the results of the simulation-based evaluation of the accuracy of ADSEQ to recover extant scaffolding adjacencies. Then we describe extant and ancestral genomes obtained with ADSEQ, and analyze important aspects of their evolutionary history. Finally we use ADSEQ to evaluate different species trees and re-examine the conclusions of [5] regarding species evolution.

### Validation of the ADSEQ algorithm for extant scaffolding

We compared the scaffolding performance of ADSEQ with two other methods, on the same data set of realistically fragmented genomes (see Methods). One is using sequence information only, BESST [63], while the other one is using the comparative approach only, on the same phylogenomic data, AD (ADSEQ, where the possibility of using sequence information is turned off). Figure 2 shows the precision statistic in function of the recall statistic for the two data sets (sampling 50% of the reads or using them all). For ADSEQ and AD, different values of the threshold for filtering adjacencies prior to linearization (see Methods) were tested.

ADSEQ outperforms BESST in precision and recall independently of the threshold. This shows that in the majority of cases, adding phylogenomic information improves the recall without affecting the precision compared to a method purely based on sequence data. Additional results, where the gene orientation is not considered to determine an inferred adjacency as TP, are provided in Additional file 9: Figure S8 et Additional file 10: Figure S9. These results show that BESST has an equivalent precision and recall statistics compared to results of Fig. 2 where gene orientation is considered. This comparison shows that for most of inferred adjacencies the three methods inferred the right orientation for both genes involved in adjacency. In summary the combined approach using comparative signal and sequence data (ADSEQ) is giving significantly better results than a method based on sequence alone (BESST).

We now compare ADSEQ and AD, for a better illustration of the Fig. 2 to compare precision and recall statistics between ADSEQ and AD see subfigures zooming in Additional file 11: Figure S10. Recall statistic is slightly better with ADSEQ than with AD for all considered threshold values. For most of the condition the precision statistic is slightly better ADSEQ than AD (except in some conditions for *A. albimanus* and *A. dirus*, see Additional file 11: Figure S10). For *A. albimanus*, with all reads considered, ADSEQ outperforms AD for recall statistic for a threshold fixed to 0.1 and 0.5. So from a quantitative point of view, adding sequence data seems to have a smaller impact on the recall and precision statistics compared to using synteny evolution. Note however that the combination of both supports for extant scaffolding adjacencies (sequence data and synteny evolution) is an important by-product of ADSEQ. A phylogenetic method alone is more difficult to trust in the absence of sequence data. So even if the general statistics are comparable, the additional support brought by the sequence data is an important feature. Moreover, additional results in Additional file 12: Figure S11 and Additional file 13: Figure S12 strongly support that a joint combination of phylogenetic and sequence signals (ADSEQ) overpasses an *a posteriori* combination of phylogenetic signal and sequence data (AD + BESST) for scaffolding improvement. These results show indeed that combining AD + BESST slightly overpasses ADSEQ in term of recall statistic (stronger TP adjacencies) but at the expense of a strong decrease of the precision.

### Improved scaffolding of *Anopheles* extant and ancestral genomes

**Fig. 4:**
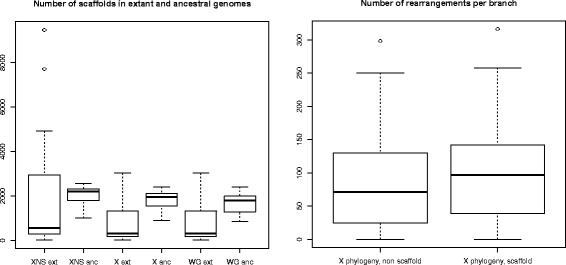
**Left:** Number of segments in extant and ancestral genomes, according to three runs of ADSEQ in three different conditions. In the first run, we turn off the scaffolding mode on the X phylogeny, that is, it only constructs ancestral segments. The first column “XNS ext” thus describes the initial assembly, and “XNS anc” the assembly of ancestral genomes when reconstructed without extant scaffolding. In the second and third runs, the scaffolding mode was turned on, and run with the X phylogeny (“X ext” and “X anc”) and the WG phylogeny (“WG ext” and “WG anc”). **Right:** Number of rearrangements over all branches of the X phylogeny, with and without the scaffolding mode

Properties of the improved assemblies for *Anopheles* extant genomes and of the reconstructed *Anopheles* ancestral genomes segments are summarized in Fig. 4. We describe three runs of ADSEQ: one without proposing extant scaffolding adjacencies (which amounts to use the DECLONE algorithm [51] to reconstruct ancestral genomes without improving extant genomes) and two with ADSEQ using the X and WG species phylogenies. The first observation that can be made is that the ability to create extant scaffolding adjacencies has a very significant impact on the ability to reconstruct ancestral segments, that define ancestral genomes at a similar level of fragmentation than the improved extant genomes. This effect is important toward refined genome evolution analysis that rely on the ancestral segments as input material, especially to detect chromosomal rearrangements.

Additional file 20: Table S4 and Additional file 14: Figure S13, Additional file 15: Figure S14, and Additional file 16: Figure S15, provide more detailed illustration and statistics on the improved scaffolding. We observe that from 36,634 initial extant segments (contigs, supercontigs and scaffolds after the various filterings steps described in Methods and SI text), we scaffold the extant genomes into 13,525 segments, with an average number of 94 genes per segment up from 37 before running ADSEQ. Very similar results are obtained for all genomes independently of the chosen phylogeny, confirming the overall picture described in Fig. 4-Left.

On the right side of Fig. 4, we can observe that we retrieve a significantly (Wilcoxon paired test, *p*-value < 10^−4^) higher number of rearrangements by the joint scaffolding technique than by just constructing ancestral genomes without scaffolding extant genomes. So the joint scaffolding of extant and ancestral genomes is beneficial to both. In particular scaffolding extant genomes while reconstructing ancestral genomes gives access to more information regarding the evolutionary history.

An interesting feature of ADSEQ is the possibility that the linearization step does delete an observed gene adjacency in an extant genome. This is unlikely as observed adjacencies have the highest score in the linearization procedure, but it can happen if it is in conflict with other adjacencies with high posterior probability. It happened only once in our dataset, for an adjacency between two *A. culcifacies* genes. The two genes were predicted in the reverse order, or equivalently in the reverse orientation, because all identified homologs were arranged similarly. This can be explained either by two inversions, one encompassing each gene, or by an assembly or annotation error. This shows that our approach can also detect questionable adjacencies in the given extant assemblies.

### Evolution and phylogeny

ADSEQ is not a phylogenetic method *per se*, as it requires a given species phylogeny and does not include an extension to search an optimal phylogeny according to some evolutionary criterion. However as a method which infers ancestral gene orders and evolutionary events, and is computationally efficient (all steps that require a species phylogeny, including the correction of gene trees with ProfileNJ, the reconciliation of the gene trees with the species trees, and the joint scaffolding / ancestral genome reconstruction takes five hours on a laptop), it can be used to compare a few selected competing phylogenies. To this aim we compared several measures obtained by the same methods using the two phylogenies, WG and X. They are the two main examined topologies in [5], where X is preferred as depicting the species history.

#### Duplications

Our pipeline using PROFILENJ to correct gene trees allows to record gene duplications. We counted a total of 6461 duplications for the X phylogeny, against 6159 duplications for the WG phylogeny (see Table 1). This means that for many gene families, a duplication was identified in the X phylogeny and not in the WG phylogeny. For these families, a well supported branch (100% bootstrap with RaxML) was compatible with the WG phylogeny but not with the WG phylogeny, indicating that well supported branches are more often compatible with the WG phylogeny. This supports the result of [5] that most genes follow the WG phylogeny. The fact that this is observed on the autosomes and not on the X phylogeny also supports that the genomes evolve with two compartments.

**Table 1:** Numbers of inferred rearrangements and duplications in the X chromosome and in the autosomes, according to the phylogeny (X or WG) used as a parameter of ADSEQ

Event	X phylogeny		WG phylogeny	
	X chr.	Autosomes	X chr.	Autosomes
Duplications	604	5857	606	5553
Rearrangements	415	2949	416	2760

#### Scaffolding and ancestral genome reconstruction

On the left side of Fig. 4, we can observe a first difference between the results obtained with the X and the WG phylogenies: the extant scaffolding is slightly better (in terms of fragmentation level of the extant genomes) with the X phylogeny (mean segment number is 835 genes for the X phylogeny, versus 840 for the WG phylogeny), while the ancestral scaffolding is better with the WG phylogeny (mean segment number is 1860 for the X phylogeny, versus 1756 for the WG phylogeny). The better extant scaffolding with the X phylogeny can be attributed to the basal position of the genome with best assembly (*A. gambiae*) in the *Gambiae complex*. Indeed in ADSEQ the sister species can be assembled according to *A. gambiae*, but outgroup species cannot, so the assembly is necessarily better if a fully assembled genome has more sisters species and less outgroups as it is the case with *A. gambiae* in the X phylogeny. Interestingly ancestral genomes are better scaffolded with the WG phylogeny, even with this sister-branch artifact that concerns extant genomes. This better ancestral genome reconstruction obtained with the WG phylogeny could be considered as a first signal contradicting the hypothesis that the X phylogeny is the true species phylogeny, although it does not allow to draw definitive conclusions.

#### Conflict

With both phylogenies we also measured the level of syntenic conflicts, defined as the sum of the posterior scores of the adjacencies discarded during the linearization phase (data shown in SI text). We observe a higher level of syntenic conflicts in the X phylogeny (7665) than in the WG phylogeny (6319). According to simulations (described in SI text), the level of conflict is higher with a wrong phylogeny, even if it is not with the same order of magnitude than what we observe on our data. This could be seen as a second element contradicting the sequence-based hypothesis that the X phylogeny is the true species phylogeny, although the high level of conflict observed with both phylogenies here again does not allow to draw reliable conclusions.

#### Gene movements (translocations)

As it is believed there have been no large-scale rearrangement between the X chromosome and the autosomes in the *Anopheles* history [4, 67], we could assign most extant and ancestral segments (at least almost all that contain more than one gene) either to the X chromosome or to an autosome, with high accuracy (see Methods). Then we identified which genes moved from the X chromosome to an autosome, or conversely, by screening all couples of direct ancestor/descendant genes, one being in a segment assigned to the X chromosome and the other to an autosome. We found 429 genes having moved from the X chromosome to an autosome, and 469 from an autosome to the X chromosome, which confirms the trend found by Neafsey et al. [4] (59 over 132 gene movements originated from the X chromosome), although we draw our conclusion from experiments using now many more genes than in [4].

#### Genome rearrangements

We now turn to detecting genome rearrangements, as defined in the Methods section. In particular, we stress that we look for breaks and gains of gener adjacencies due to genome rearrangements such as inversions, transpositions and translocations, excluding duplications and losses, as well as adjacency breaks and gains due to duplications and losses. Moreover, due to the fragmented nature of many ancestral genomes, we expect to underestimate the true number of synteny breaks and gains.

We detect 3364 gains or breakages of adjacencies (860 in the *Gambiae complex*) using the X phylogeny, and 3176 using the WG phylogeny (590 in the *Gambiae complex*). The difference is illustrated in Fig. 3. Between the two competing phylogenies, one can observe a 30% decrease in the number of rearrangements within the *Gambiae complex* with the WG phylogeny compared to the X phylogeny. These gains/breaks of adjacencies can be combined along each branch to detect inversions, defined by pairs of breaks in the ancestor and pairs of gains in the descendant involving the same four gene extremities. This lead to the identification of 242 inversions in the X phylogeny (including 16 inversions in the *Gambiae complex*, including 4 on the single lineage to *A. gambiae*) and 240 inversions with the WG phylogeny, with only 4 in the *Gambiae complex*, two of them on the branch leading to *A. gambiae*.

#### Comparison of sex chromosomes and autosomes evolution

Sex chromosome and autosomes have different evolutionary modes, according to duplications and rearrangements. Table 1 summarizes the number of inferred events of gene duplication and genome rearrangement in the sex chromosomes and autosomes, depending on the chosen phylogeny.

A striking observation is the different behavior of the X chromosome and of the autosomes regarding duplications and genome rearrangements. We do not count loss events to compare phylogenies because the absence of genes can be due to the fragmented assembly and not necessarily to actual gene losses during evolution. This compartmentalization was observed by [5] for genes and attributed to introgression in the autosomes; it was also noticed in [4] that the genome rearrangement rate was much higher in the X chromosome than in autosomes. We observe here a similar trend. We computed genome rearrangement rates by normalizing the number of observed gains and breaks of gene adjacencies by the number of gene adjacencies in the whole set of extant and ancestral genomes; with the X phylogeny we could observe that the X chromosome has a rearrangement rate equal to 1.46 times the rate observed in the autosomes, a figure that is similar (1.57) using the WG phylogeny. The observed higher reate of rearrangement in the X chromosome is in fact likely higher, as the relative fragmentation of the chromosome X is higher compared to the autosomes in most species both extant and ancestral (data not shown).

Moreover, we can observe interesting differences between the X and WG phylogenies. Constantly less events are found on the X chromosome with the X phylogeny, while less events are found on the autosomes with the WG phylogeny. It seems indeed that not only do genes follow different histories because of introgression [5], but also entire chromosomes do. However, the observed compartmentalization alone does not allow us to specify which part of the genome has followed the species diversification. As the *Gambiae complex* is estimated to be 2.2 million years old, it is reasonable to use parsimony arguments concerning rearrangements (see argument in the next paragraph). If we do so, we find less rearrangements in total in the WG phylogeny: even normalized by the number of adjacencies (because an increase in the number of rearrangements might be the effect of a higher number of adjacencies): 9.15 10^−3^ for the WG phylogeny versus 9.68 10^−3^ for the X phylogeny. This means that rearrangements do not yield the same phylogenetic signal than the one suggested in [5]), which puzzles the evolutionary scenario in the *Gambiae complex* [68].

#### Assessing the relevance of rearrangements parsimony

The fact that parsimony can give a good account on the phylogeny can be questioned. Indeed, rearrangements in *Anopheles* are not uniformly distributed [69], they can show some degree of convergence, and rearrangements can show inter-species polymorphism. To test whether in the *Gambiae complex* we are in the domain of validity of parsimony, we compared the gene order of *A. gambiae* with *A. albimanus*, which, following the recent improved assembly of *A. albimanus*, are the two genomes which have their genes assigned to chromosomes. We selected all genes with an assignment to a chromosome, and applied the EM2 distance estimator [70]. It is based on a non uniform model of genome rearrangements which has proved to give the most reliable results on mammalian genomes, whose evolution spans a similar amount of time than the *Anopheles* genomes. We found an estimation of 1313 inversions with the statistical estimator, while the parsimony solution was 1300 (data not shown). So the parsimony result is within in the 1% interval of the statistical method, far from saturation. As *A. gambiae* and *A. albimanus* are separated by approximately 79 million years of evolution, we may suppose that, in the 2 or 3 million years that have shaped the *Gambiae complex*, rearrangements were not numerous enough to contradict parsimony.

## Discussion

An important contribution of our work is the unification of two domains of research, namely genome scaffolding, and the evolution of gene order and ancestral genome reconstruction. They are usually separated despite the similarity of their objectives (reconstructing ancestral gene order is akin to a scaffolding procedure if ancestral genes are considered as contigs). Our work improves on previous works (especially [37, 39, 71]) in several aspects. In particular, we integrate elements coming from more traditional phylogenetic methods, such as gene trees and reconciliations, in order to be able to handle a large gene complement that includes gene families with complex evolutionary histories. Another important aspect of our work is the validation procedure of the scaffolding method. We propose a novel simulation procedure which takes real sequence data but lowers the coverage and rely on a conservative contigs assembler to obtain a realistic fragmentation. Using this validation method, we show that combining both sequence data and comparison with related genomes in a phylogenetic context produce better scaffolds, at least in the context of *Anopheles* genomes.

The benefit of the joint approach that considers in the same framework scaffolding extant genomes and reconstructing ancestral genomes is evident from both the improved extant genomes assemblies, where we reduce the fragmentation from roughly 36,000 segments to below 14,000 segments, and the detection of genome rearrangements, where we observe again a much better resolution of ancestral genomes. This allows us to detect a statistically significantly larger number of genome rearrangements that can not be confused with assembly artifacts. To the best of our knowledge, ADSEQ is currently the only method that can process such a data set with many genomes, most of them provided with fragmented assemblies, while using a large complement of gene families without being limited by the nature of the evolution of these families in terms of duplications and losses, and using also sequencing data. Regarding extant genomes scaffolding, the quality of our results depends of a set of factors, such as the quality of the initial extant assemblies and the position in the species phylogeny; we do not gain much for well assembled species such as *A. albimanus* which is almost an outgroup, while we refine very well the assembly of the genomes of species such as *A. minimus* or *A. dirus*. It is important to note that while we rely on sequencing data in the present work, other sources of data such as genome maps for example could be used to define a prior score for scaffolding assemblies. In terms of genome rearrangements, we likely underestimates their actual number due to the fragmentation of the reconstructed ancestral genomes. This is a consequence the very conservative approach we follow that detects only rearrangements for which there is a clear support. It remains to see if more realistic models of genome rearrangements that do not rely on reconstructed ancestral gene orders would be able to cope, in terms of computational complexity and of robustness of the detected rearrangements, with both the large number of species considered here and the level of fragmentation of the extant genomes assemblies. Nevertheless, the results we obtain support strongly the observation of [4] that the X chromosome evolves by genome rearrangements at a much higher rate than the autosomes.

## Conclusions

Genes sequences and nucleotide substitutions are questionable markers for phylogeny because of introgression, due to hybridization or horizontal transfer, as it has been several times demonstrated recently, and in particular on *Anopheles* species. Genome rearrangements can help decipher phylogenetic relationships. However, they are difficult to infer from genomes with fragmented assemblies, a common feature for genomes obtained from short sequencing reads. We introduce a computational and statistical method for the inference of genome rearrangement events in the presence of fragmented assemblies, proposing improved scaffolding based on sequencing data and an evolutionary model of gain/breakage of genes synteny along a given species phylogeny. We provide an evolutionary study of rearrangements, providing alternative hypotheses on the introgression process in the *Gambiae complex*.

Finally, our work opens the way to several research avenues. Generally, our general approach that relies on the joint analysis of sequencing data and the comparative approach to improve the quality of extant genome data could be extended to correct other types of errors that assembly breakpoints. To cite a specific example, it could be extended to account for the well known problem of unassembled genes [72], that create apparent gene loss and rearrangement breakpoints. Other avenues could include the development of metrics to compare alternative species phylogenies or the introduction in the evolutionary model of introgression events.

## Additional files

Additional file 1 Supplementary text. (PDF 181 kb)

Additional file 2 **Figure S1.** Extant and ancestral genome gene content (left) and ancestral gene degree (right). Left: Number of genes of extant species (left), ancestral species using the reconciled VectorBase gene trees (middle), and ancestral species using the reconciled ProfileNJ gene trees (right). Right: Gene degree distribution of ancestral genes after applying ADseq with the RAW gene trees (blue graph) and the ProfileNJ gene trees (red graph), compared to the expected gene degree distribution for theoretical perfectly assembled genomes (black graph). The degree of a gene is defined as the sum of the ADseq *posterior* scores of adjacencies involving this gene. Here the value at coordinate *x* is the sums of all degrees in the interval [*x,x*+1]. (PDF 92 kb)

Additional file 3 **Figure S2.** The pipeline is split into two parts: The first part (Blue one) processes genome content data to obtain extant genome adjacencies. **Step 1** detects genes that are included in other genes. In **Step 2**, gene families containing these genes are filtered out to avoid ambiguity in defining observed extant gene adjacencies. In **Step 3**, gene trees are inferred from the gene sequences, one gene tree per gene family (see Additional file 5: Figure S4 for more information on gene trees inference pipeline). Finally, in **Step 4** genes contained in gene families for which gene trees have not been inferred are discarded from the analysis (41 gene families containing representing 1,039 genes). The second part of the pipeline (Green one) processes sequencing data to obtain scaffolding adjacencies that will be used to improve extant genome assembly with the ADseq algorithm. **Step A** trims reads with Trimmomatic to remove low qualities reads and remaining adapters. In **Step B**, trimmed reads are mapped onto their respective genome with Bowtie2 considering all multiple mappings. In **Step C**, pairs of contigs for which paired-end reads suggest a possible contiguity along their chromosome are linked with the scaffolding software BESST, and the resulting potential scaffolding adjacencies are scored according to the BESST model. Then, in **Step 5** scaffolding gene adjacencies are determined from contigs adjacencies obtained from sequencing data processing part with genes present in gene trees. This results in scaffolds with observed scored scaffolding adjacencies that are used as input of DeCoSTAR (**Step 6**). See Additional file 18: Table S2 for a description per species on dataset used for DeCoSTAR. Pipeline to produce input data for ADseq on 18 *Anopheles* dataset. The pipeline takes as input a species tree for the 18 *Anopheles* species, the whole set of gene families and gene trees for these species and genomic data (contigs, scaffolds and chromosomes). The goal of the pipeline is to produce input data for the ADseq algorithm to reconstruct ancestral genome structure and evolution and to improve extant genome scaffolding. (PDF 103 kb)

Additional file 4 **Figure S3.** Distribution of gene families number (red bars) and number of genes (blue bars) per families containing *x* species. **Left graph:** distribution of the 17,780 raw input gene trees corresponding to 212,800 genes. **Middle graph:** distribution of the 14,981 gene families, containing 184,719 genes, after discarding families containing included genes (after step 2 of Additional file 3: Figure S2). **Right graph:** distribution of the 14,940 gene trees, composed of 183,680 genes, after gene trees inference pipeline (after steps 3 and 4 of Additional file 5: Figure S4). (PDF 83 kb)

Additional file 5 **Figure S4.** Pipeline to improve gene trees inference from homologous gene family. CDS sequences of genes in gene trees have been obtained from VectorBase database and homologous gene families deduced from the 14,981 gene trees resulting of step 2 of Additional file 5: Figure S4. **Step A** consists to multiple align homologous genes with Muscle with parameter “-maxiters 2” if a gene sequence have a size upper than 32,000 bp. In **Step B**, Gblocks was applied on alignments to select high confidence alignment sites. At this step, 41 gene families have been discarded due to sequences that were not present in a selected blocks. For **Step C**, RAxML have been used to infer maximum likelihood gene trees with the GTR-GAMMA model and 100 bootstrap iterations. Finally in **Step D**, the maximum likelihood gene trees are processed with ProfileNJ to potentially changing branches with bootstrap support lower than 100% in a DL reconciliation model (min(Duplication,Loss)) with the species tree [73]. (PDF 35 kb)

Additional file 6 **Figure S5.** Distributions of scaffolding adjacencies scores computed by BESST for scaffolding adjacencies supported by at least 3 paired reads. **Left graph:** adjacency scores distribution between all contigs or scaffolds, over 405,939 scaffolding adjacencies. **Right graph:** adjacency scores distribution for contigs and scaffolds with gene corresponding to the 68,876 scaffolding gene adjacencies considered by DeCoSTAR. Blue bars represent the link variation score, red bars the link dispersity score and purple bars the mean of the two link scores. For more information on the link scores see SI text and [62]. (PDF 37 kb)

Additional file 7 **Figure S6.** Distributions of scaffolding adjacencies link scores computed by BESST for scaffolding adjacencies supported by at least 3 paired reads, for each of the 18 *Anopheles* species. **Upper graphs:** distribution of scores all 405,939 potential scaffolding adjacencies. **Lower graphs:** distribution of scores for all 68,876 scaffolding gene adjacencies used as input by DeCoSTAR. **Left graphs:** distribution of link dispersity scores. **Middle graphs:** distribution of link variation scores. **Right graphs:** distribution of the mean of link variation and dispersity scores. Each color corresponds to one species and the number between parenthesis in the legend indicates the number of scaffolding adjacencies inferred by BESST for each species. (PDF 64 kb)

Additional file 8 **Figure S7.** The ADseq validation protocol. (PDF 61 kb)

Additional file 9 **Figure S8.** Precision and recall statistics for scaffolding adjacencies on three artificially fragmented genomes (A.alb: *Anopheles albimanus*, A.ara: *Anopheles arabiensis* and A.dir: *Anopheles dirus*), when gene orientations are not accounted for. **Left graph:** results with 50% of reads. **Right graph:** results with all reads. The different methods results are plotted with the precision on the Y axis and the recall on the X axis. For ADseq and AD, results for three different adjacency support threshold (0.1, 0.5 and 0.8) before genome linearization are plotted and represented with a color gradient. These results show similar results to Fig. 2 showing that for most of the predicted adjacencies the three methods infer the correct gene orientation. (PDF 106 kb)

Additional file 10 **Figure S9.** Subfigures zooming of Additional file 9: Figure S8 to compare precision and recall statistics between ADseq and AD. **Upper graphs:** zoom of results with 50% of reads. **Lower graphs:** results with all reads. (PDF 154 kb)

Additional file 11 **Figure S10.** Subfigures zooming of Additional file 3: Figure S2 to compare precision and recall statistics between ADseq and AD. **Upper graphs:** zoom of results with 50% of reads. **Lower graphs:** results with all reads. (PDF 136 kb)

Additional file 12 **Figure S11.** Venn diagrams showing adjacencies shared by the three scaffolding methods ADseq, AD and BESST with a sample of 50% of the reads. **Upper Venn diagrams:** results for *Anopheles albimanus*. **Middle Venn diagrams:** results for *Anopheles arabiensis*. **Lower Venn diagrams:** results for *Anopheles dirus*. **Left diagrams:** False Negative (FN) adjacencies, corresponding to adjacencies created by the fragmentation process and that have not been recovered. **Center diagrams:** results for True Positive (TP) adjacencies. Here, an adjacency is considered TP if the pair of genes is adjacent in the reference assembly and the orientation of genes involved in the adjacency is properly recovered. **Right diagrams:** results for Certain False Positive (CFP) adjacencies. An adjacency is determined as CFP when the pair of gene does not belong to the reference assemblies and one of the two genes is not located at a contig extremity in reference genome, or if the recovered orientation of genes is incorrect. If we consider method individually, these results show that ADseq outperforms AD and BESST with the lowest number of FN adjacencies, the largest number of TP adjacencies and the lowest number of CFP adjacencies (except for *An. albimanus* where AD has the lowest number of CFP (224 vs. 231 for ADseq). However, if we combine *a posteriori*AD and BESST, this performs better than ADseq in terms of recall (higher number of TP adjacencies) but at the expense of a strong decreases of precision (much higher number of CFP adjacencies). (PDF 20 kb)

Additional file 13 **Figure S12.** Similar to Additional file 12: Figure S11 with all reads included. (PDF 20 kb)

Additional file 14 **Figure S13.** Scatter plot exhibiting scaffolding improvement of the 18 *Anopheles* genomes by ADseq with X species tree phylogeny. Right plot is a zoom of a small part of the left graph. Each color corresponds to one species. For each species, upper part of vertical line corresponds to number of segments in initial genome assembly and lower part the number of segments after scaffolding improvement by ADseq. Circle diameter is proportional to the % of scaffolding improvement of the genome where scale is displayed in lower right part of the graphs. The X axis represent the sum of *a posteriori* scores of discarded adjacencies during linearization step representing the degree of syntenic conflicts in adjacencies prediction of ADseq (see paragraph “Conflict” in section “Results”). (PDF 56 kb)

Additional file 15 **Figure S14.** Similar to Additional file 14: Figure S13 with WG species tree phylogeny. (PDF 56 kb)

Additional file 16 **Figure S15.** Similar to Additional file 14: Figure S13 with RAW gene trees instead of ProfileNJ gene trees. (PDF 48 kb)

Additional file 17 **Table S1.** Summary of genome assemblies and sequencing data information. 16 on the 18 *Anopheles* species have been sequenced in [4] and data are available on the SRA database of the NCBI (see column 4 and 6 for BioProject and SRA ID). FASTQ files of paired sequencing data have been obtained with SRA-toolkit. After mapping of paired reads on reference genome assemblies (column 2), median insert size of libraries have been determined with package “CollectInsertSizeMetrics” of Picard Tools (v1.61) (column 7). Column “Library name” give information on the sequencing strategies employed in [4]. Where ’fragment’ library corresponds to a Paired-End library with an expected insert size of 180bp and FR orientation (→←). ’jump’ library corresponds to a Mate-Pair library with an insert size of 1.5kbp and RF orientation (←→). And ’fosill’ corresponds to a library generated from a pool of hundred mosquitoes to improve the scaffolding with an expected insert size around 38kbp and FR orientation (→←). Column 3 gives the ID of gene set used in this study. (PDF 38 kb)

Additional file 18 **Table S2.** Assembly statistics on the 18 *Anopheles* genomes. Statistics before before processing are displayed in columns 2-7 and after the pipeline to produce input data for the DeCoSTAR algorithm in columns 8-11 (see Additional file 3: Figure S2 for illustration of the data preprocessing step). For initial dataset assembly statistics, columns 2 and 3 present contigs number and N50 statistic in bp for all contigs in genome assemblies. In columns 4-7, only contigs with at least one gene are considered. Column 4 corresponds to contigs number with gene in reference assemblies. Columns 5 & 6 represent N50 statistics respectively in bp and in gene number. Column 7 represent the number of gene in reference genome assemblies. For genome assemblies used as input of DeCoSTAR, all contigs contains at least one gene. Column 8 gives the number of contigs after step 4 of Additional file 3: Figure S2. Columns 9 & 10 represent N50 statistics respectively in bp and in gene number. And column 11 represents the number of gene in genomes taken as input of DeCoSTAR. The input dataset of DeCoSTAR is composed of 14,940 gene trees (see Additional file 4: Figure S3 and Additional file 5: Figure S4 for more information on gene trees) and 68,876 gene adjacencies with sequence support (scaffolding gene adjacencies) (see Additional file 6: Figure S5 and Additional file 7: Figure S6 for more information on scaffolding adjacencies). (PDF 33 kb)

Additional file 19 **Table S3.** Assembly statistics at various stages of the gene annotation step of validation protocol of ADseq (step 4 of Additional file 8: Figure S7). For each species and annotation step, table gives different assembly statistics (column2): the number of contigs in the assembly, the size of the assembly in bp and in gene number, the N50 statistics of the assembly in bp and in gene number (if available) and gene trees number corresponding to gene present in the assembly (for the two last columns). Column 3 (Initial assembly) corresponds to the assembly statistics of reference genomes. Columns 4-8 of upper and lower table corresponds to Minia assembly statistics at different filtering step respectively with 50% reads sampling and without reads sampling. Column “initial” corresponds to assembly in output of Minia algorithm assembly. Minia contigs are then mapped on reference genome to annotate gene of reference assembly on Minia contigs. Assembly statistics after filter1 corresponds to Minia contigs that have been mapped on reference assembly with an identity and a coverage >=90*%*. Filter2 consists to keep only contig with an unique optimal alignment (to avoid uncertainty in gene annotation). Column 7 corresponds to Minia assembly statistics after merging of Minia contigs overlapping a same gene (simulating RNA-seq scaffolding). Then last column corresponds to statistics after filter3 that consists to discard gene families of genes that have not been mapped on Minia contigs. (PDF 51 kb)

Additional file 20 **Table S4.** Scaffolding statistics on the 18 *Anopheles* genomes before and after ADseq (with the X (upper table) and WG (lower table) species phylogenies). The columns 2-5 correspond to assemblies statistics before running the ADseq algorithm. Column 2 corresponds to the number of contigs in reference assemblies. The N50 statistic corresponding to the contig size where 50% of the total assembly length is comprised in contigs with size superior or equal to this value. This metric is computed with size considerd both in bp (in column 3) and in gene number (in column 4). Column 5 gives the number of genes in genome assemblies give as input to ADseq. Columns 6-9 and 10-13 represent scaffolding statistics of ADseq respectively for X chromosome species tree topology and Whole-Genome topology. Columns 6 & 10 represent scaffolds number after ADseq. Columns 7 & 11, and 8 & 12 represent N50 statistics respectively for size in bp and size in gene number. Columns 9 & 13 represent new adjacencies inferred by ADseq (#scaff adj) represent the number of new adjacencies that are scaffolding adjacencies (i.e. adjacencies with sequence signal proposed by BESST and inferred by ADseq). (PDF 56 kb)

## Abbreviations

CARs: Contiguous ancestral regions
CDS: Coding DNA sequence
CFP: Certain false positive
DP: Dynamic programming
FN: False negative
FP: False positive
MWM: Maximum-weight matching
SRA: Sequence read archive
TGS: Third-generation sequencing
TP: True positive
WG: Whole genome
