# Supplementary material for: Phylogenetic signal from rearrangements in 18 Anopheles species by joint scaffolding extant and ancestral genomes
Source: BMC Genomics. 2018 May 9;19(Suppl 2):96. doi: 10.1186/s12864-018-4466-7 (PMC5954271; doi:10.1186/s12864-018-4466-7)
Supplement: Supplementary file 1 — Supplementary text. (PDF 181 kb) [file 12864_2018_4466_MOESM1_ESM.pdf]

ADDITIONAL FILE 1 - SUPPLEMENTARY TEXT

Phylogenetic signal from rearrangements in 18 Anopheles species by joint scaffolding extant and ancestral genomes

Yoann Anselmetti[1,2], Wandrille Duchemin[2,3], Eric Tannier[2,3], Cedric Chauve[4] and Sèverine Bérard[1*]

*Correspondence:
Severine.Berard@umontpellier.fr
[1]ISEM, Université de Montpellier, CNRS, IRD, EPHE, Montpellier, France
Full list of author information is available at the end of the article

The ADseq algorithm: Gibbs-Boltzmann probabilistic framework

The sampling mode of ADseq is based on the following principle. For a given ADseq instance, let $\mathcal{A}$ be the set of all possible evolutionary scenarios that are in the search space considered by the dynamic programming algorithm. For a given scenario $A \in \mathcal{A}$, we denote by $s(A)$ its parsimony score. For a given *pseudo-temperature* kT, we define the *partition function* $Z_{\mathcal{A}}$ as follows:

$$Z_{\mathcal{A}} = \sum_{A\ in \mathcal{A}} e^{-s(A)/kT}$$

The Gibbs-Boltzmann probability of A is then

$$P(A) = \frac{e^{-s(A)/kT}}{Z_{\mathcal{A}}}$$

ADseq can sample solutions from $\mathcal{A}$ with each solution A having probability $P(A)$ to be sampled. In order to sample more frequently parsimonious or near-parsimonious scenarios, one can then tune the value of kT. While kT decreases toward 0, the probability mass of the parsimonious scenarios in the Gibbs-Boltzmann distribution increases, tending toward a uniform distribution over all parsimonious scenarios; conversely, when kT increases, the Gibbs-Boltzmann distribution evolves toward the uniform distribution over all scenarios. For extant adjacencies prediction, a *scaffolding propagation index (SPI)* parameter, allowing to propagate an extant adjacency of a species A in species B located in the same clade that species A with clade size equivalent to the value of *SPI* parameter, have been fixed to 20 (> 18) to consider synteny signal from every species during scaffolding procedure. For more information on ADseq algorithm, see [1].

Genome assemblies and sequencing data of 18 Anopheles genomes data set

Out the 18 *Anopheles* species, 16 have been sequenced genomes in [2] (see Tab. S1 for Genome assembly name). Paired sequencing data are available and were obtained from the Sequence Read Archive (SRA) of the NCBI with SRA-toolkit (see Tab. S1 for information on sequencing data). The whole set of 16 species with sequencing data have two sequencing libraries on a single female mosquito. A Paired-End library

with an insert size of 180bp (called 'fragment' library) with FR orientation (→←) and a Mate-Pair library with an insert size of 1.5kbp (called 'jump' library) with RF orientation (←→). For 11 of them, a third insert size library of ∼38kbp (called 'fosill' library) was generated from a pool of hundred mosquitoes to improve the scaffolding with a FR orientation (→←). 'fosill' library is a Paired-End sequencing of fosmid library on Illumina that uses bacterial plasmid to integrate large genome portion to produce large insert size library (see [3] for more information). ID of Gene sets used for genome annotation of the 18 *Anopheles* are given in the Tab. S1. These gene sets were built by VectorBase in collaboration with J. Craig Venter Institute and/or the Broad Institute.

Pipeline to produce input data of ADseq for the 18 Anopheles genomes data set

We developed a pipeline to process available genomic data in input data for ADseq. Our pipeline is divided in two parts, first part consists to process genome content data and second part consists to process sequencing data. The pipeline is illustrated in Fig. S2.

Genome content data processing.

The right part (blue) of the pipeline in Fig. S2 consists to take available genome content and phylogenetic data on 18 *Anopheles* dataset to determine the list of adjacencies between gene contained in gene trees.

Initial filtering of gene families.

First step consists to discard gene families for which, in at least one species, one gene is fully included within another gene, as such situation do not allow to unambiguously decide the relative position of the two genes (steps 1 and 2 of Fig. S2). This filter results in 14,981 gene families whose the content is illustrated in the middle graph of Fig. S3. For overlapping genes, there is no discarding of their gene families and adjacencies between these genes is determined by the relative position of their 5' position on the forward strand.

Computing reconciled gene trees.

To handle the issues of erroneous gene trees in the gene trees dataset produced by Neafsey *et al.* [2], we inferred new gene trees from the 14,981 gene families with the protocol described in Fig. S4). For each gene family, CDS for the genes member of the family, obtained from VectorBase, were aligned with Muscle [4] (v3.8.425), then Gblocks [5] (v0.91b) was used to select high confidence alignment sites (columns). 41 families in which some sequences were not represented in any selected site were discarded at this step (see right graph of Fig. S3 for gene and species content of the 14 940 families). Maximum likelihood gene trees were then obtained with RAxML [6] (v8.2.8) with the GTR-GAMMA model, and 100 bootstrap iterations.

The maximum likelihood gene trees so obtained were then processed with ProfileNJ [7] to correct the topology by possibly changing branches with bootstrap support lower than 100% by minimizing the number of duplications and losses in a reconciliation with the considered specie tree.

In the less than one hundred cases where ProfileNJ generated several optimal solutions, an arbitrary topology was chosen. The result is a set of 14,940 gene trees representing 183,680 genes.

The resulting unrooted gene trees were then rooted and reconciled with the species tree using ecceTERA [8]. ecceTERA is a gene tree / species tree parsimony reconciliation algorithm that associates to every ancestral gene of a gene tree a species and an evolutionary event (speciation or duplication), choosing an assignment that minimizes the number of gene duplication and gene loss induced by the assignment. Given an unrooted gene tree, ecceTERA computes the rooting of the gene tree, among all possible ones, that minimizes the reconciliation score as defined above. The algorithm of ecceTERA is an efficient dynamic programming algorithm that process an unrooted gene tree with n leaves and a given species tree with m leaves in time $O(n^2m)$.

Observed gene adjacencies were deduced from annotated genes after removing of those that were not present in gene families, as ADseq relies on gene trees to infer ancestral and extant adjacencies (step 4 of Fig. S2). For statistics on the number of contigs and gene before and after the pipeline of data preprocessing, see Tab. S2.

Sequencing data processing.

The left part (green) of the pipeline in Fig. S2 consists to process paired-sequencing data to obtain weighted potential adjacencies. These adjacencies will be taken in account by DeCoSTAR for more accurate prediction of new extant adjacencies and reconstruction of the evolutionary history of genome structure.

First step consisted to trimmed reads with Trimmomatic (v0.36) [9] (step A of Fig. S2). Then trimmed reads have been mapped with Bowtie2 (v2.2.9) [10] (step B of Fig. S2). Mapping were done on the three different insert size libraries with option allowing to take into account all alignments for each reads (see Tab. S1 for library insert size estimation from mapping). Except for *Anopheles arabiensis* & *Anopheles merus* where the 100 best alignments have been taken into account, due to excessive time computation (more than one month).

A scaffolding step is done to compute a score between contigs pairs linked with the scaffolding tool BESST (v2.2.6) [11, 12] (Step C of Fig. S2). The following parameters have been used for BESST: –print_scores -z 10000 –min_mapq 0. BESST computes the gap distance between contigs pairs linked by paired-reads for which ones the distance is inferior to $(\mu + 3\sigma)$, where μ and σ are respectively the mean and the standard deviation of the insert size library and determined by maximum likelihood estimation with GapEst [13]. Then the method compute two scores, the link variation score (π_σ) and the link dispersity score (π_ζ)) for large contigs pairs (with size superior to $(\mu + 4\sigma)$). π_σ measures of how far observed distances are from the theoretical distance (where a π_σ =1 indicates that observed distances between contigs given by paired reads are similar to the estimated gap distance by GapEst) and the π_ζ measures the similarity of reads distribution on contigs linked (where a π_ζ =1 indicates an exact similarity between the reads distribution observed on the two contigs). The number of scaffolding adjacencies and the scores distribution of these adjacencies are described in Fig. S5 and S6.

Finally, scaffolding adjacencies of annotated genome contigs pairs with more than 3 links (paired-reads) have been kept, because π_ζ score computed by BESST works

correctly for scaffolding edges supported by more than 3 links, representing 405,939 directed contigs pairs linked by 4,128,682 scaffolding edges. If we consider only contigs containing genes present in gene trees *i.e* scaffolding gene adjacencies, total count for the whole *Anopheles* dataset is 68,876 scaffolding adjacencies use as input of ADseq and linked by 846,045 paired-reads .

Analysis of the newly inferred gene trees.

To evaluate the properties of the gene trees we inferred, we ran ADseq on two sets of gene trees: the gene trees obtained from *Anopheles* consortium (called RAW trees from now) and the gene trees we inferred (called ProfileNJ trees). The results are strikingly different. For example the RAW gene trees yield 39,194 duplications, against 6,461 for ProfileNJ gene trees. Fig. S1 summarizes two ancestral genomes statistics of interest. On the left-hand side we represent the distribution of the number of genes in ancestral genomes. Some ancestral genomes can have more than 30,000 genes in the RAW dataset, three times more than the biggest extant genome. Numbers are much more reasonable with the ProfileNJ dataset. On the right-hand side, we illustrate the linearity of ancestral genomes. recall that genomes do not have to be strictly linear as an output of ADseq (before linearization). We use the distribution of degrees of ancestral genes, defined as follows: the degree of a gene is the sum of the ADseq *posterior* score (that belongs to $[0, 1]$) of all adjacencies involving this gene. We use this statistics as a measure of the linearity of the inferred genomes. The ideal gene degree distribution of a complete error-free genome assembly is illustrated in the black graph (almost all genes have exactly two adjacencies and so degree two). It appears clearly that the ProfileNJ distribution (red graph) is much closer to the ideal than the RAW distribution (blue graph).

All metrics argue in favor of ProfileNJ trees for better ancestral genome inference. However, the results show gaps between the ideal scenario and ProfileNJ values. This may be due to a reconciliation with duplications and losses, while a lot of genes introgress, phylogenetic artifacts (some wrong branches are highly supported by bootstrap), errors in multiple sequences alignments or in clustering genes into families, or false positives in ADseq ancestral adjacencies.

The ADseq validation protocol

After species selection and random sampling of sequencing data (steps 1 and 2 of Fig. S7), reads are mapped with the method Minia (v2.0.3) with default parameter (except parameter abundance-min fixed to 3) (step 3 of Fig. S7). KmerGenie (v1.7016) has been used with default parameter to determine the best kmer size value use as input of Minia (see Tab. S1 for kmer size used). In order to be able to compare the new assembly with the initial assembly, we aligned the new contigs onto the initial assembly using BLASTn (v2.4.0+) [14] with BLASTn algorithm (with -task megablast) and e-value threshold fixed to 1E-10. To transfer gene annotation from initial to the new assembly, Minia contigs have to be uniquely and confidently mapped on contigs of reference assembly (step 4 of Fig. S7). To insure these criterion, two filters have been applied. Filter 1 consists to keep only contig alignment with *identity* $>= 90\%$ and *coverage* $>= 90\%$. On the remaining contig alignments only contigs with an unique optimal score alignment (in identity and coverage) are kept

(Filter 2). Moreover, if alignments of two contigs overlap the same gene, we join them into a single contig, simulating a scaffolding step based on transcripts (Merging step). Finally, any gene family for which at least one gene does not align with the new contigs is completely discarded from the initial data and from the remaining analysis (Filter 3). See Tab. S3 for assembly statistics at the different filtering step of the MINIA contigs gene annotation. Then, MINIA contigs are scaffolded to get adjacencies with sequencing support (step 5 of Fig. S7). The pipeline is the same that those used for the analysis of the 18 *Anopheles* dataset (see Fig. S2 and SI text) at the exception of the number of read multiple alignments in BOWTIE2 limited to 50 to reduce the computation time. After scaffolding step, all input data necessary for DECOSTAR are ready. We apply DECOSTAR on input data with (ADSEQ) or without (AD) sequencing data to predict new adjacencies (step 6 of Fig. S2). Then, last part of the pipeline consists to compute precision and recall statistics on predicted adjacencies compare to adjacencies not present in MINIA contigs but in reference contigs.

Comparison of scaffolding accuracy of ADseq, AD and BESST on simulated fragmented genomes

After genome fragmentation simulation for the three selected species (*Anopheles albimanus*, *Anopheles arabiensis* and *Anopheles dirus*) and the two reads sampling (50% and 100%). ADSEQ, AD and BESST are applied on the 6 datasets (3 species x 2 reads sampling) to compare the ability of the three methods to scaffolds genome. For each condition, first step consists to determine list of adjacencies that have been occulted during genome fragmentation simulation. Set of predicted of the three scaffolding methods are compared to this list. For BESST, set of predicted adjacencies corresponds to adjacencies as output of BESST and is not limited to adjacencies for which BESST compute scores. For AD and ADSEQ, set of predicted adjacencies corresponds to predicted adjacencies after linearization of genome on adjacencies with *a posteriori* support upper or equal to 0.1, 0.5 or 0.8 (see Methods).

Authors' information

[1]ISEM, Université de Montpellier, CNRS, IRD, EPHE, Montpellier, France. [2]Univ Lyon, Université Lyon 1, CNRS, Laboratoire de Biométrie et Biologie Evolutive UMR5558, 43 Boulevard du 11 novembre 1918, 69622 Villeurbanne cedex, France. [3]INRIA Grenoble - Rhône-Alpes, 655 Avenue de l'Europe, 38330 Montbonnot-Saint-Martin, France. [4]Department of Mathematics, Simon Fraser University, 8888 University Drive, V5A1S6 Burnaby, BC, Canada.

References

1. Duchemin W, Anselmetti Y, Patterson M, Ponty Y, Berard S, Chauve C, et al. DeCoSTAR: Reconstructing the ancestral organization of genes or genomes using reconciled phylogenies. Genome Biology and Evolution. 2017;.
2. Neafsey DE, Waterhouse RM, Abai MR, Aganezov SS, Alekseyev MA, Allen JE, et al. Mosquito genomics. Highly evolvable malaria vectors: the genomes of 16 Anopheles mosquitoes. Science. 2015 Jan;347(6217):1258522.
3. Williams LJS, Tabbaa DG, Li N, Berlin AM, Shea TP, MacCallum I, et al. Paired-end sequencing of Fosmid libraries by Illumina. Genome Research. 2012;22(11):2241–2249.
4. Edgar RC. MUSCLE: Multiple sequence alignment with high accuracy and high throughput. Nucleic Acids Research. 2004;32(5):1792–1797.
5. Talavera G, Castresana J. Improvement of phylogenies after removing divergent and ambiguously aligned blocks from protein sequence alignments. Systematic Biology. 2007;56(4):564–77.
6. Stamatakis A. RAxML-VI-HPC: Maximum likelihood-based phylogenetic analyses with thousands of taxa and mixed models. Bioinformatics. 2006;22(21):2688–2690.
7. Noutahi E, Semeria M, Lafond M, Seguin J, Boussau B, Guéguen L, et al. Efficient gene tree correction guided by genome evolution. PLoS ONE. 2016;11(8).
8. Jacox E, Chauve C, Szöllősi GJ, Ponty Y, Scornavacca C. ecceTERA: comprehensive gene tree-species tree reconciliation using parsimony. Bioinformatics. 2016 Feb;.
9. Bolger AM, Lohse M, Usadel B. Trimmomatic: a flexible trimmer for Illumina sequence data. Bioinformatics. 2014;30:2114.

10. Langmead B, Salzberg SL. Fast gapped-read alignment with Bowtie 2. Nature Methods. 2012;9(4):357–359.
11. Sahlin K, Vezzi F, Nystedt B, Lundeberg J, Arvestad L. BESST - Efficient scaffolding of large fragmented assemblies. BMC Bioinformatics. 2014;15(1):281.
12. Sahlin K, Chikhi R, Arvestad L, Science C. Genome scaffolding with PE-contaminated mate-pair libraries. bioRxiv preprint. 2015;p. 1–13.
13. Sahlin K, Street N, Lundeberg J, Arvestad L. Improved gap size estimation for scaffolding algorithms. Bioinformatics. 2012;28(17):2215–2222.
14. Altschul SF, Gish W, Miller W, Myers EW, Lipman DJ. Basic local alignment search tool. Journal of Molecular Biology. 1990 Oct;215:403–410.
