# Supplementary figures and images for: Phylogenetic signal from rearrangements in 18 Anopheles species by joint scaffolding extant and ancestral genomes
Source: BMC Genomics. 2018 May 9;19(Suppl 2):96. doi: 10.1186/s12864-018-4466-7 (PMC5954271; doi:10.1186/s12864-018-4466-7)

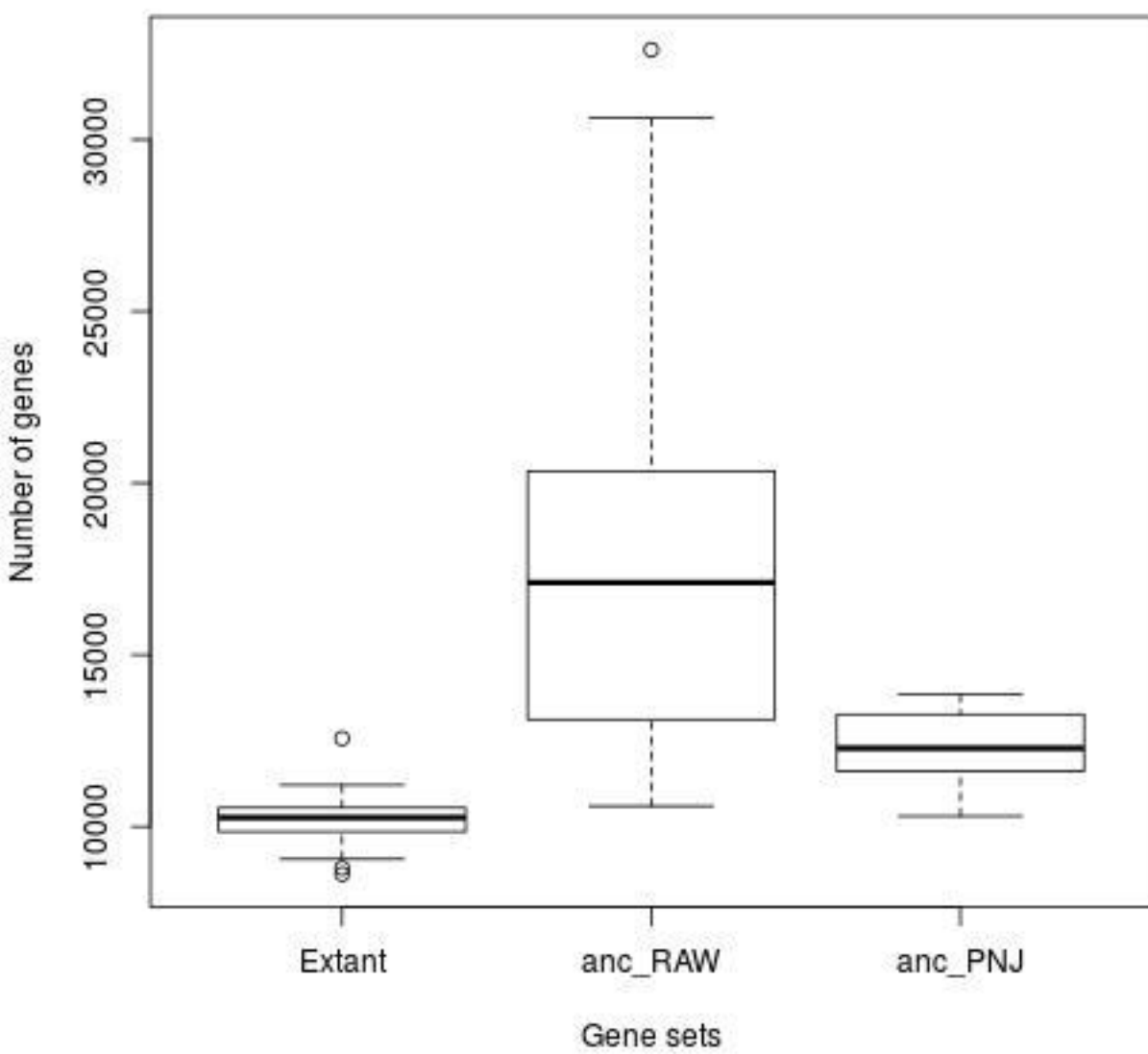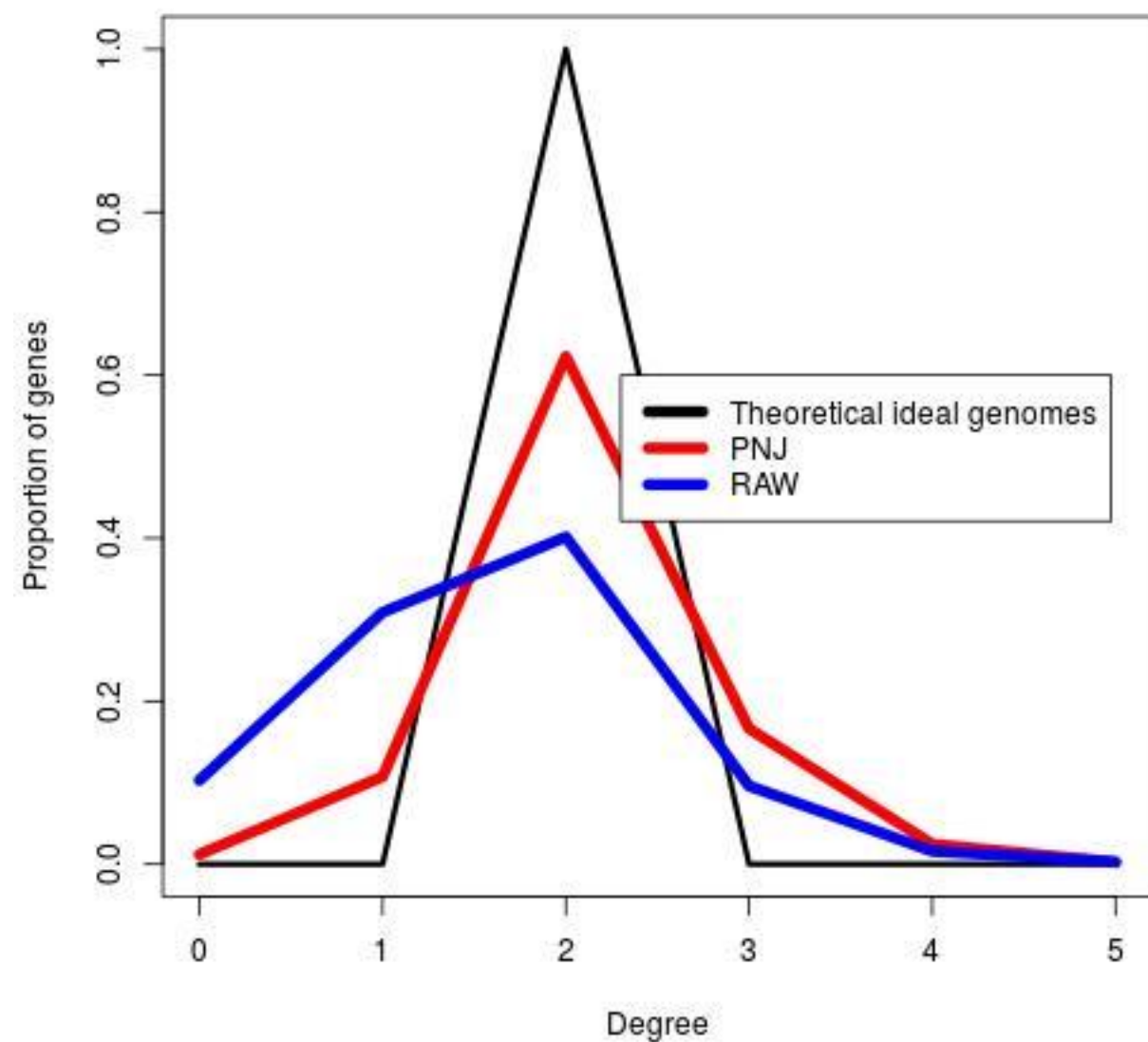

Supplement: Supplementary file 2 — Figure S1. Extant and ancestral genome gene content (left) and ancestral gene degree (right). Left: Number of genes of extant species (left), ancestral species using the reconciled VectorBase gene trees (middle), and ancestral species using the reconciled ProfileNJ gene trees (right). Right: Gene degree distribution of ancestral genes after applying ADseq with the RAW gene trees (blue graph) and the ProfileNJ gene trees (red graph), compared to the expected gene degree distribution for theoretical perfectly assembled genomes (black graph). The degree of a gene is defined as the sum of the ADseq posterior scores of adjacencies involving this gene. Here the value at coordinate x is the sums of all degrees in the interval [x,x+1]. (PDF 92 kb) [file 12864_2018_4466_MOESM2_ESM.pdf]

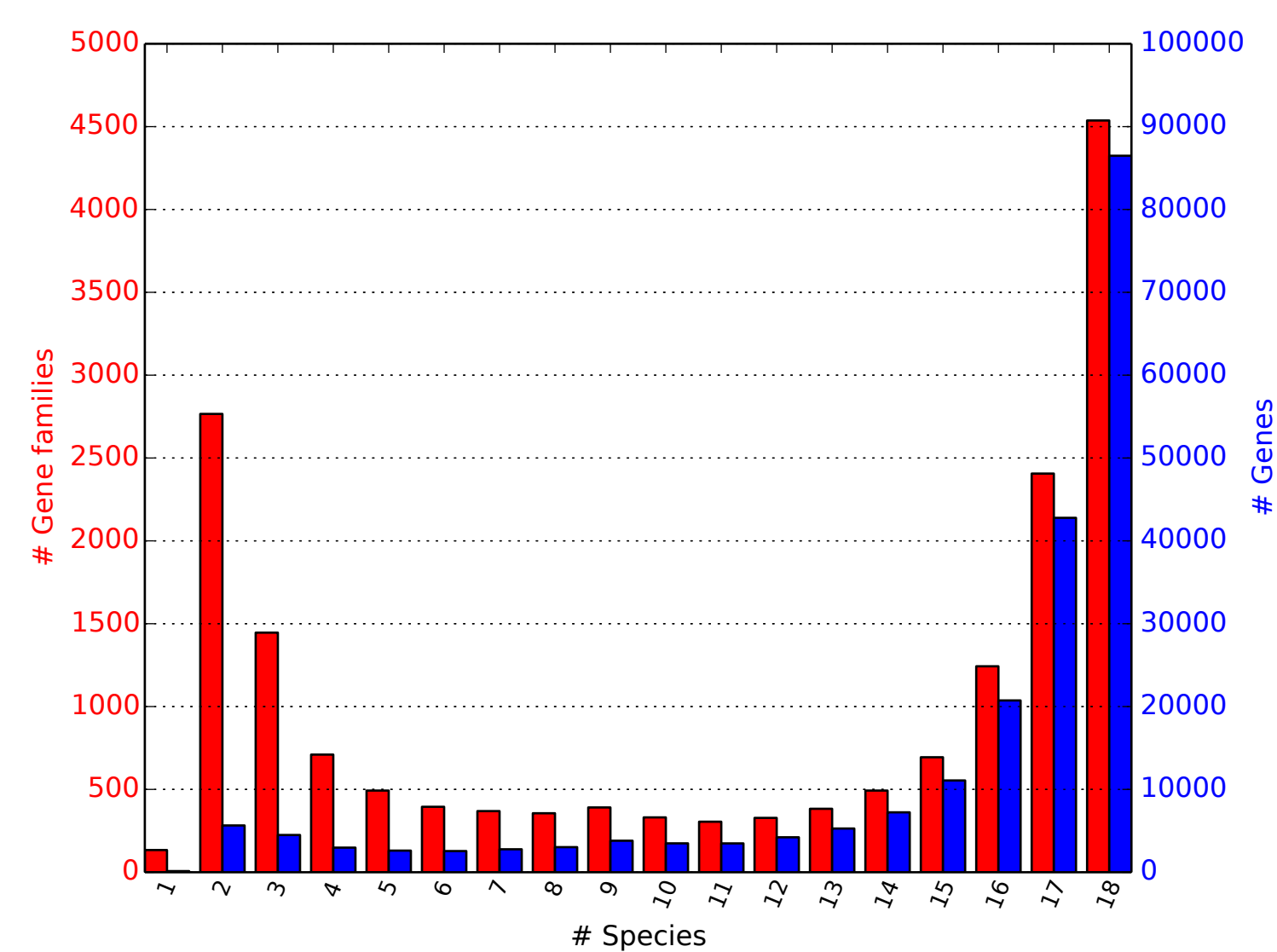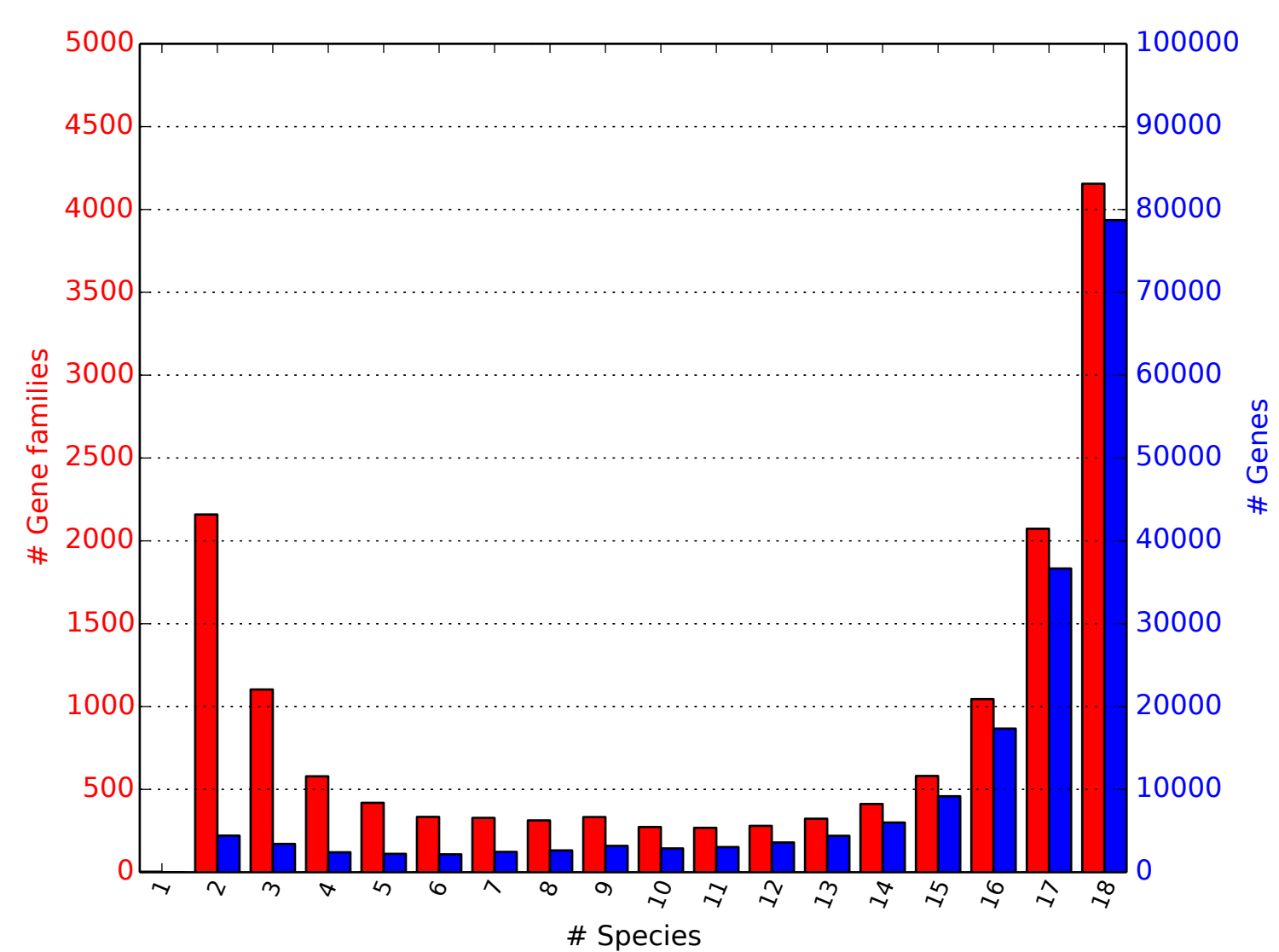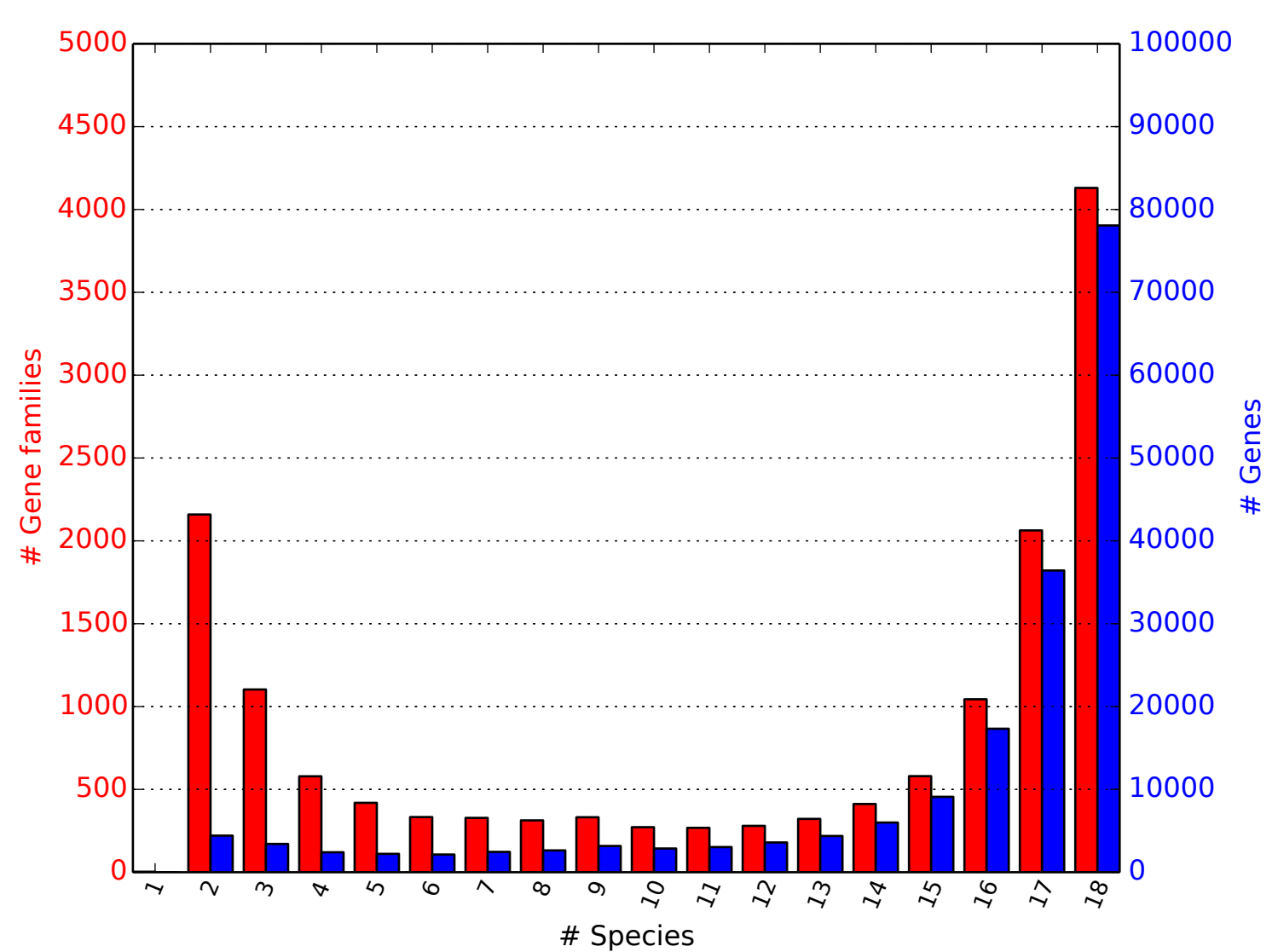

Supplement: Supplementary file 4 — Figure S3. Distribution of gene families number (red bars) and number of genes (blue bars) per families containing x species. Left graph: distribution of the 17,780 raw input gene trees corresponding to 212,800 genes. Middle graph: distribution of the 14,981 gene families, containing 184,719 genes, after discarding families containing included genes (after step 2 of Additional file 3: Figure S2). Right graph: distribution of the 14,940 gene trees, composed of 183,680 genes, after gene trees inference pipeline (after steps 3 and 4 of Additional file 5: Figure S4). (PDF 83 kb) [file 12864_2018_4466_MOESM4_ESM.pdf]

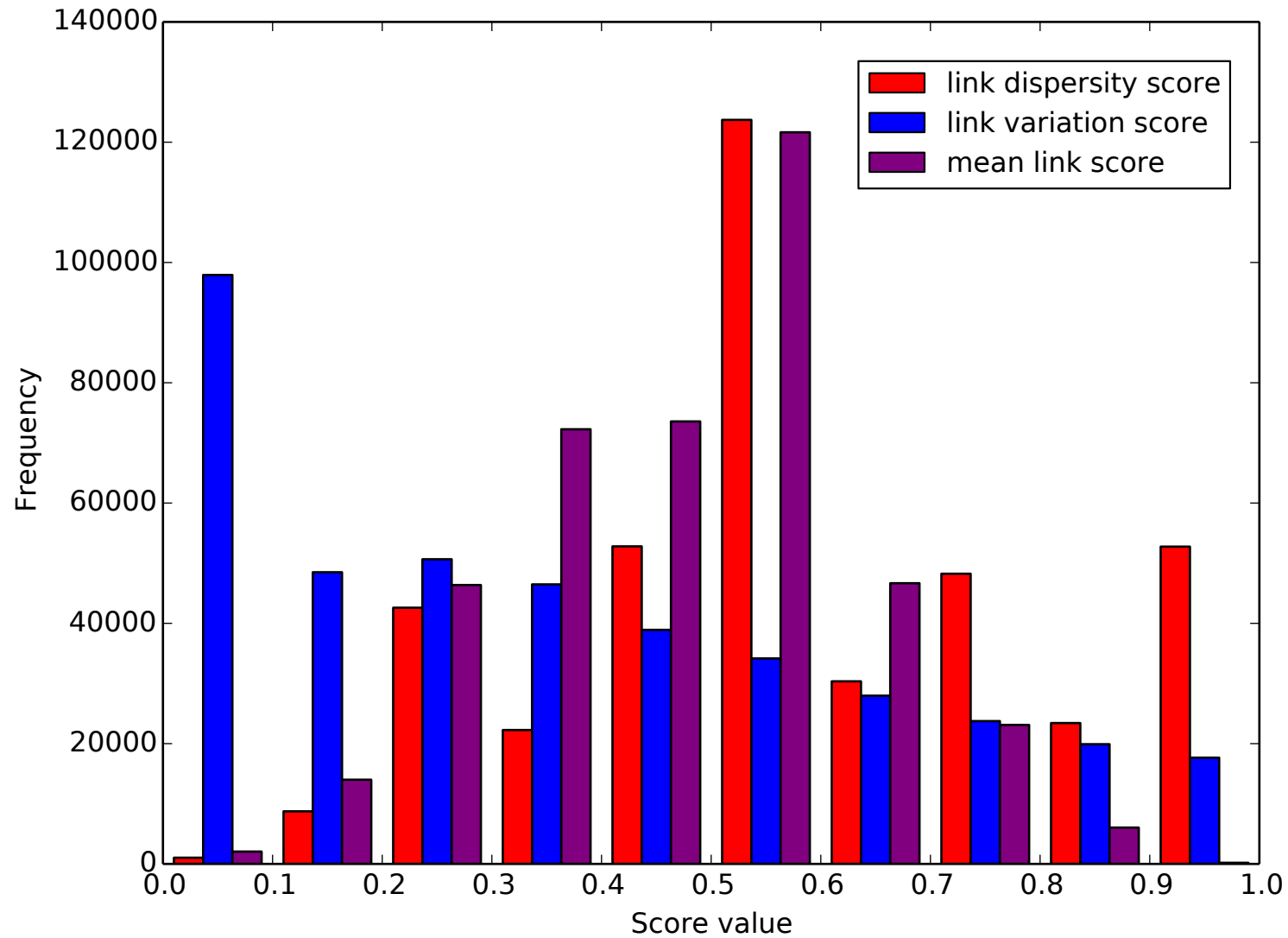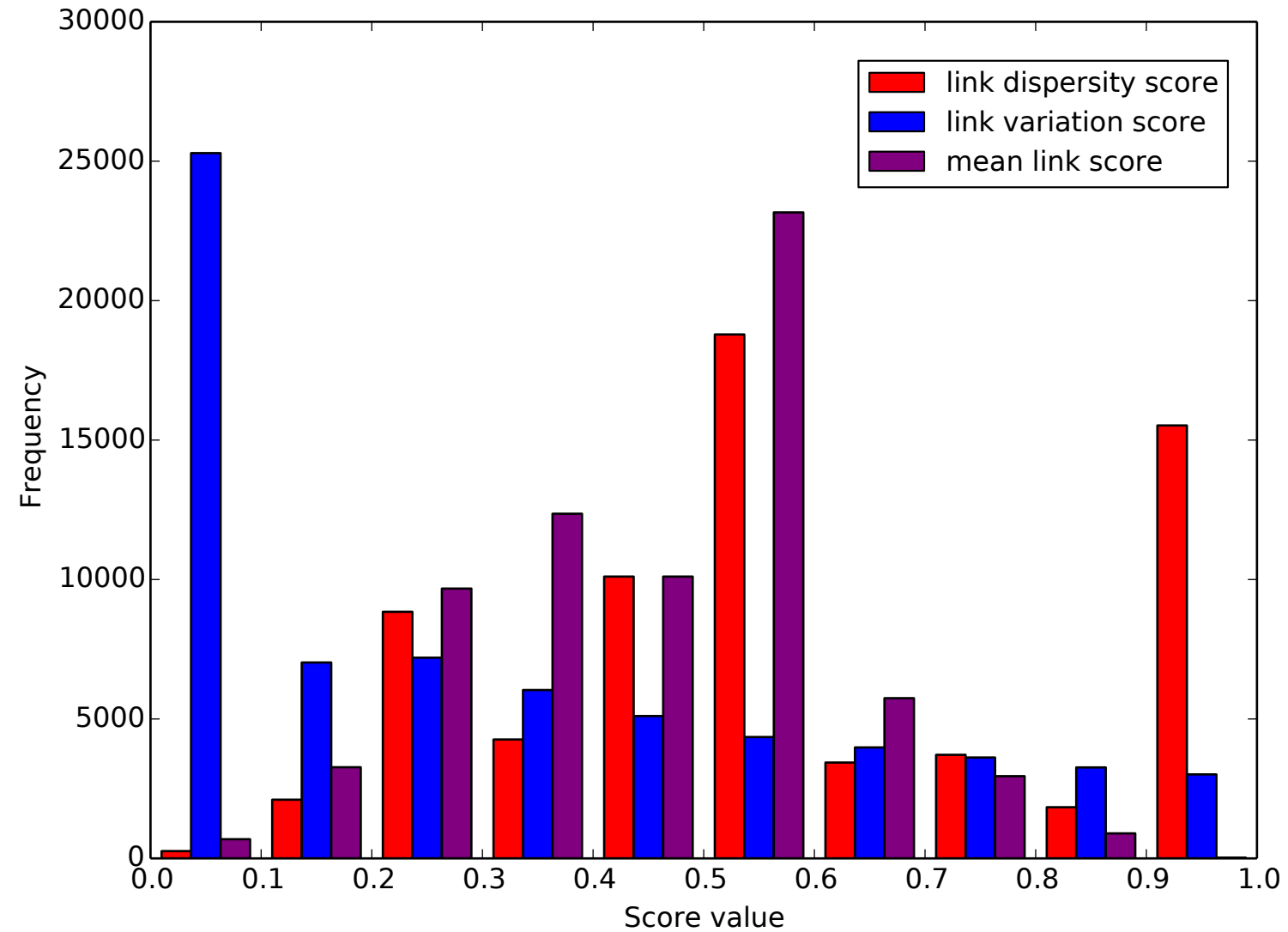

Supplement: Supplementary file 6 — Figure S5. Distributions of scaffolding adjacencies scores computed by BESST for scaffolding adjacencies supported by at least 3 paired reads. Left graph: adjacency scores distribution between all contigs or scaffolds, over 405,939 scaffolding adjacencies. Right graph: adjacency scores distribution for contigs and scaffolds with gene corresponding to the 68,876 scaffolding gene adjacencies considered by DeCoSTAR. Blue bars represent the link variation score, red bars the link dispersity score and purple bars the mean of the two link scores. For more information on the link scores see SI text and [62]. (PDF 37 kb) [file 12864_2018_4466_MOESM6_ESM.pdf]

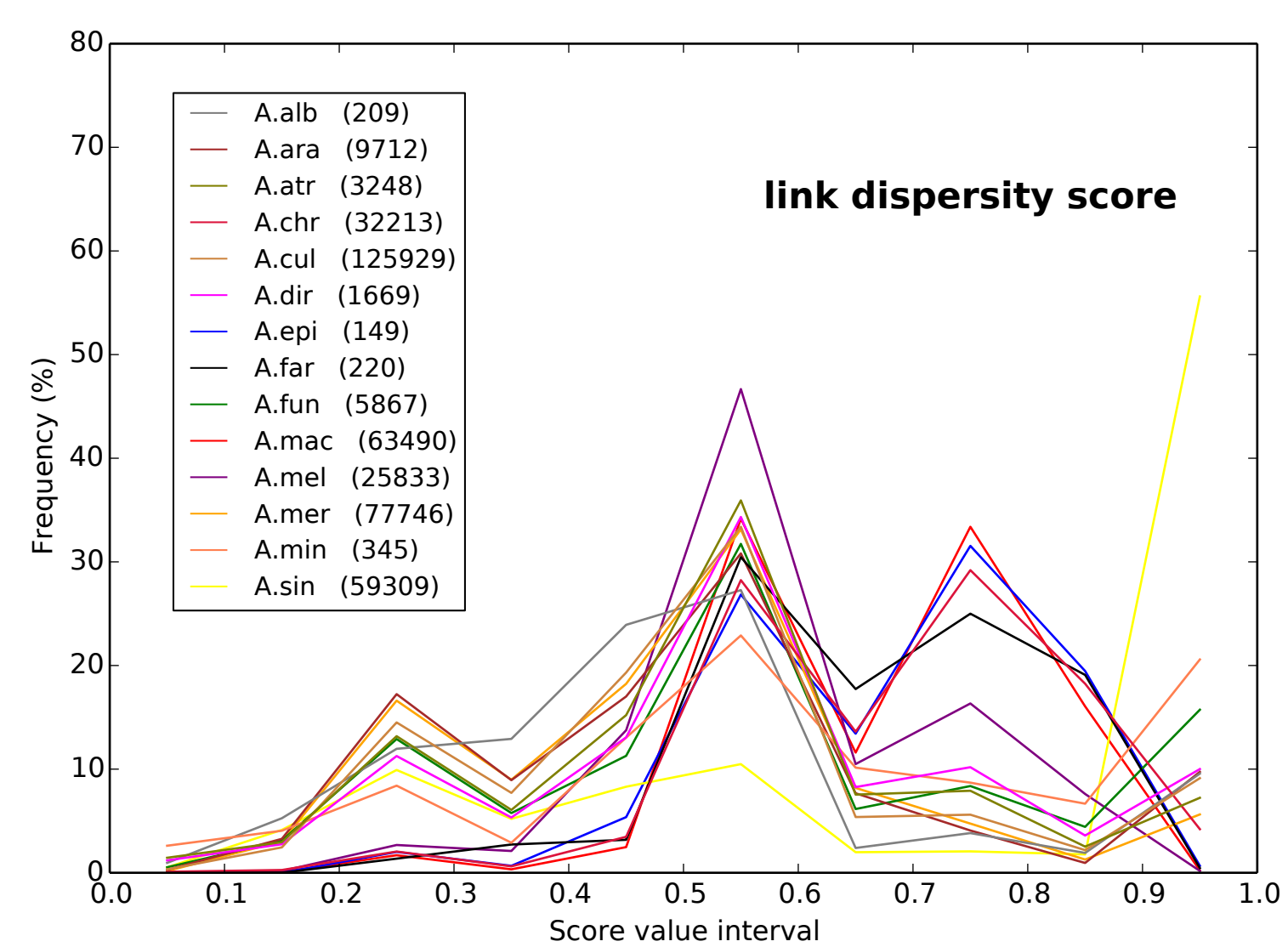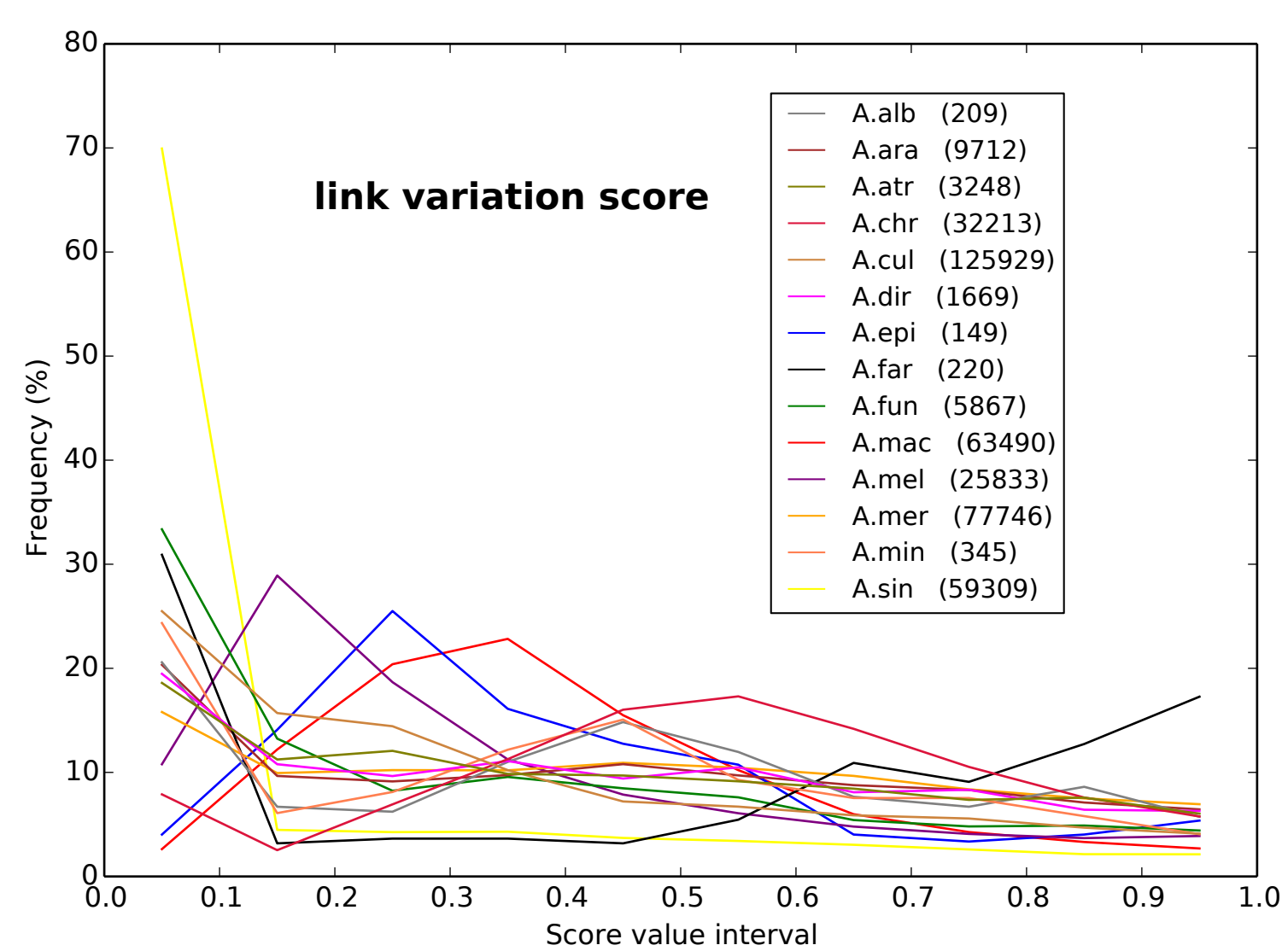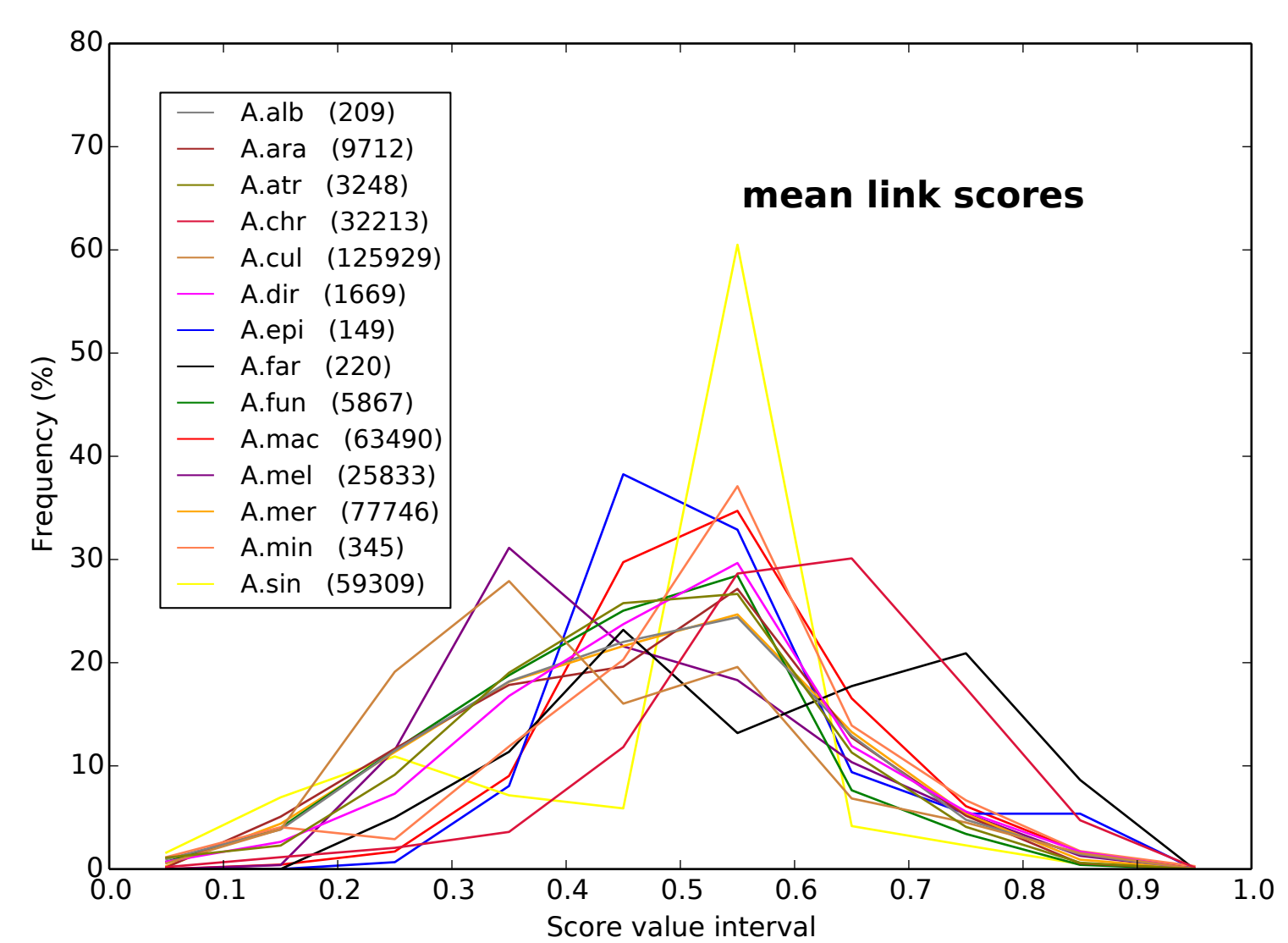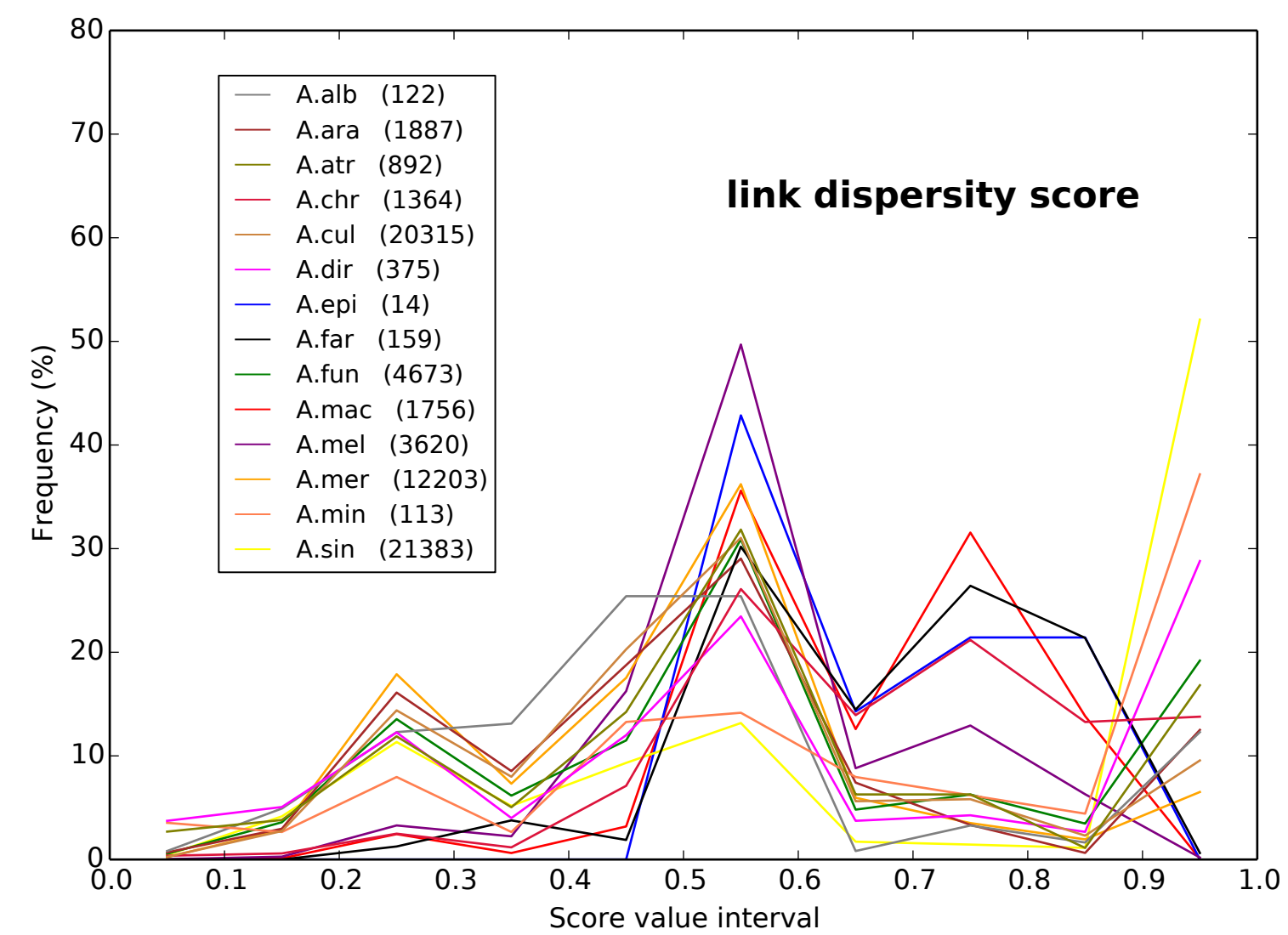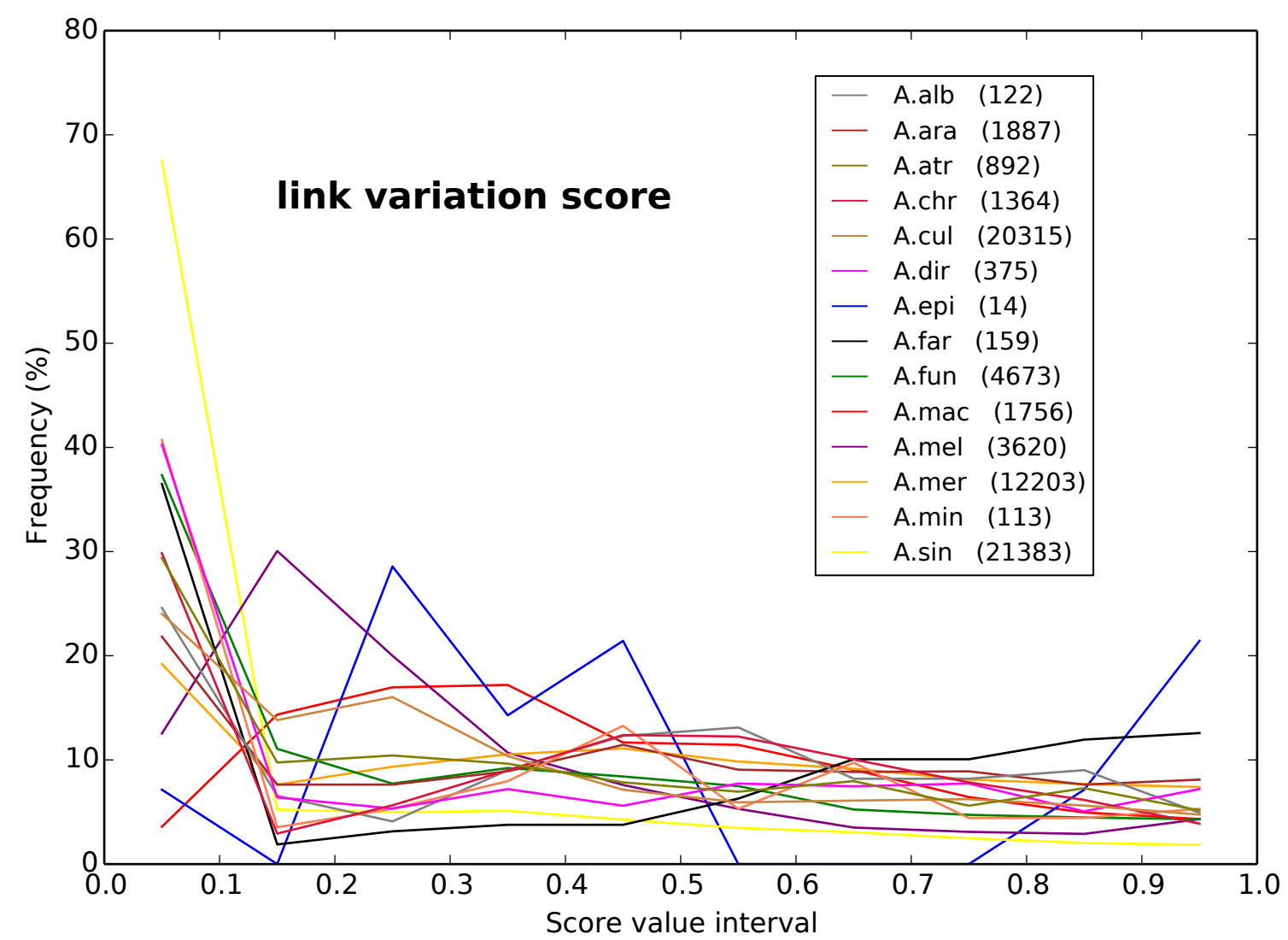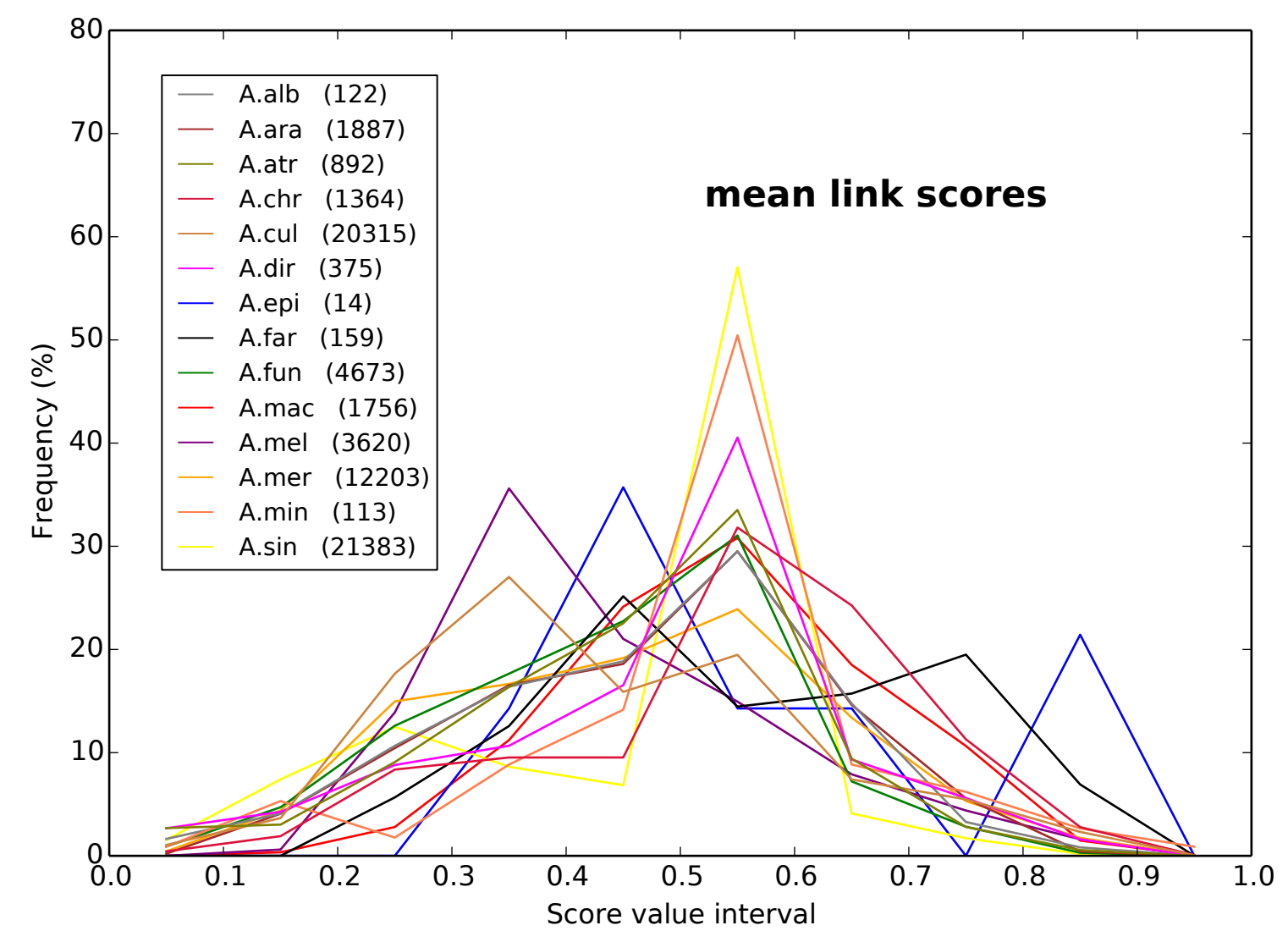

Supplement: Supplementary file 7 — Figure S6. Distributions of scaffolding adjacencies link scores computed by BESST for scaffolding adjacencies supported by at least 3 paired reads, for each of the 18 Anopheles species. Upper graphs: distribution of scores all 405,939 potential scaffolding adjacencies. Lower graphs: distribution of scores for all 68,876 scaffolding gene adjacencies used as input by DeCoSTAR. Left graphs: distribution of link dispersity scores. Middle graphs: distribution of link variation scores. Right graphs: distribution of the mean of link variation and dispersity scores. Each color corresponds to one species and the number between parenthesis in the legend indicates the number of scaffolding adjacencies inferred by BESST for each species. (PDF 64 kb) [file 12864_2018_4466_MOESM7_ESM.pdf]

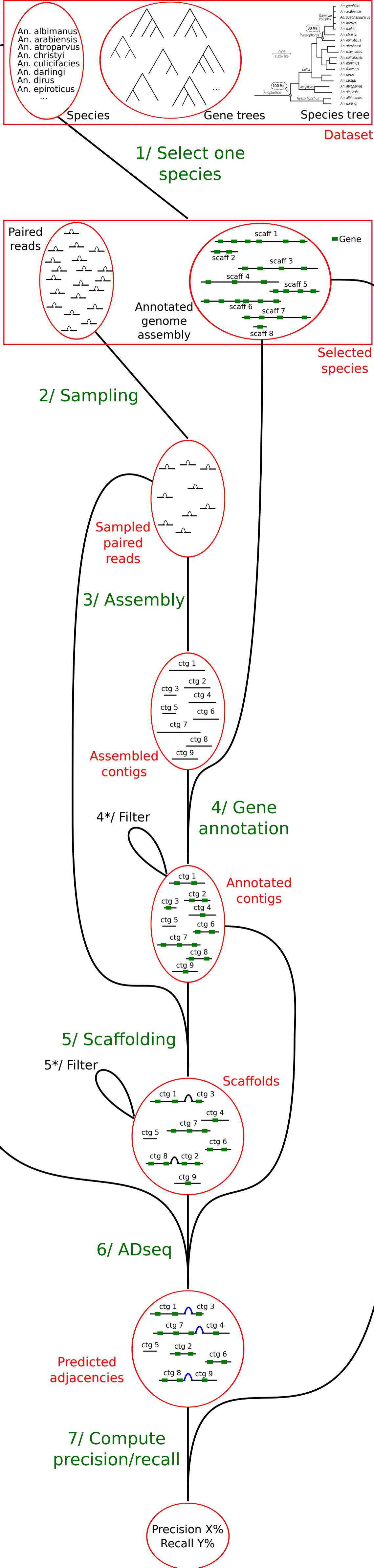

Supplement: Supplementary file 8 — Figure S7. The ADseq validation protocol. (PDF 61 kb) [file 12864_2018_4466_MOESM8_ESM.pdf]

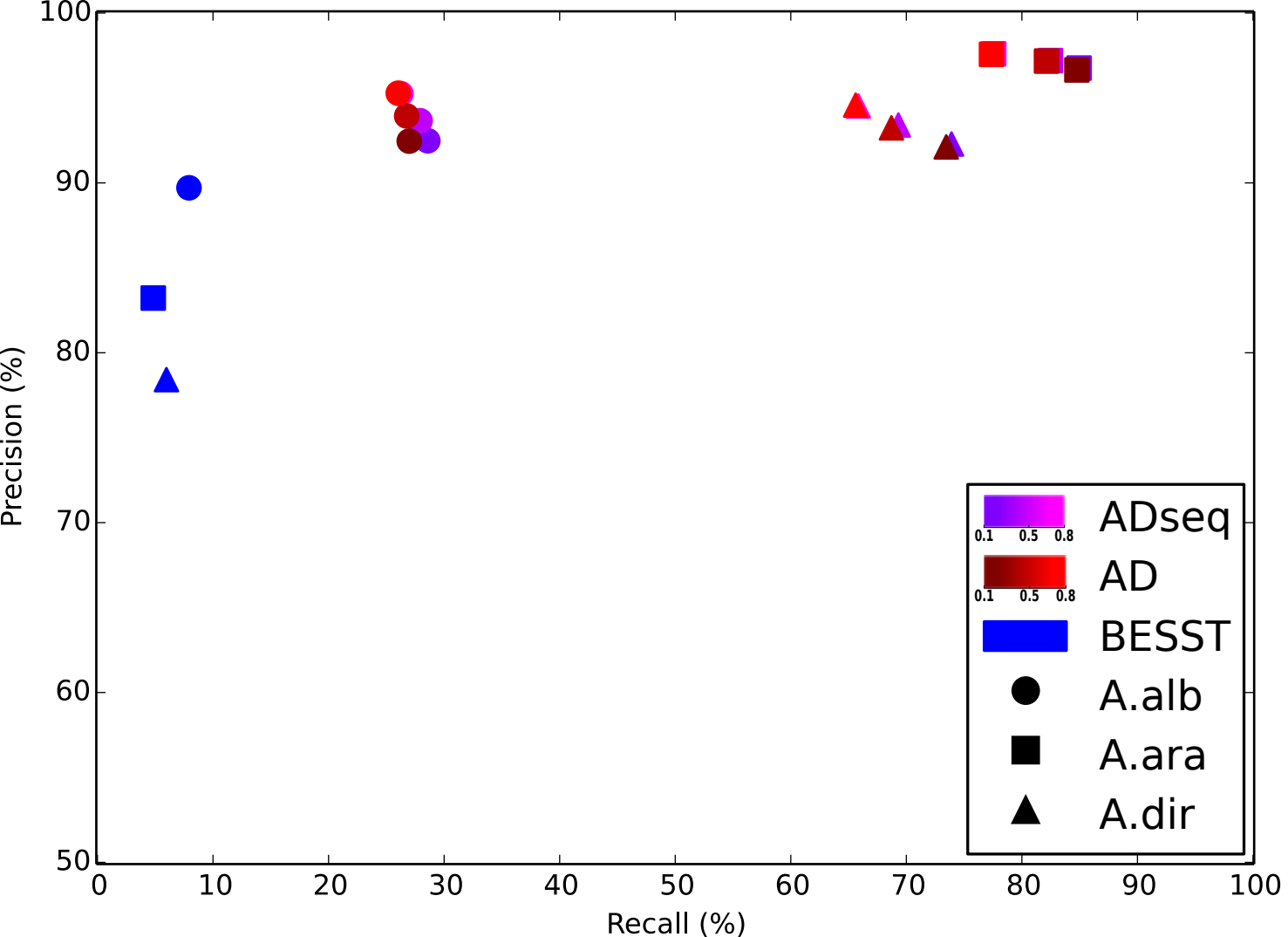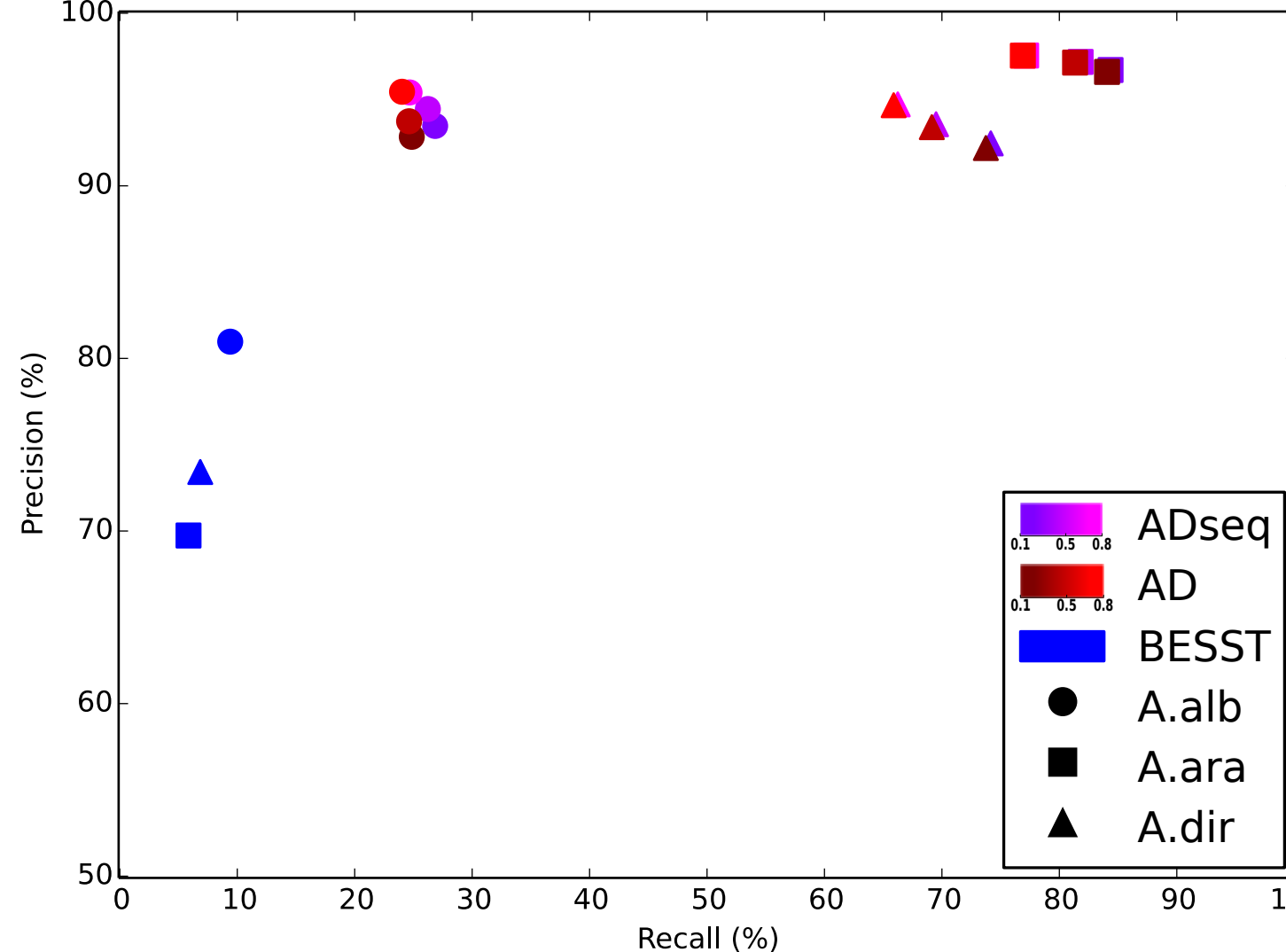

Supplement: Supplementary file 9 — Figure S8. Precision and recall statistics for scaffolding adjacencies on three artificially fragmented genomes (A.alb: Anopheles albimanus, A.ara: Anopheles arabiensis and A.dir: Anopheles dirus), when gene orientations are not accounted for. Left graph: results with 50% of reads. Right graph: results with all reads. The different methods results are plotted with the precision on the Y axis and the recall on the X axis. For ADseq and AD, results for three different adjacency support threshold (0.1, 0.5 and 0.8) before genome linearization are plotted and represented with a color gradient. These results show similar results to Fig. 2 showing that for most of the predicted adjacencies the three methods infer the correct gene orientation. (PDF 106 kb) [file 12864_2018_4466_MOESM9_ESM.pdf]

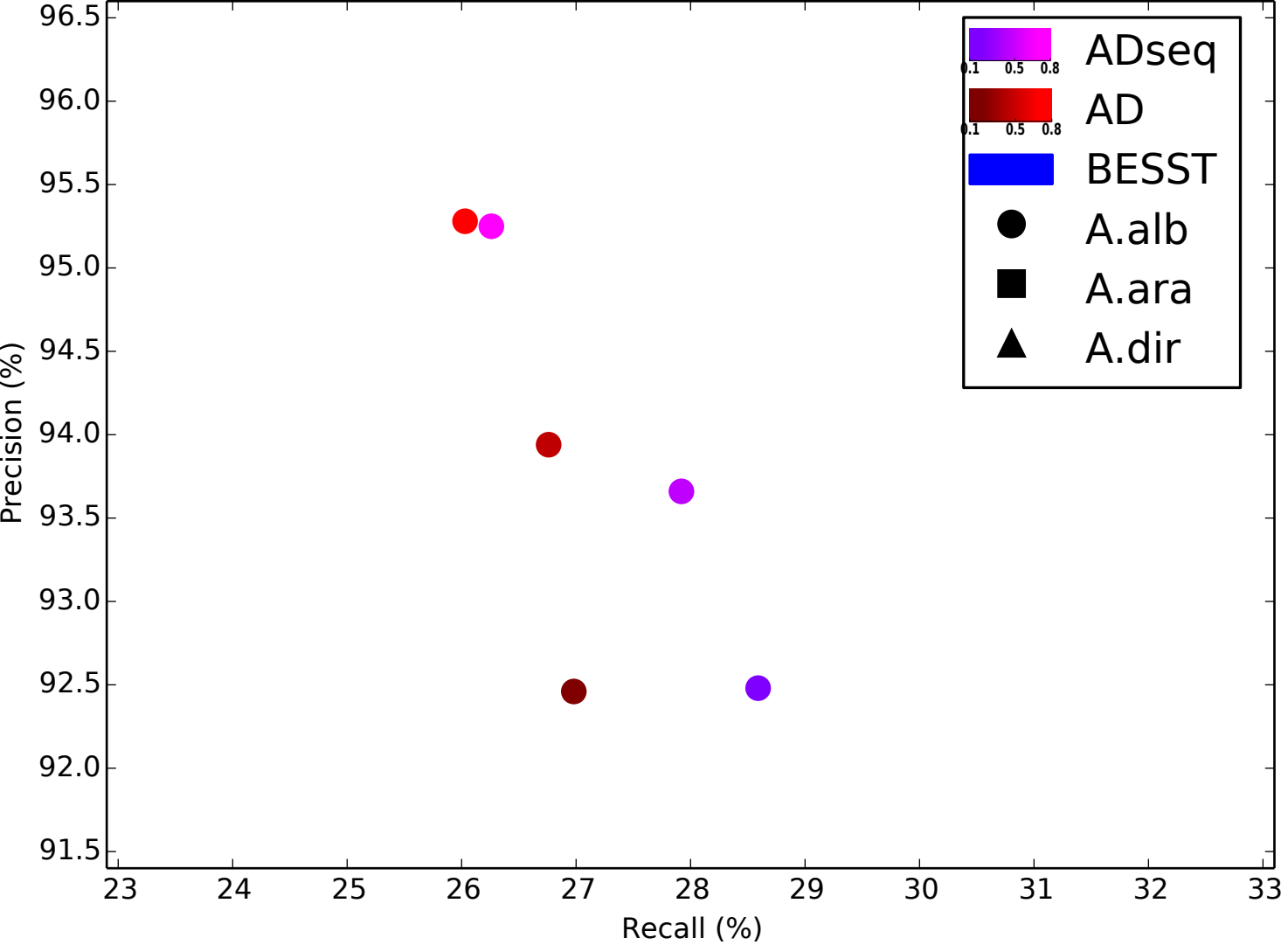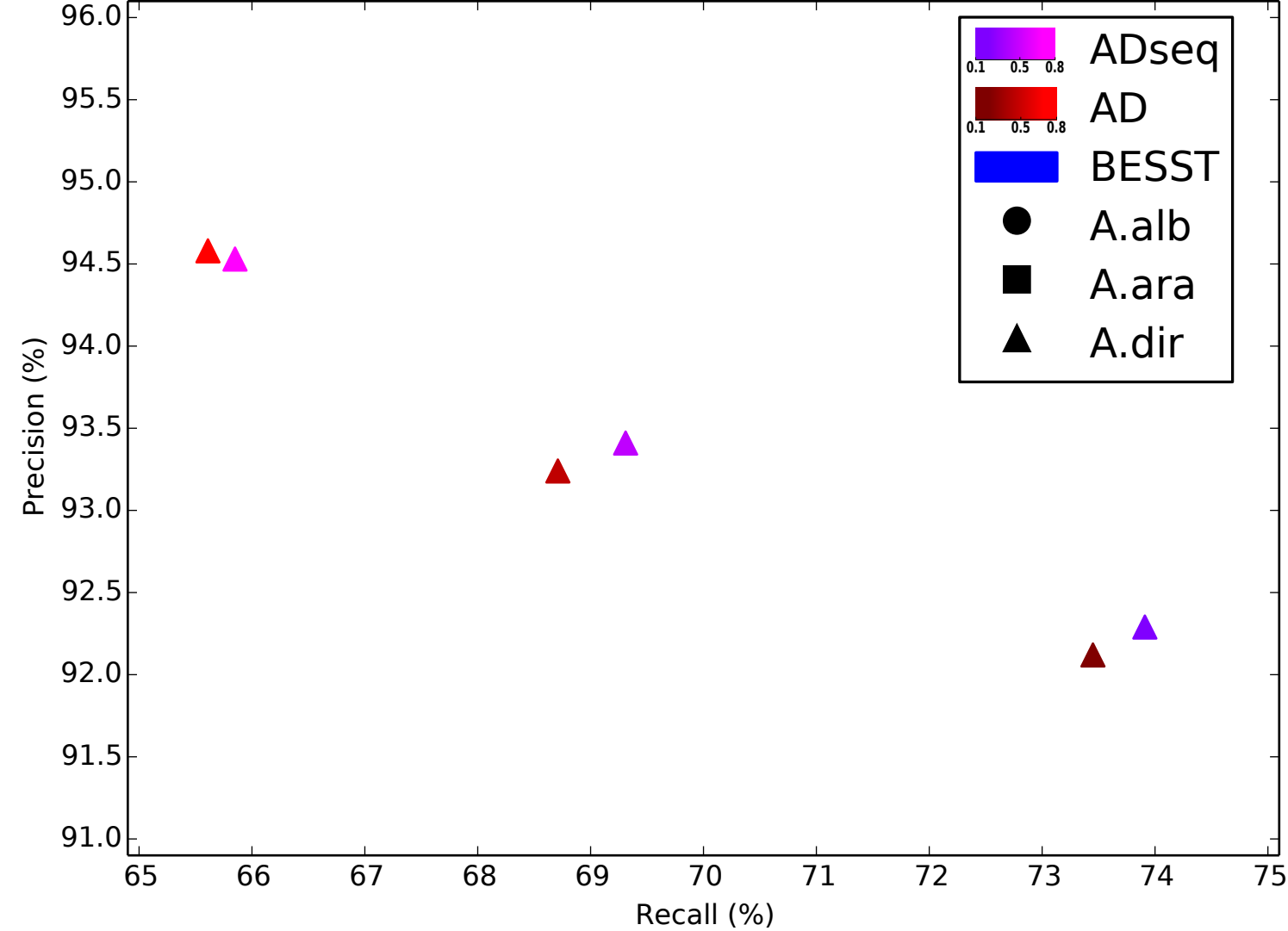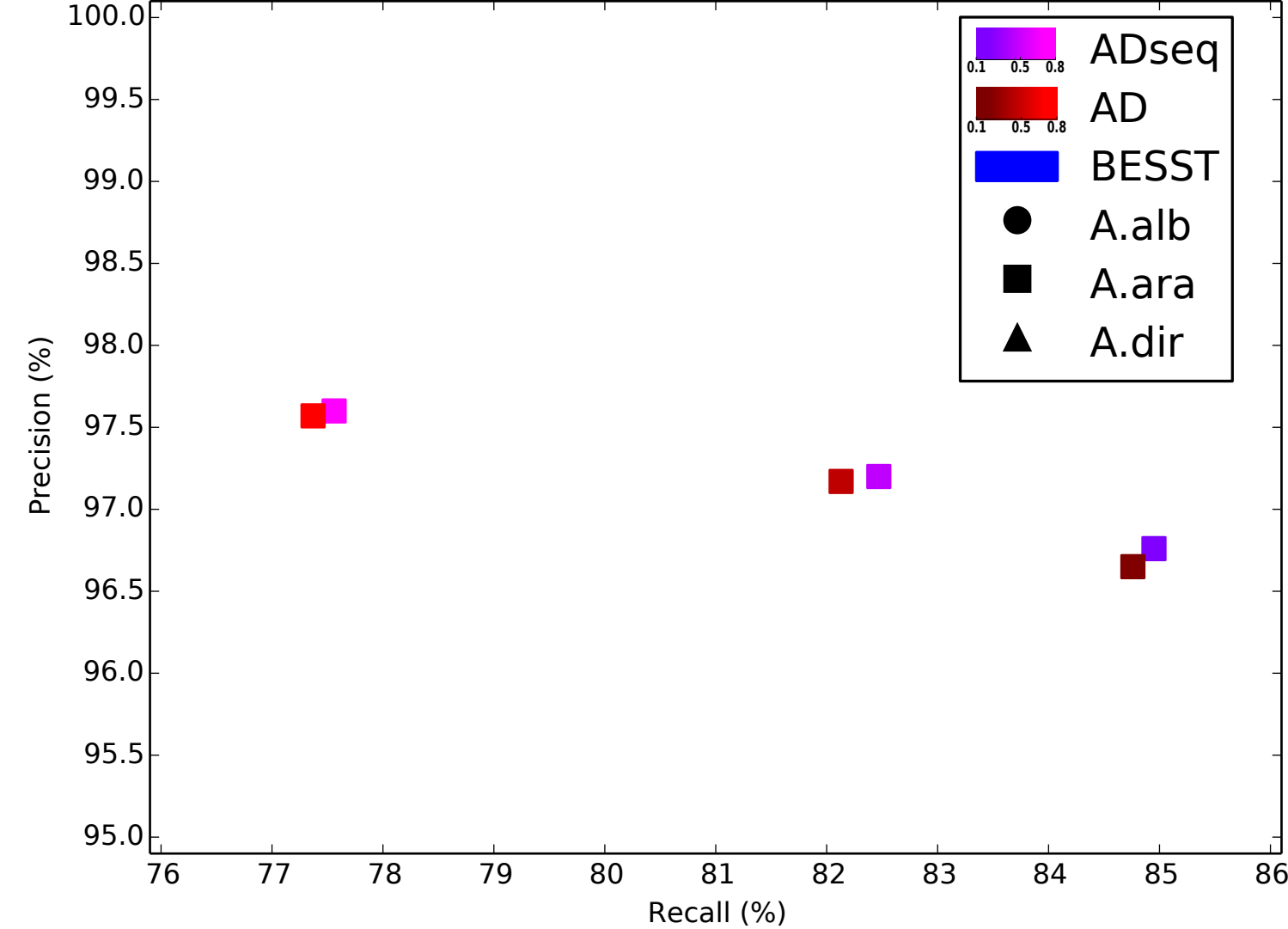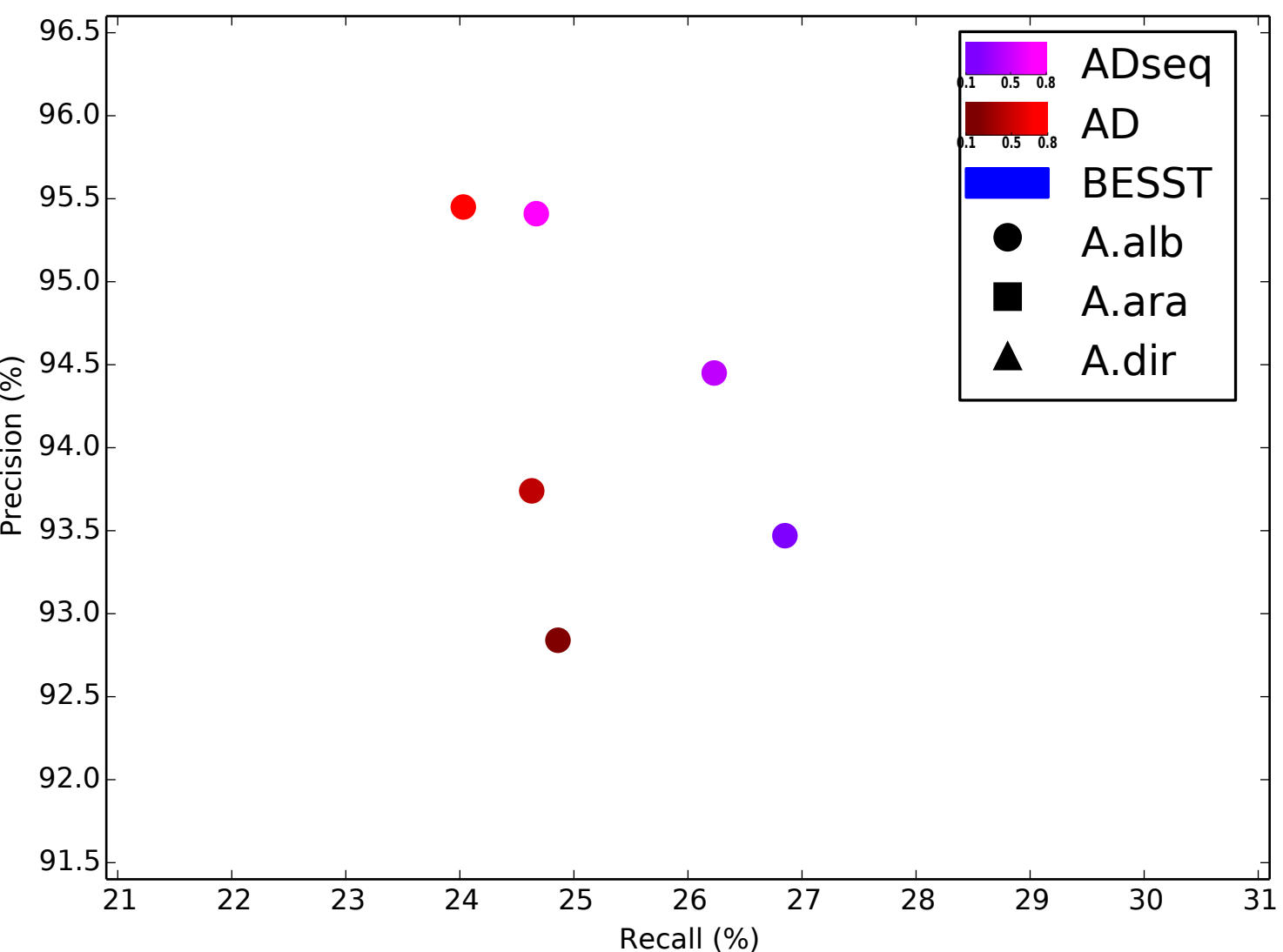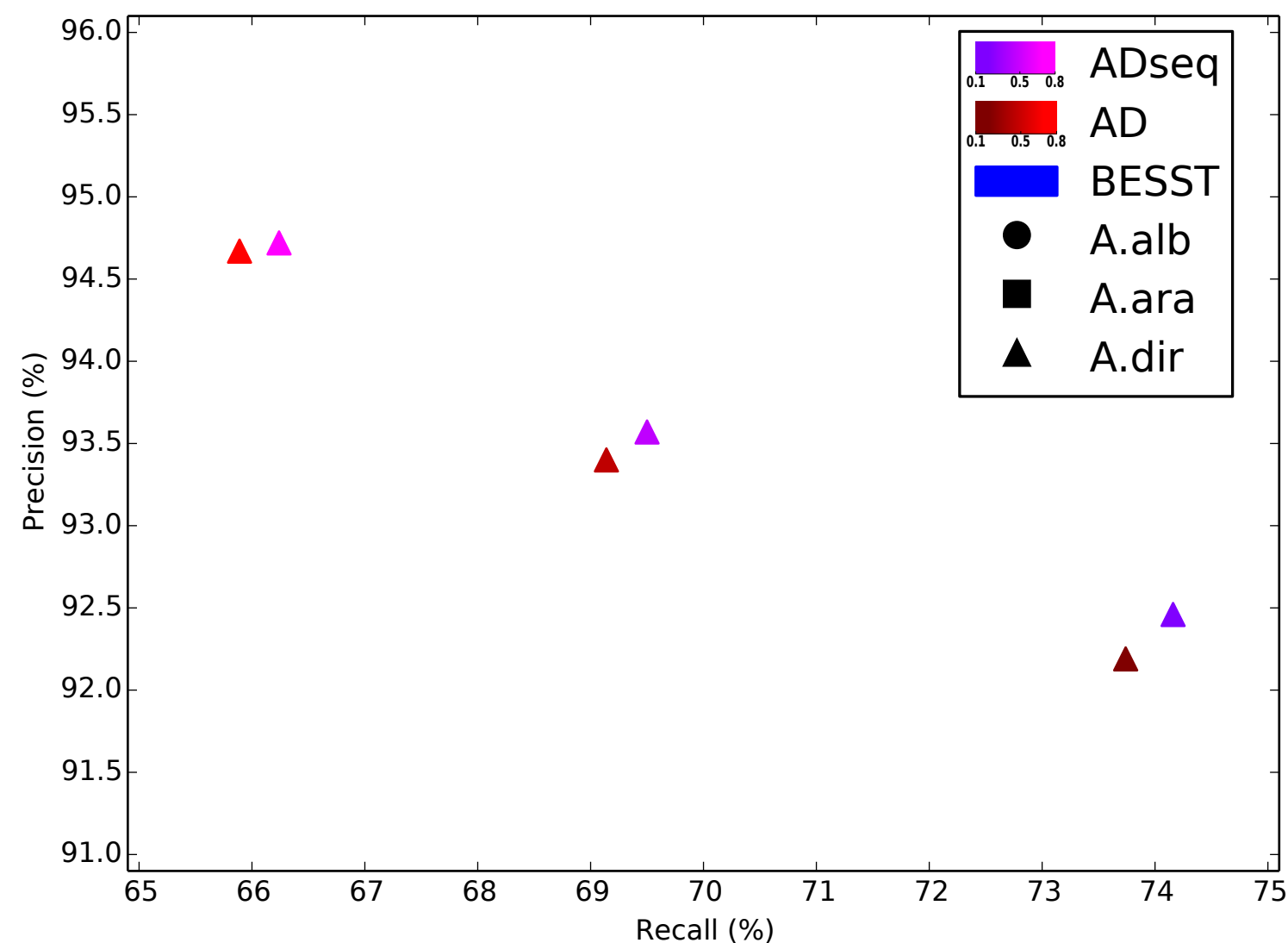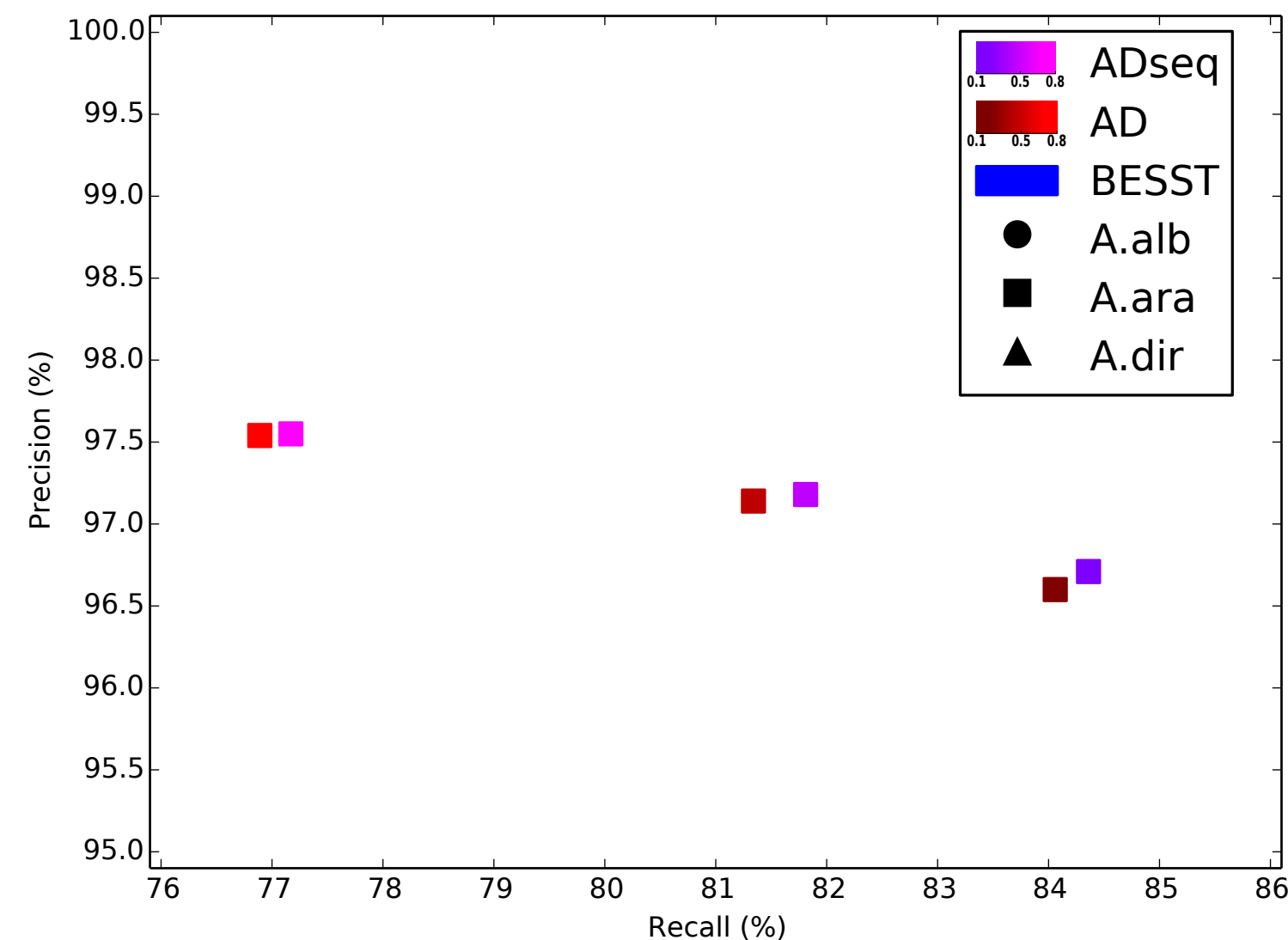

Supplement: Supplementary file 10 — Figure S9. Subfigures zooming of Additional file 9: Figure S8 to compare precision and recall statistics between ADseq and AD. Upper graphs: zoom of results with 50% of reads. Lower graphs: results with all reads. (PDF 154 kb) [file 12864_2018_4466_MOESM10_ESM.pdf]

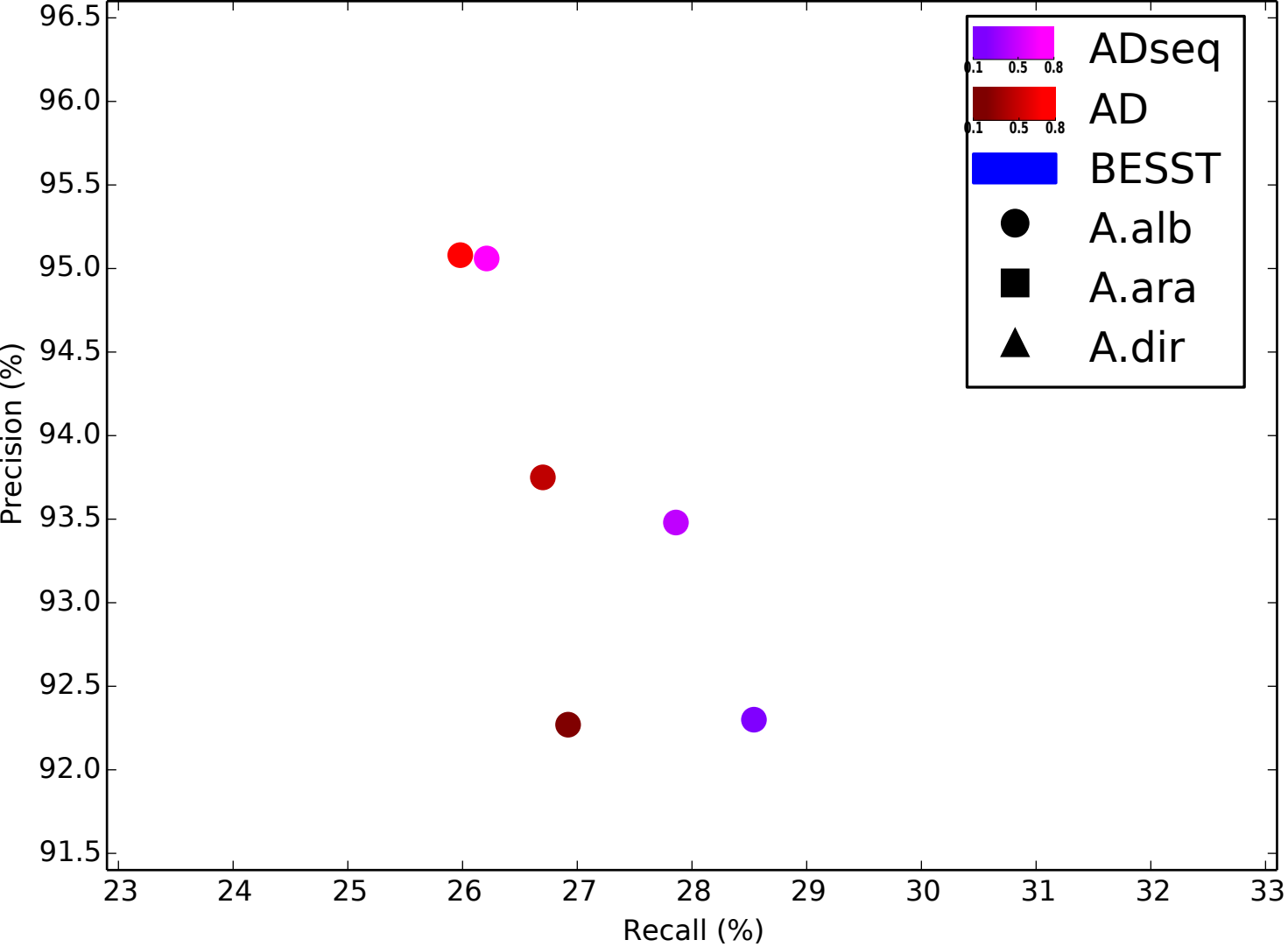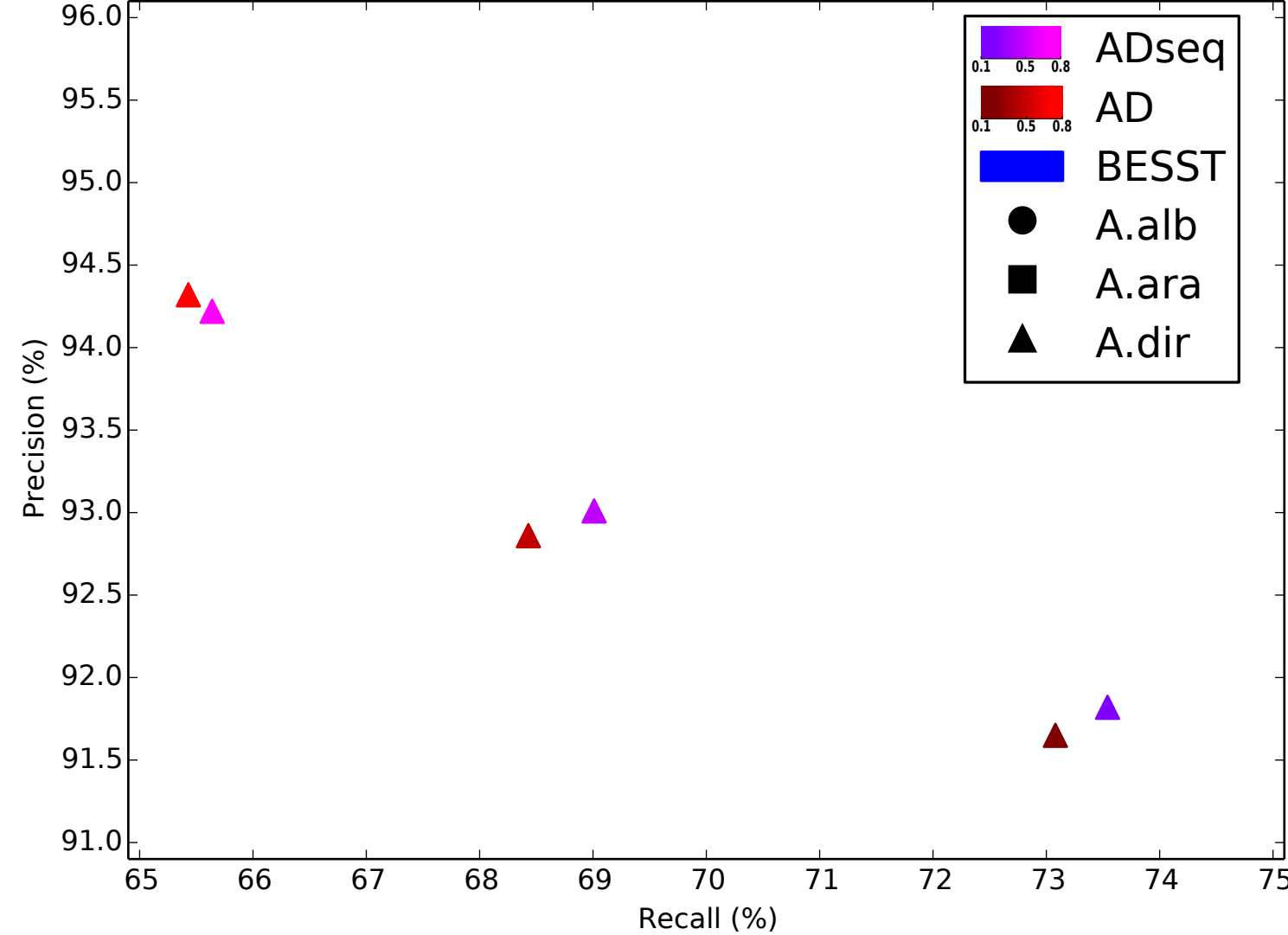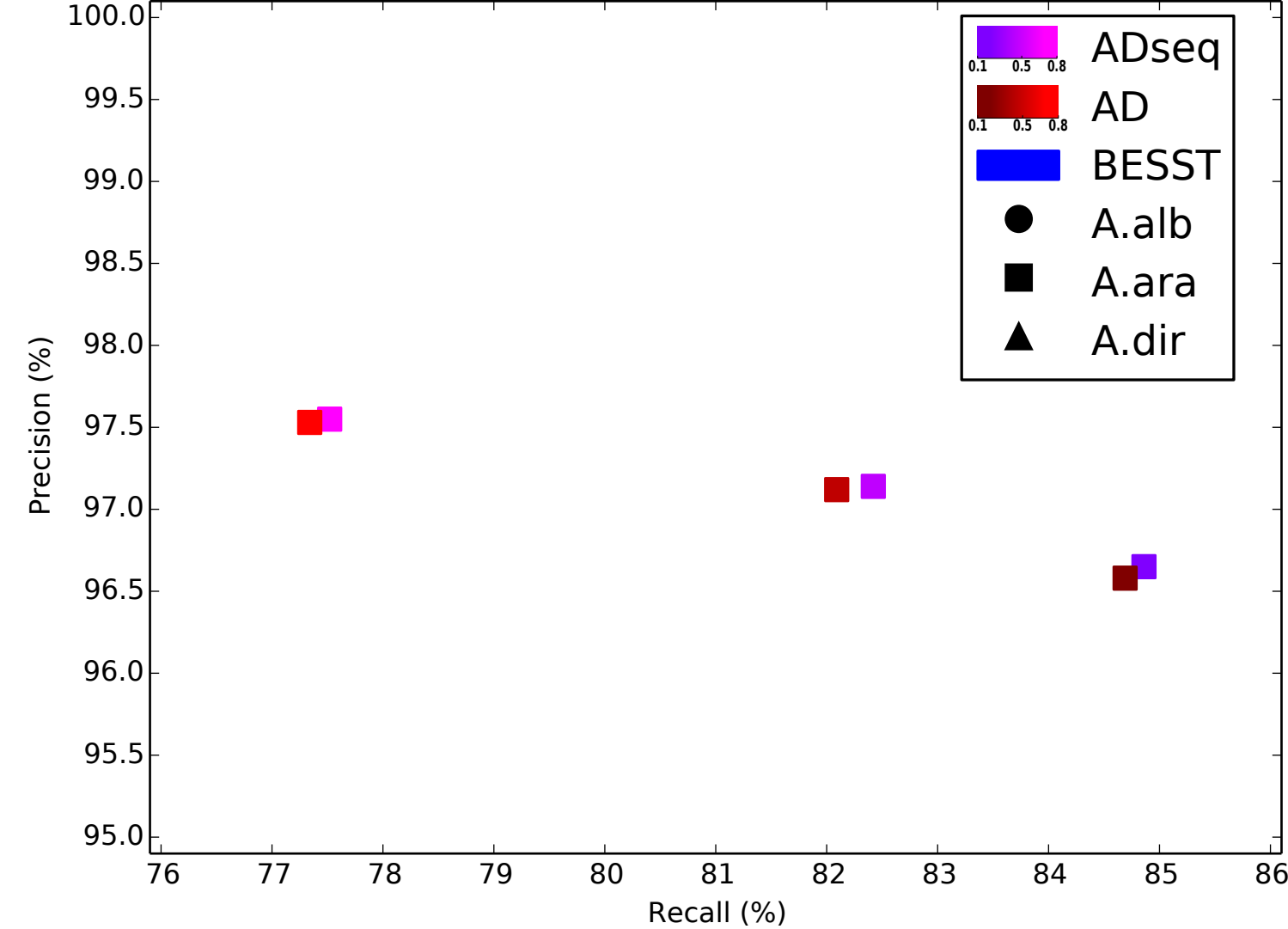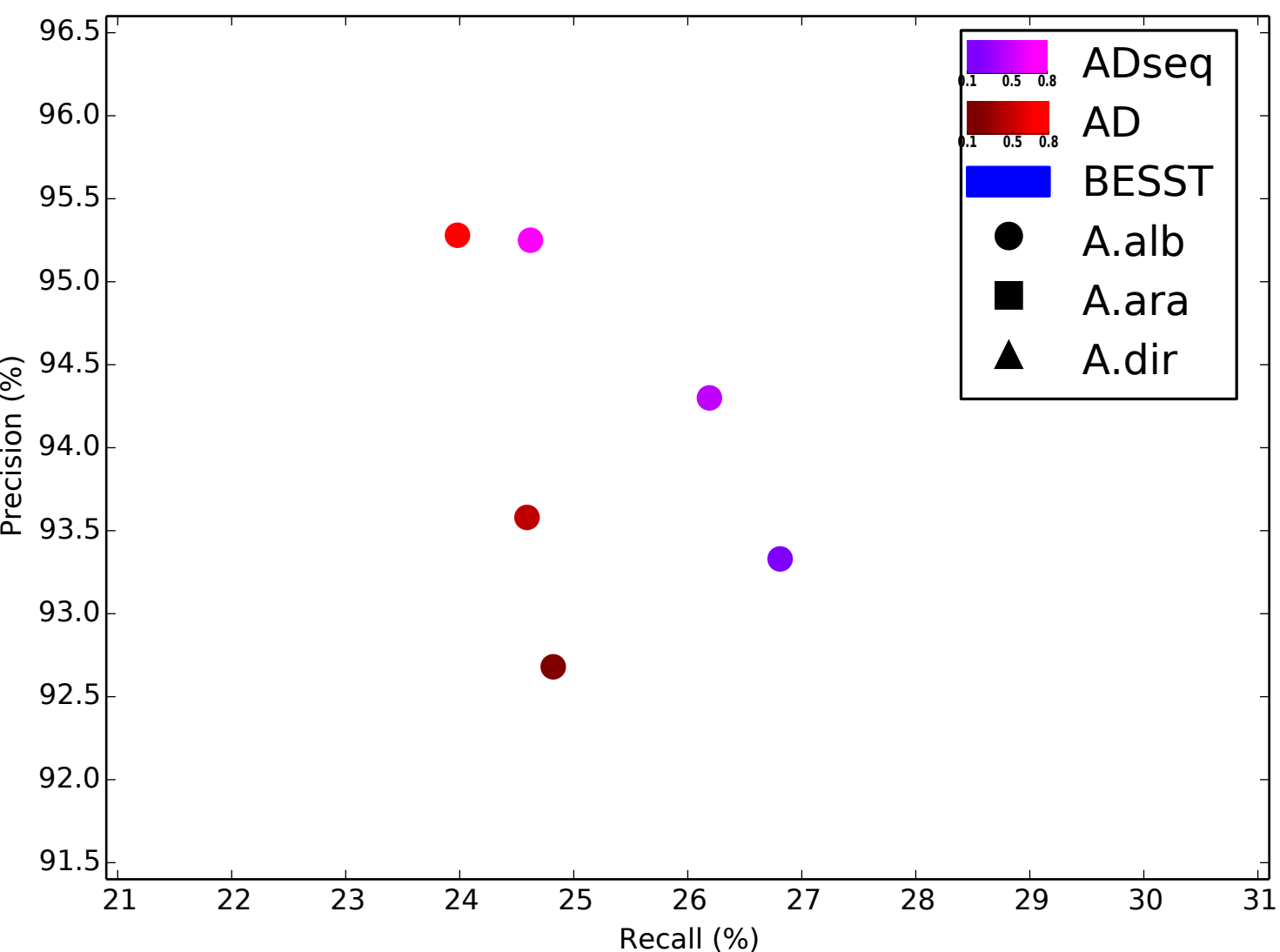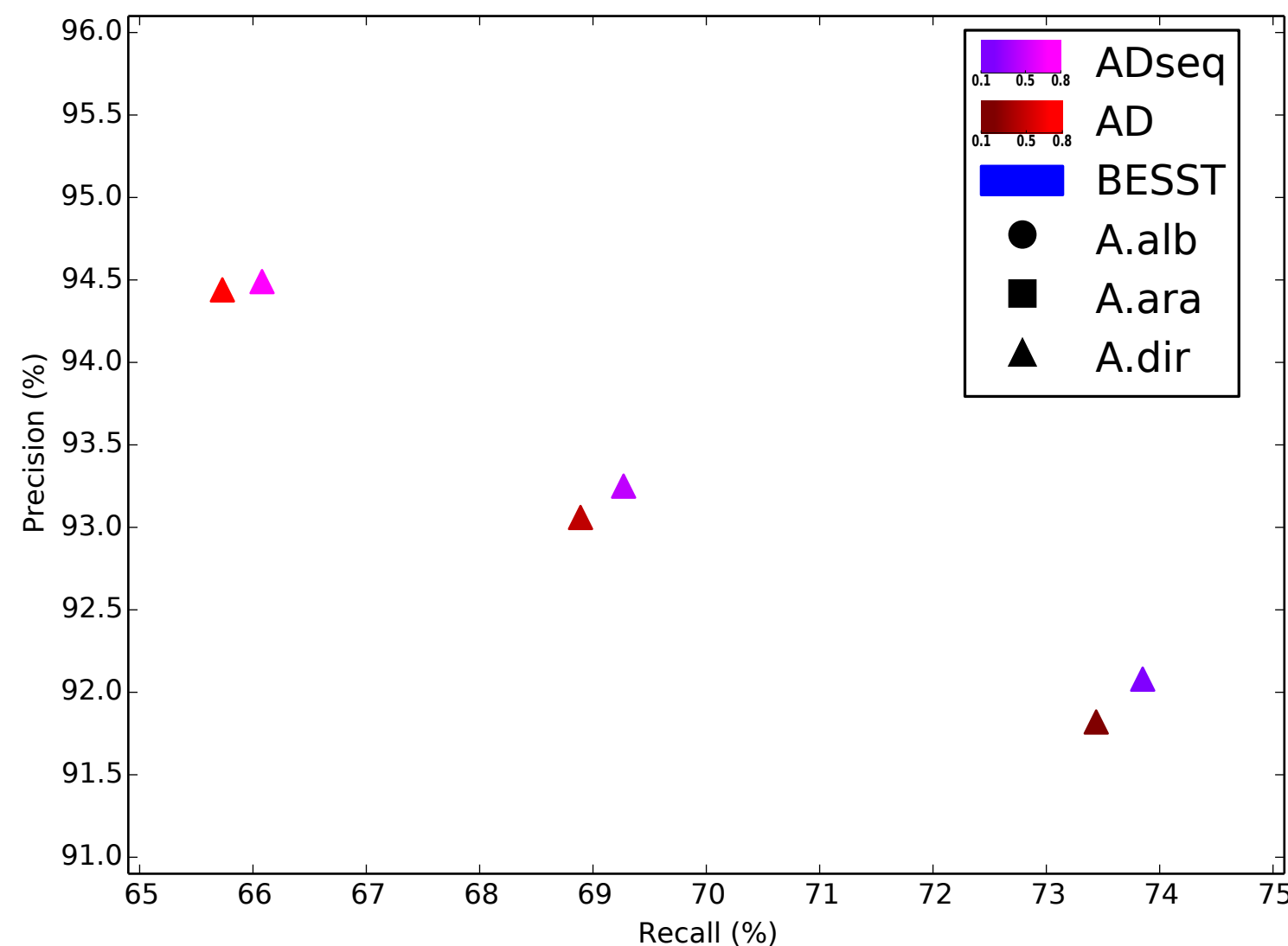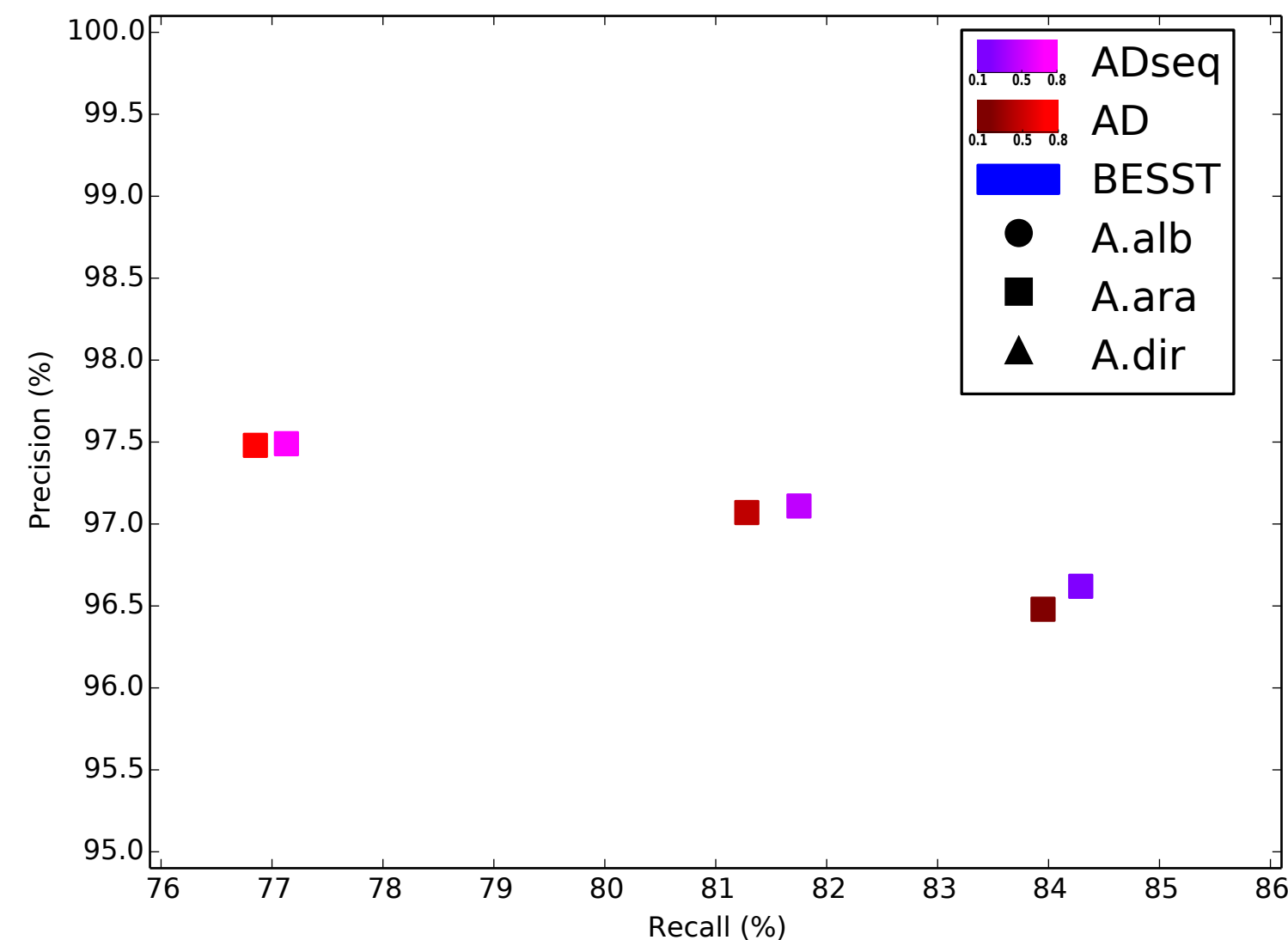

Supplement: Supplementary file 11 — Figure S10. Subfigures zooming of Additional file 3: Figure S2 to compare precision and recall statistics between ADseq and AD. Upper graphs: zoom of results with 50% of reads. Lower graphs: results with all reads. (PDF 136 kb) [file 12864_2018_4466_MOESM11_ESM.pdf]

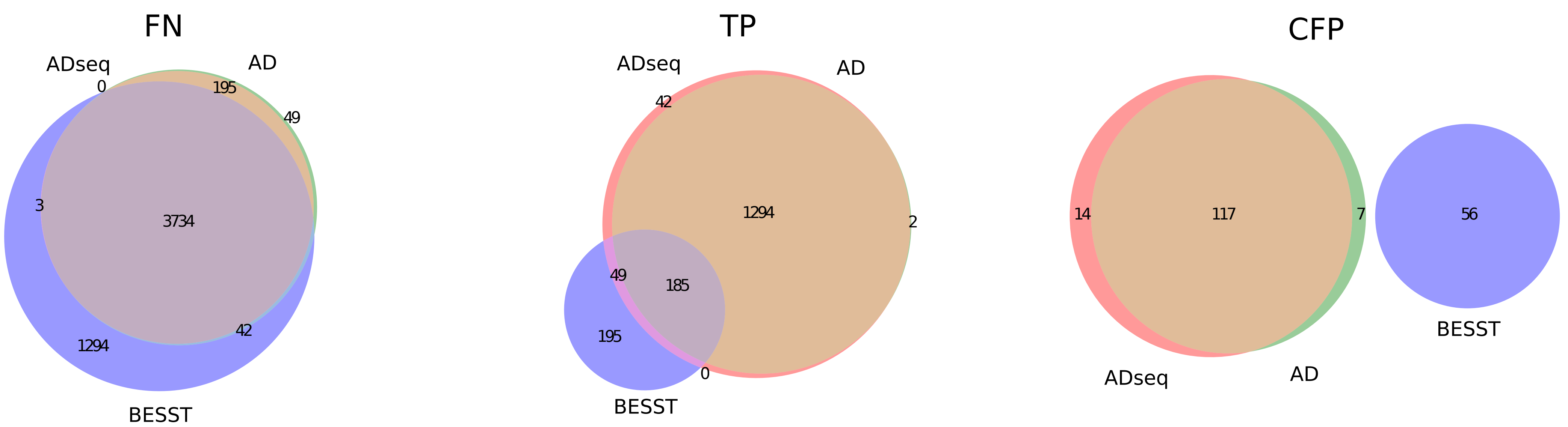

# Anopheles albimanus

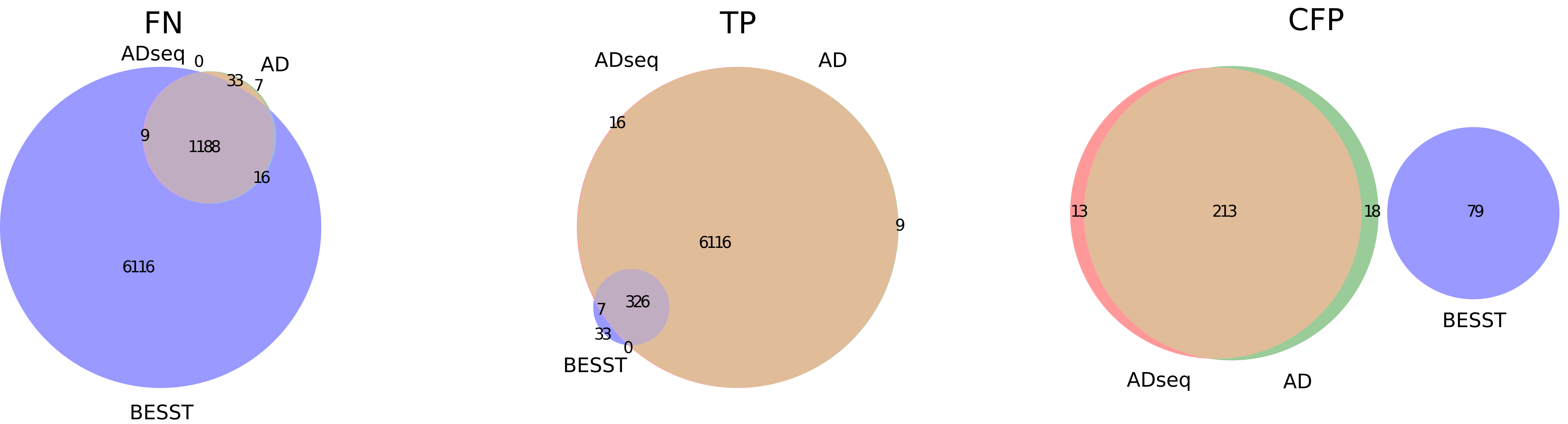

# Anopheles arabiensis

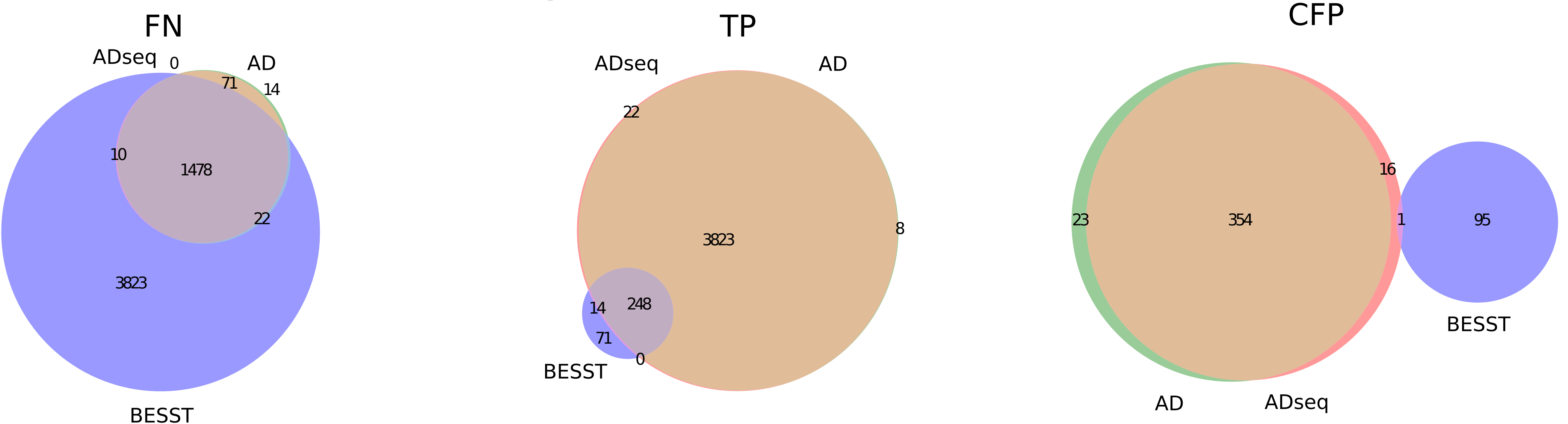

# Anopheles dirus

Supplement: Supplementary file 12 — Figure S11. Venn diagrams showing adjacencies shared by the three scaffolding methods ADseq, AD and BESST with a sample of 50% of the reads. Upper Venn diagrams: results for Anopheles albimanus. Middle Venn diagrams: results for Anopheles arabiensis. Lower Venn diagrams: results for Anopheles dirus. Left diagrams: False Negative (FN) adjacencies, corresponding to adjacencies created by the fragmentation process and that have not been recovered. Center diagrams: results for True Positive (TP) adjacencies. Here, an adjacency is considered TP if the pair of genes is adjacent in the reference assembly and the orientation of genes involved in the adjacency is properly recovered. Right diagrams: results for Certain False Positive (CFP) adjacencies. An adjacency is determined as CFP when the pair of gene does not belong to the reference assemblies and one of the two genes is not located at a contig extremity in reference genome, or if the recovered orientation of genes is incorrect. If we consider method individually, these results show that ADseq outperforms AD and BESST with the lowest number of FN adjacencies, the largest number of TP adjacencies and the lowest number of CFP adjacencies (except for An. albimanus where AD has the lowest number of CFP (224 vs. 231 for ADseq). However, if we combine a posterioriAD and BESST, this performs better than ADseq in terms of recall (higher number of TP adjacencies) but at the expense of a strong decreases of precision (much higher number of CFP adjacencies). (PDF 20 kb) [file 12864_2018_4466_MOESM12_ESM.pdf]

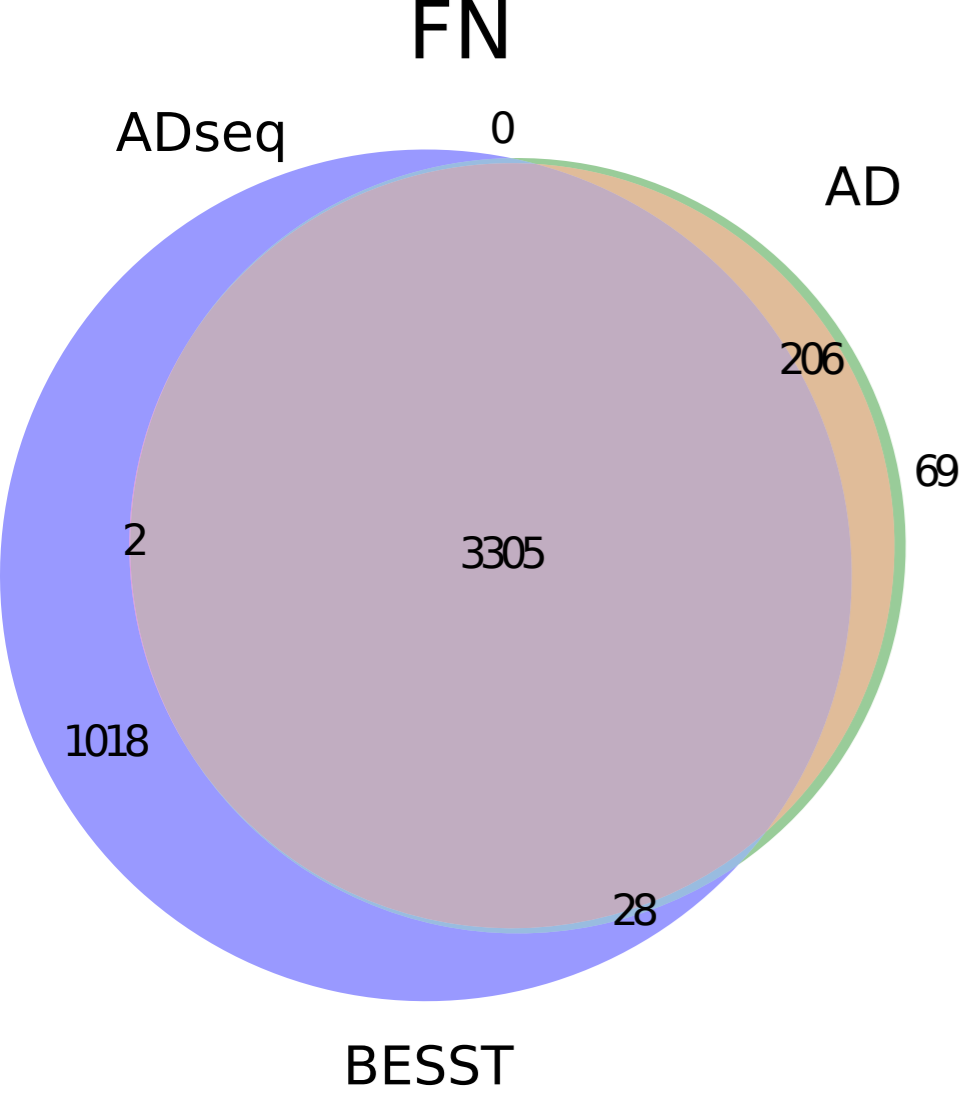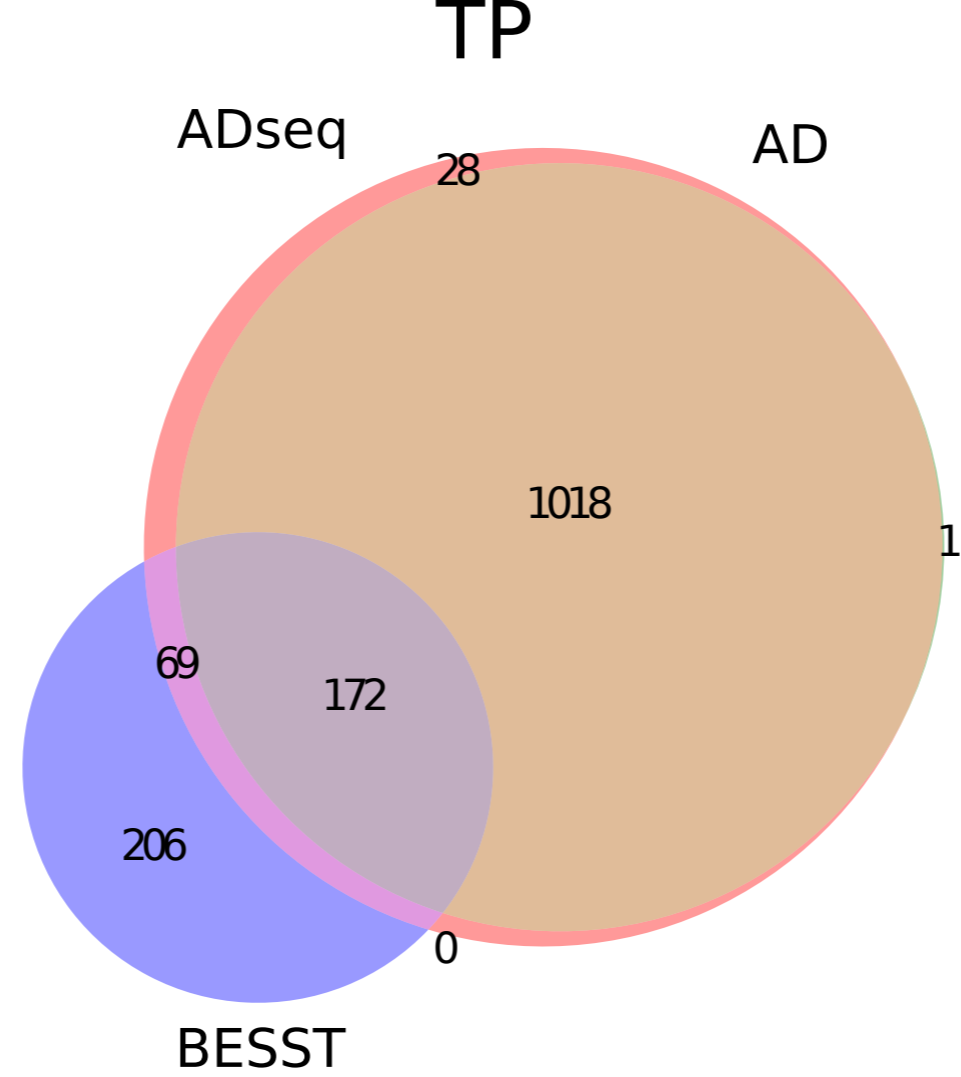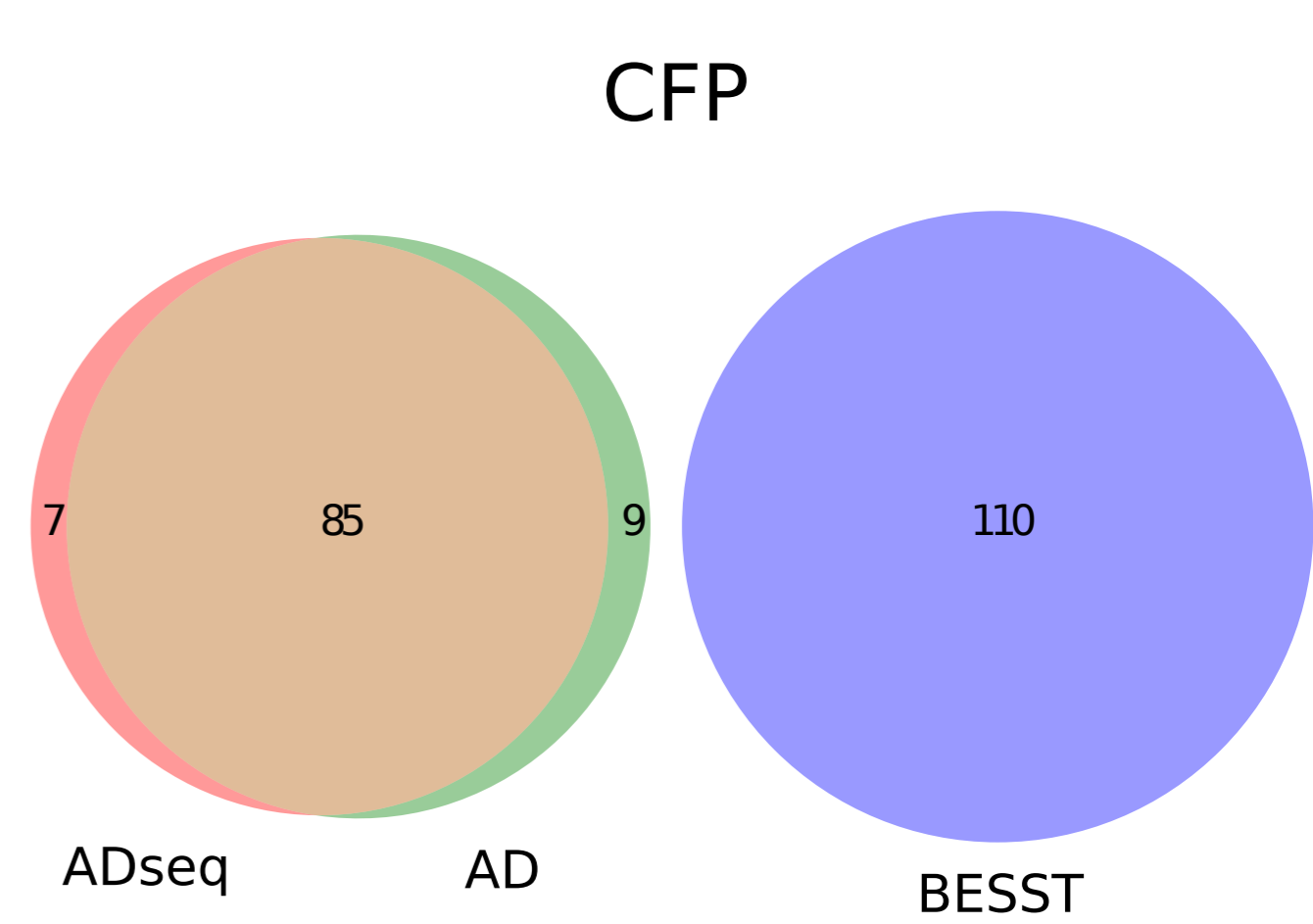

## Anopheles albimanus

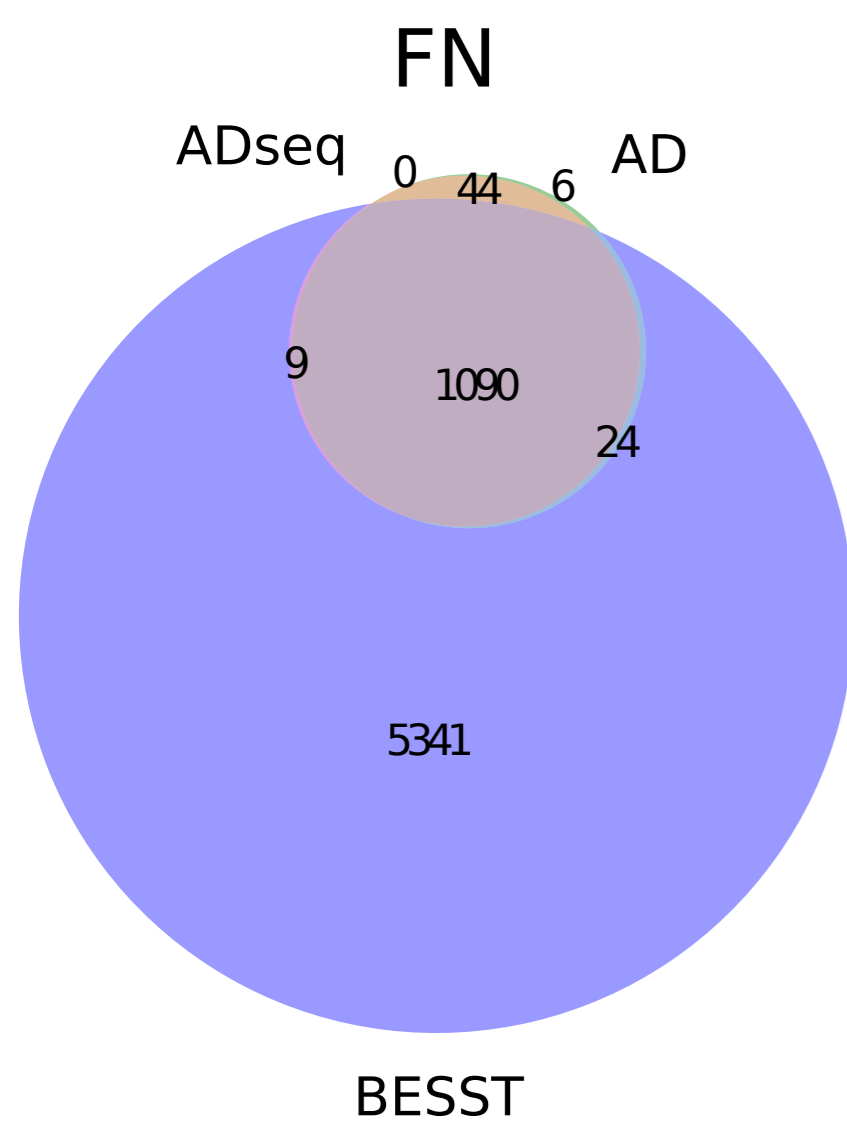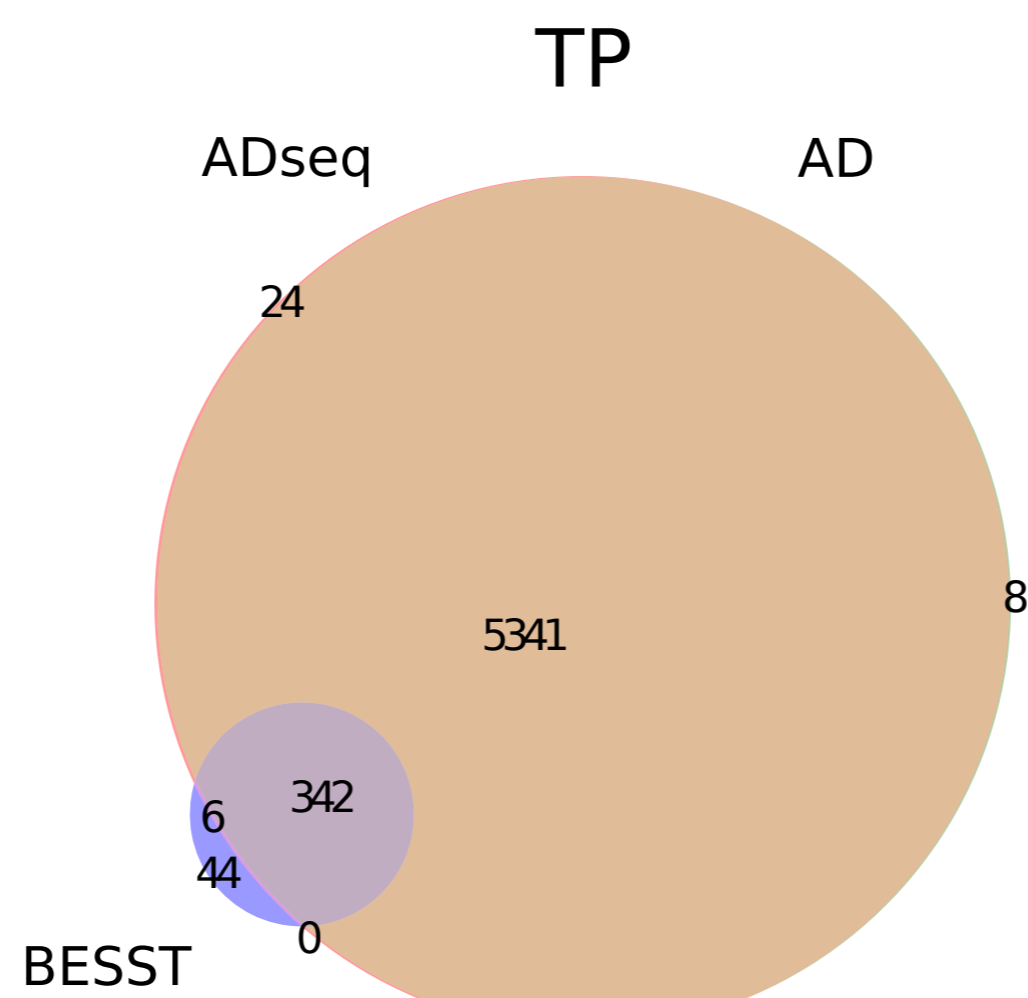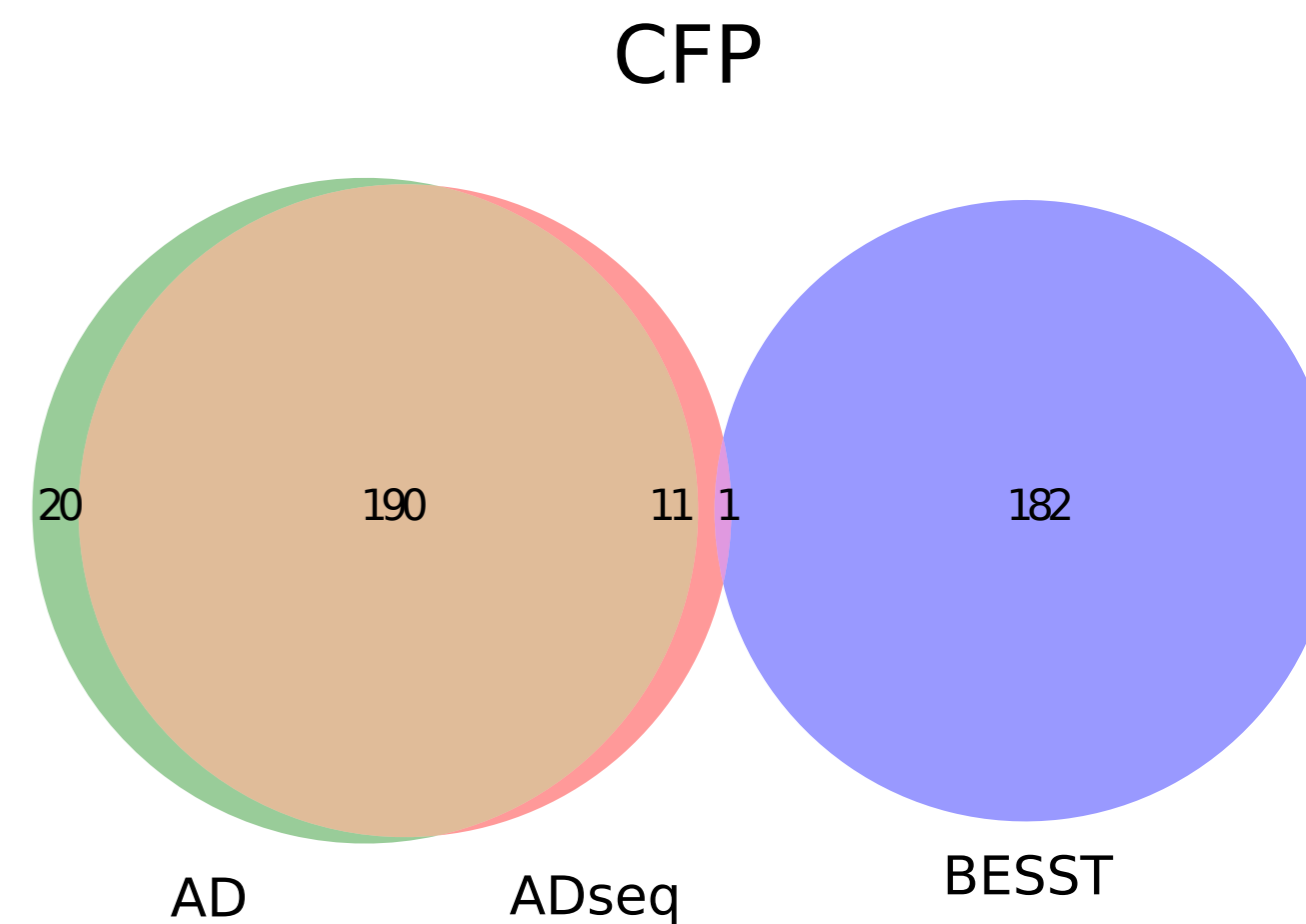

## Anopheles arabiensis

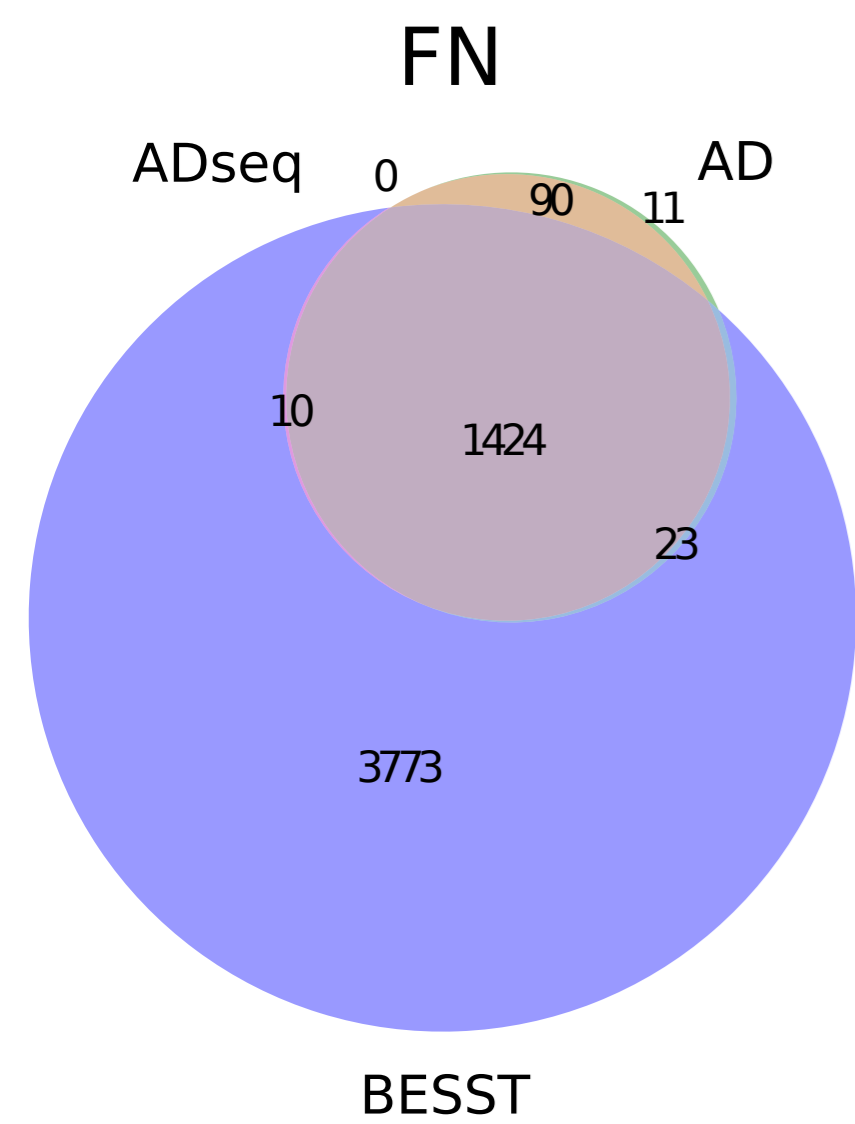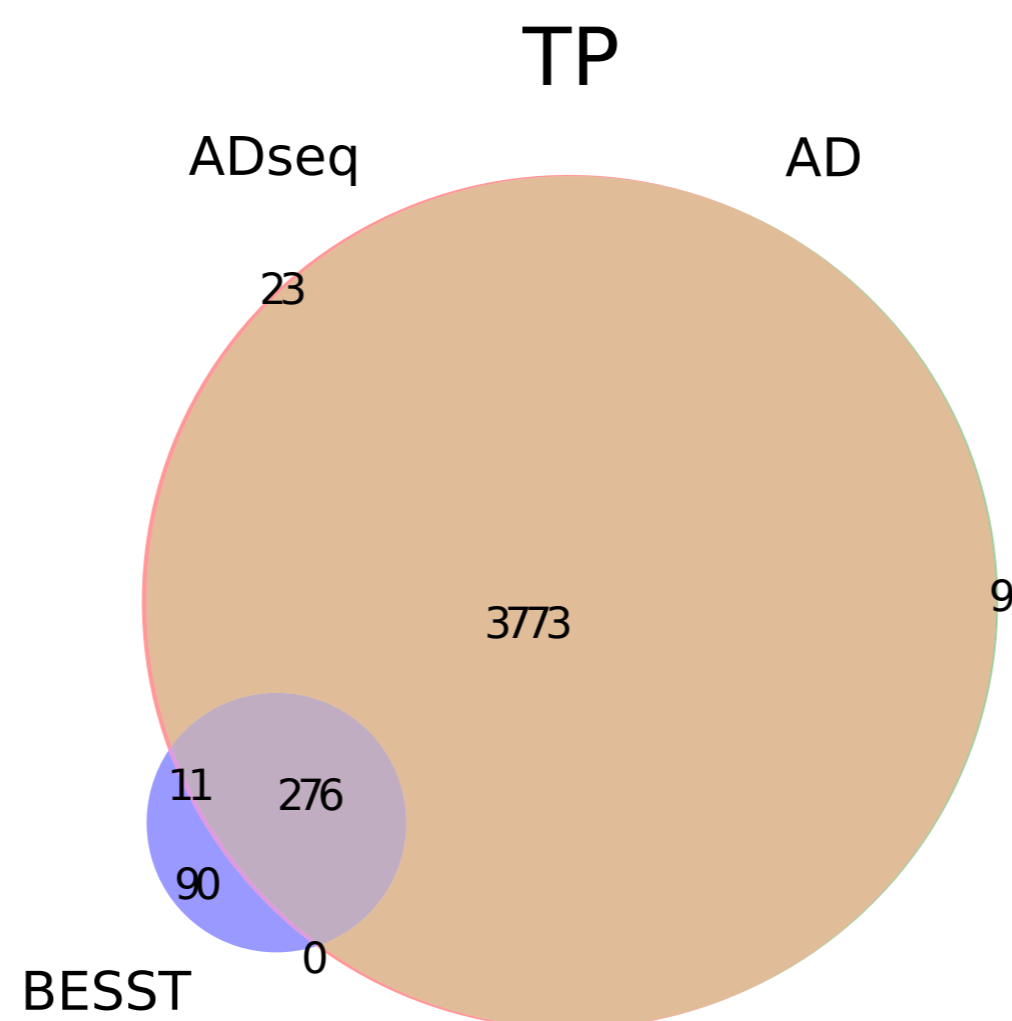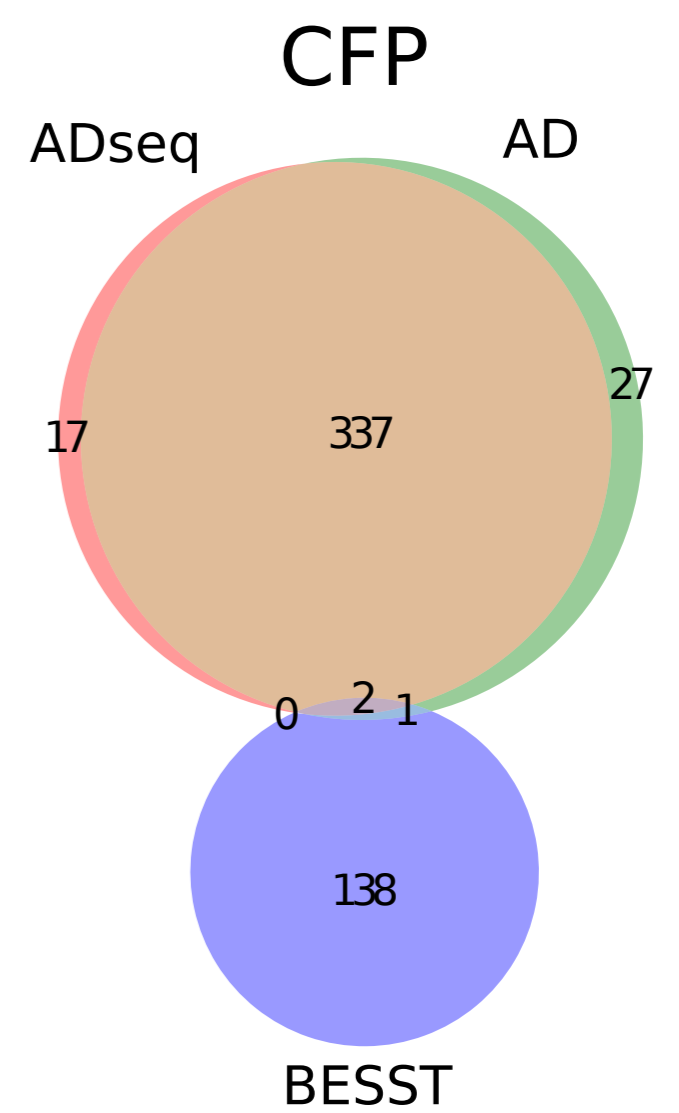

## Anopheles dirus

Supplement: Supplementary file 13 — Figure S12. Similar to Additional file 12: Figure S11 with all reads included. (PDF 20 kb) [file 12864_2018_4466_MOESM13_ESM.pdf]

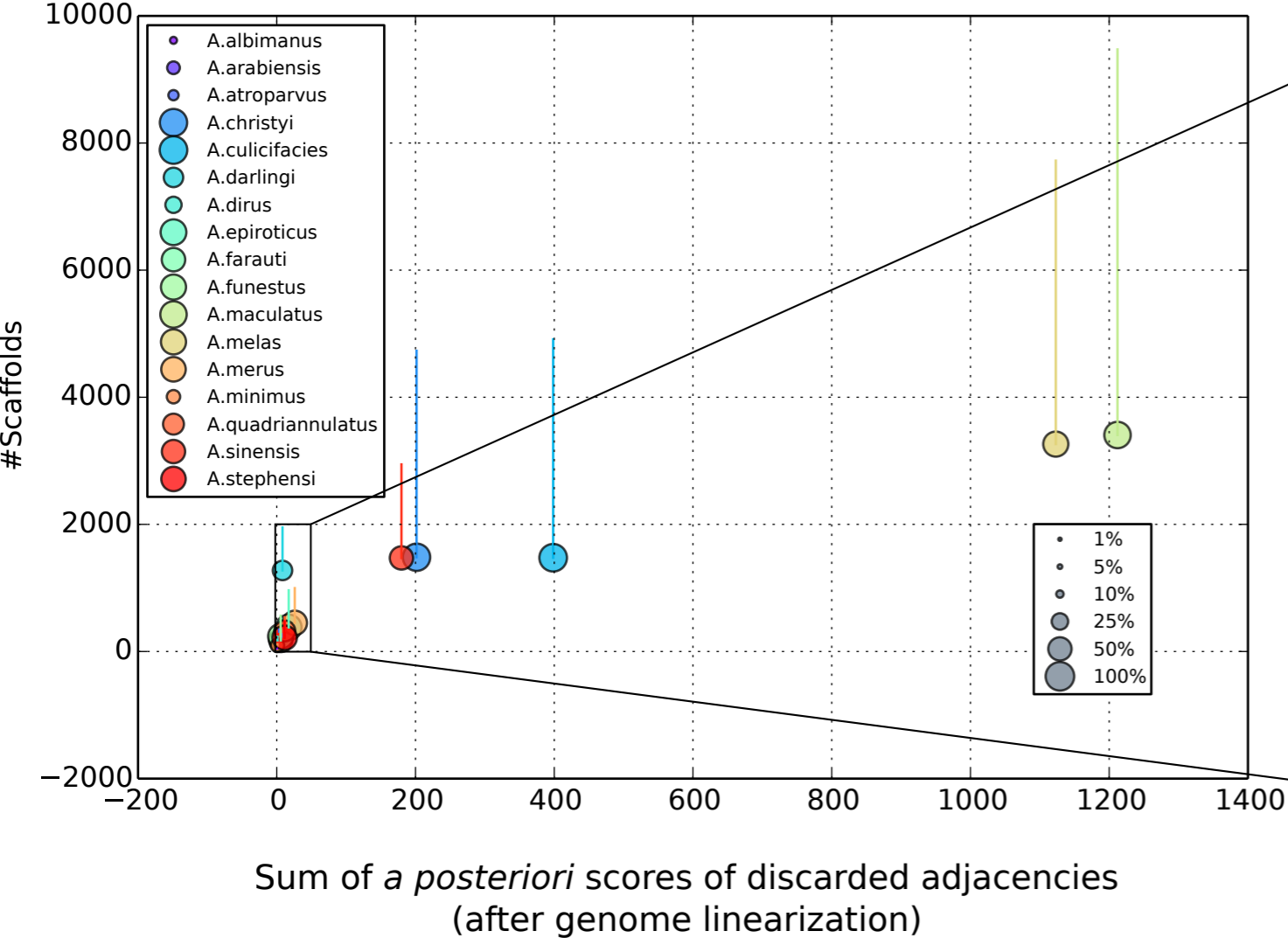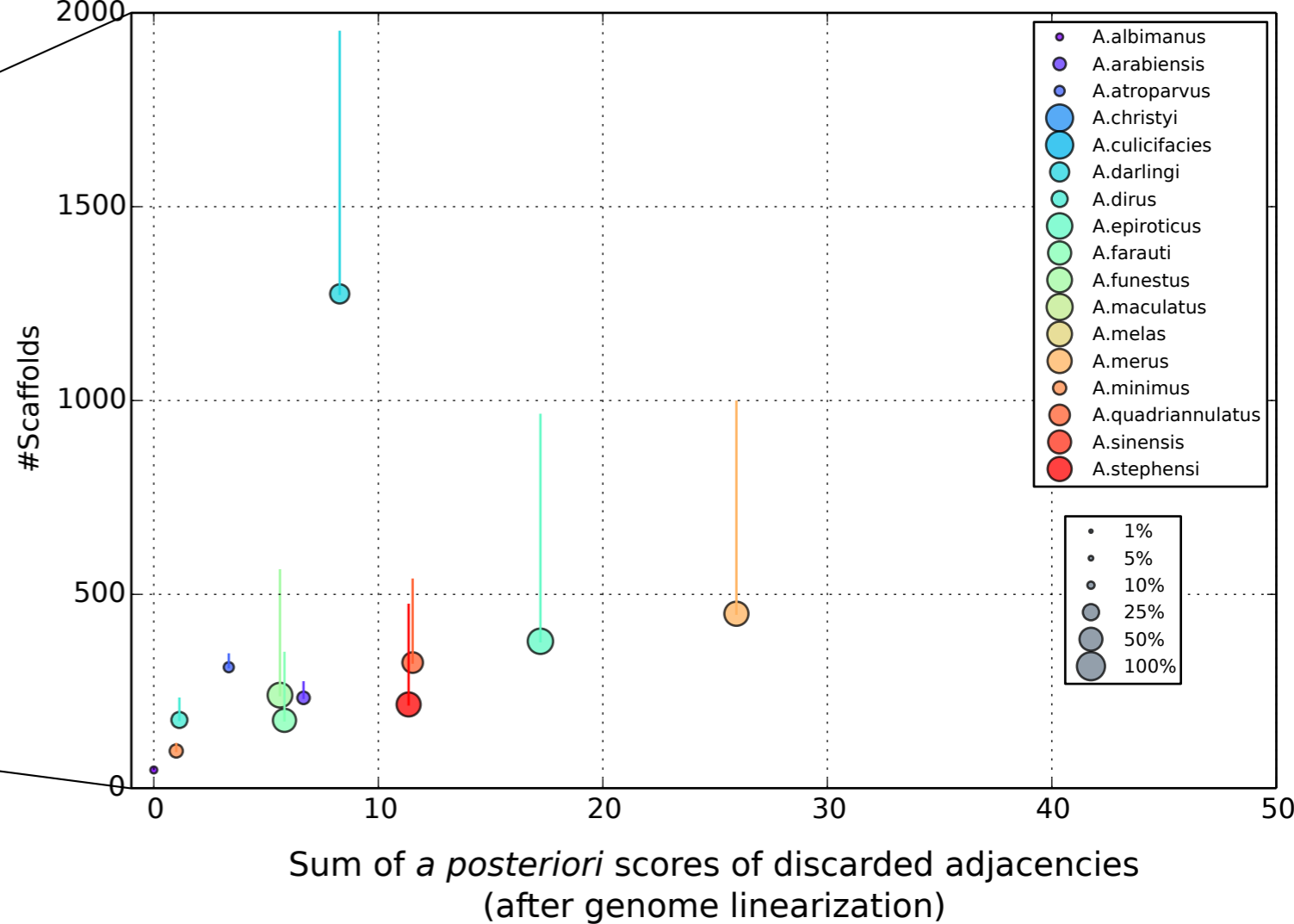

Supplement: Supplementary file 14 — Figure S13. Scatter plot exhibiting scaffolding improvement of the 18 Anopheles genomes by ADseq with X species tree phylogeny. Right plot is a zoom of a small part of the left graph. Each color corresponds to one species. For each species, upper part of vertical line corresponds to number of segments in initial genome assembly and lower part the number of segments after scaffolding improvement by ADseq. Circle diameter is proportional to the % of scaffolding improvement of the genome where scale is displayed in lower right part of the graphs. The X axis represent the sum of a posteriori scores of discarded adjacencies during linearization step representing the degree of syntenic conflicts in adjacencies prediction of ADseq (see paragraph “Conflict” in section “Results”). (PDF 56 kb) [file 12864_2018_4466_MOESM14_ESM.pdf]

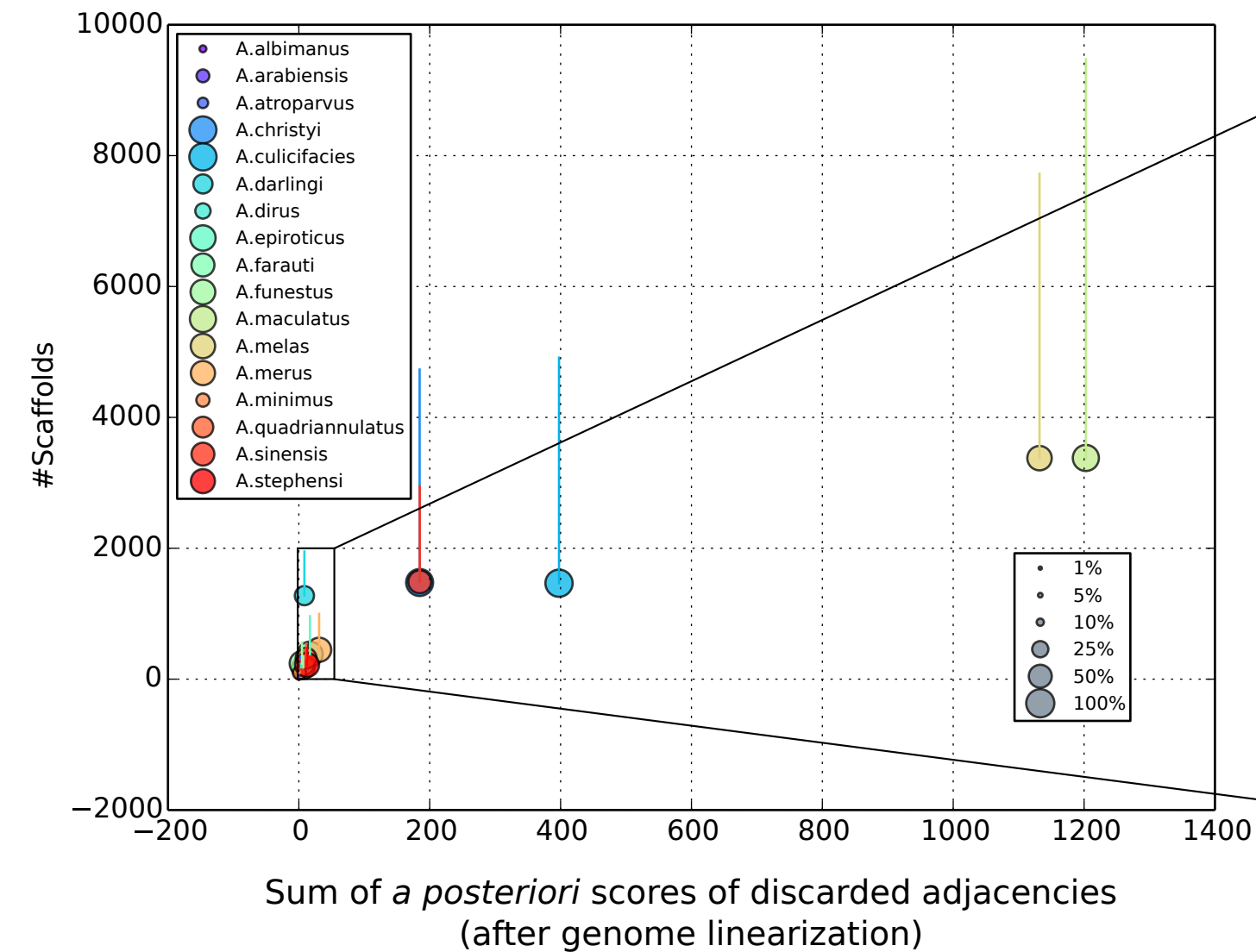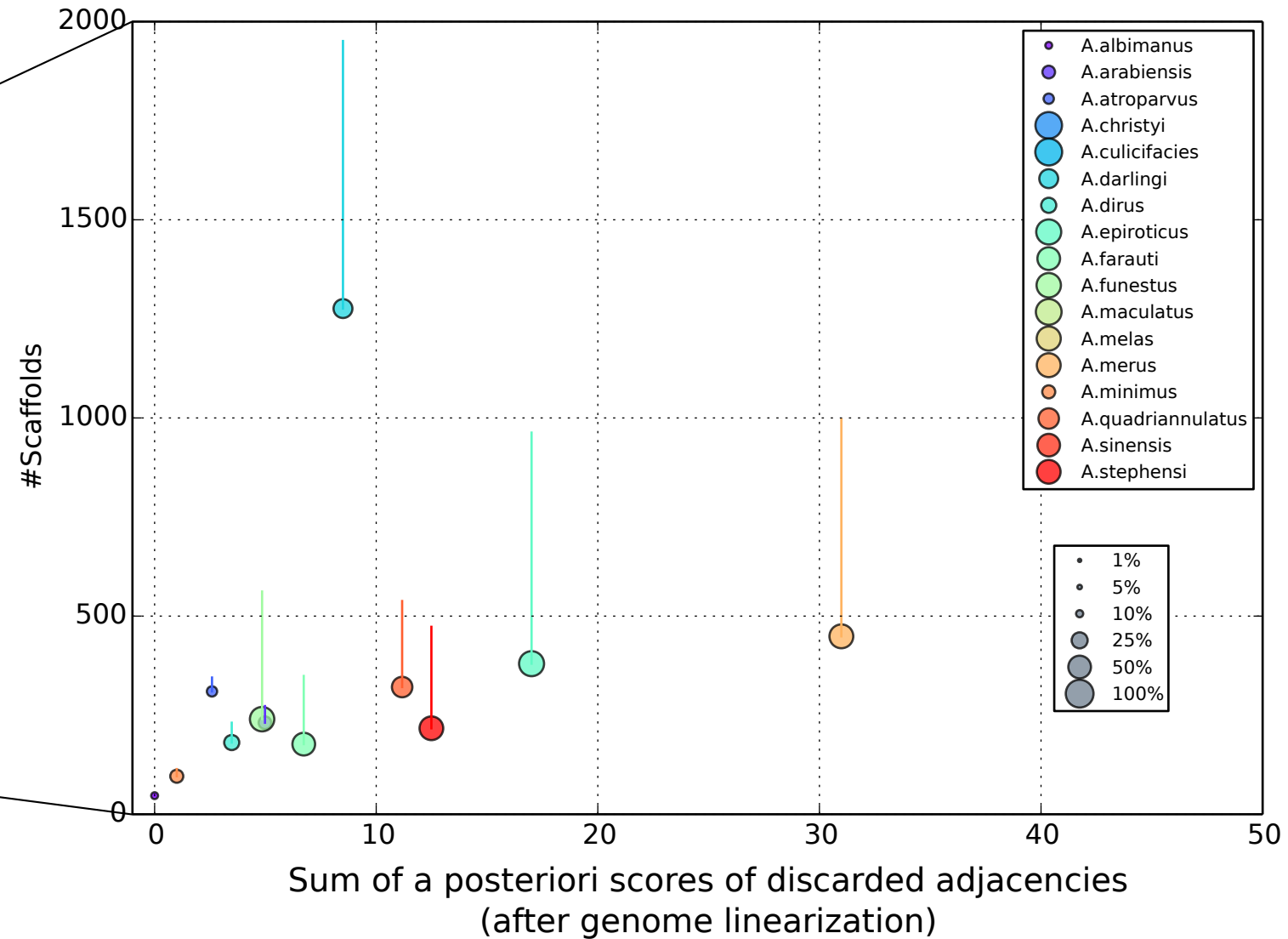

Supplement: Supplementary file 15 — Figure S14. Similar to Additional file 14: Figure S13 with WG species tree phylogeny. (PDF 56 kb) [file 12864_2018_4466_MOESM15_ESM.pdf]

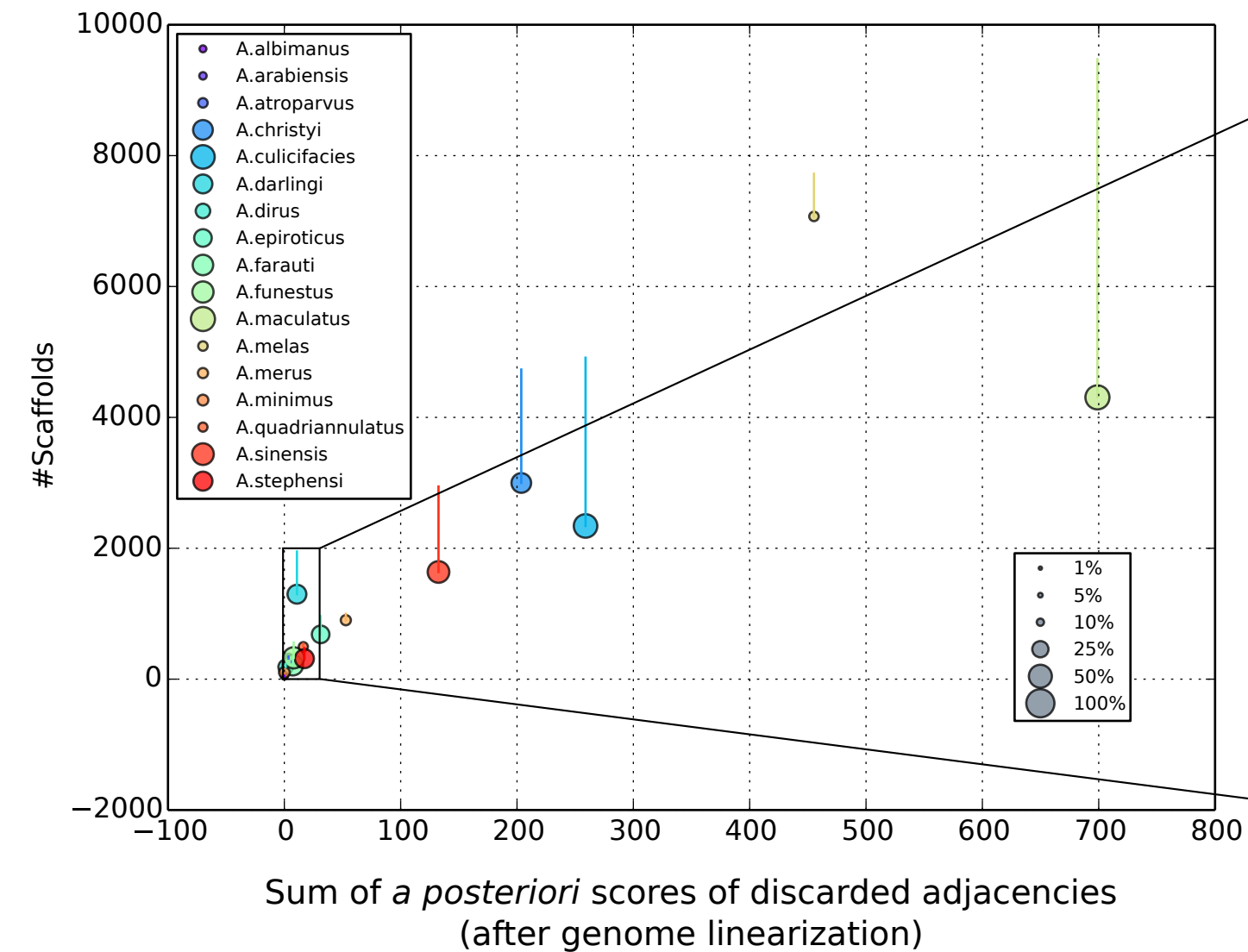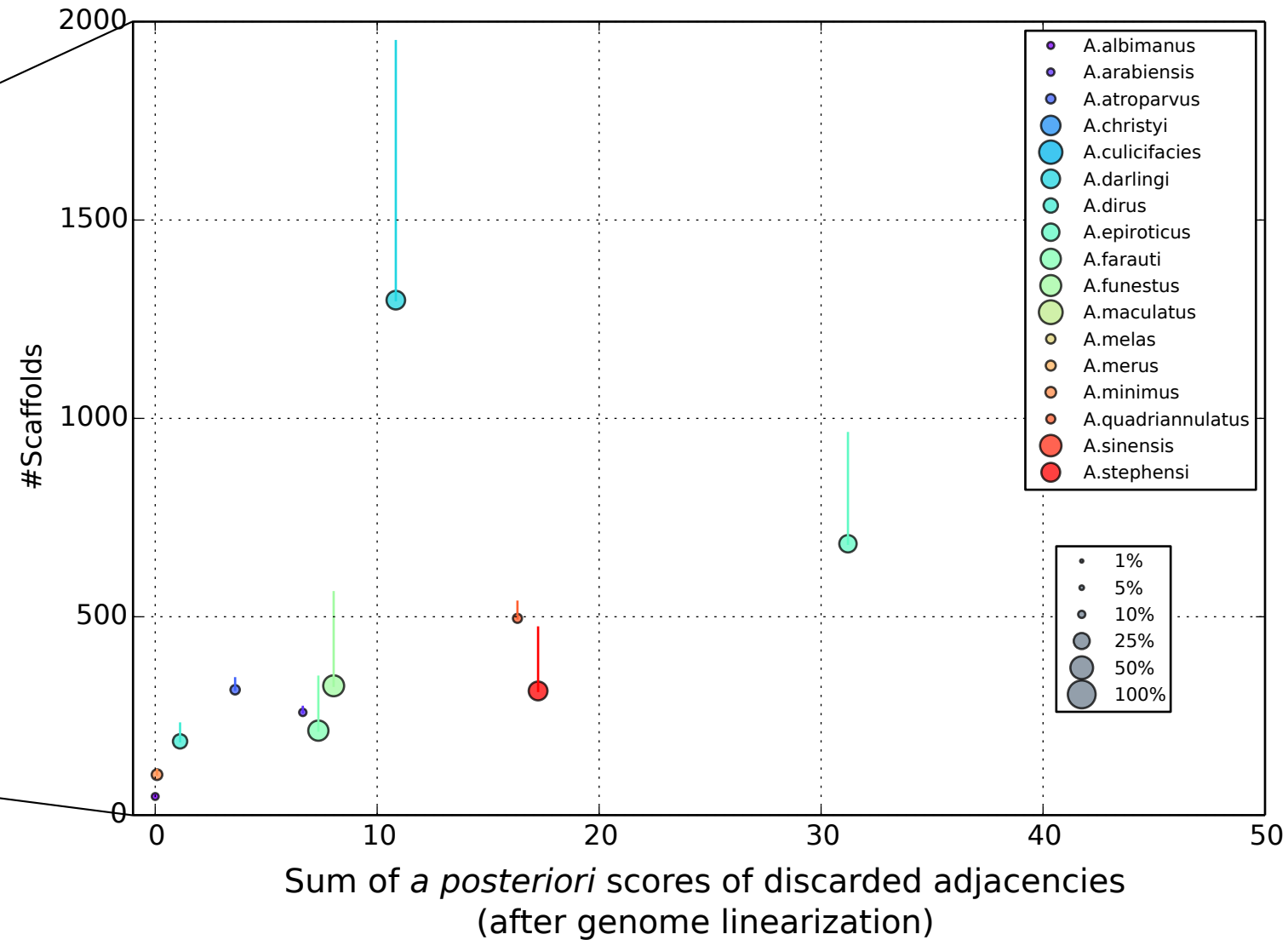

Supplement: Supplementary file 16 — Figure S15. Similar to Additional file 14: Figure S13 with RAW gene trees instead of ProfileNJ gene trees. (PDF 48 kb) [file 12864_2018_4466_MOESM16_ESM.pdf]
