# Supplementary material for: Phylogenetic signal from rearrangements in 18 Anopheles species by joint scaffolding extant and ancestral genomes
Source: BMC Genomics. 2018 May 9;19(Suppl 2):96. doi: 10.1186/s12864-018-4466-7 (PMC5954271; doi:10.1186/s12864-018-4466-7)
Supplement: Supplementary file 3 — Figure S2. The pipeline is split into two parts: The first part (Blue one) processes genome content data to obtain extant genome adjacencies. Step 1 detects genes that are included in other genes. In Step 2, gene families containing these genes are filtered out to avoid ambiguity in defining observed extant gene adjacencies. In Step 3, gene trees are inferred from the gene sequences, one gene tree per gene family (see Additional file 5: Figure S4 for more information on gene trees inference pipeline). Finally, in Step 4 genes contained in gene families for which gene trees have not been inferred are discarded from the analysis (41 gene families containing representing 1,039 genes). The second part of the pipeline (Green one) processes sequencing data to obtain scaffolding adjacencies that will be used to improve extant genome assembly with the ADseq algorithm. Step A trims reads with Trimmomatic to remove low qualities reads and remaining adapters. In Step B, trimmed reads are mapped onto their respective genome with Bowtie2 considering all multiple mappings. In Step C, pairs of contigs for which paired-end reads suggest a possible contiguity along their chromosome are linked with the scaffolding software BESST, and the resulting potential scaffolding adjacencies are scored according to the BESST model. Then, in Step 5 scaffolding gene adjacencies are determined from contigs adjacencies obtained from sequencing data processing part with genes present in gene trees. This results in scaffolds with observed scored scaffolding adjacencies that are used as input of DeCoSTAR (Step 6). See Additional file 18: Table S2 for a description per species on dataset used for DeCoSTAR. Pipeline to produce input data for ADseq on 18 Anopheles dataset. The pipeline takes as input a species tree for the 18 Anopheles species, the whole set of gene families and gene trees for these species and genomic data (contigs, scaffolds and chromosomes). The goal of the pipeline is to produce inpu [file 12864_2018_4466_MOESM3_ESM.pdf]

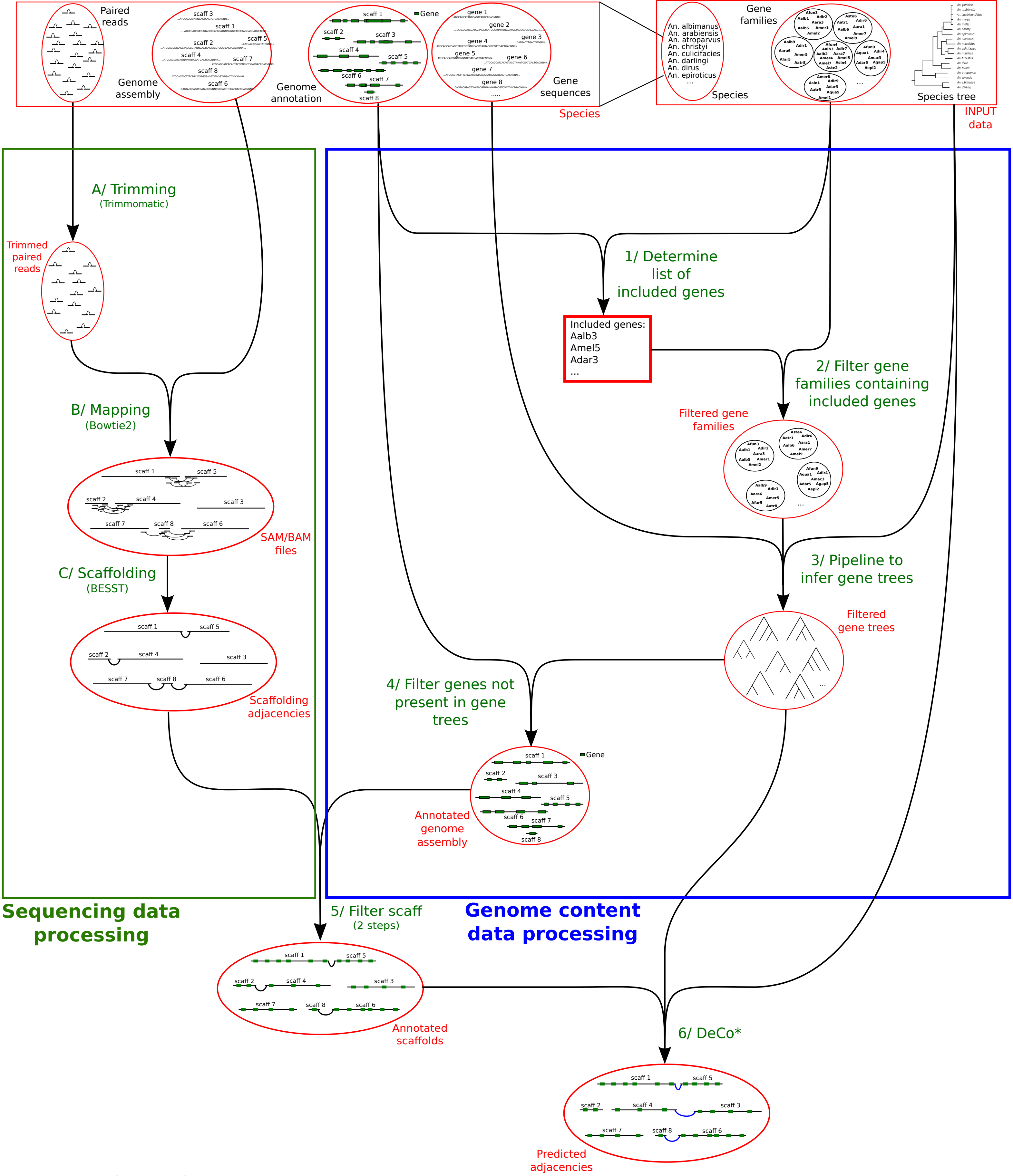

The first part (Blue one) processes genome content data to obtain extant genome adjacencies. **Step 1** detects genes that are included in other genes. In **Step 2**, gene families containing these genes are filtered out to avoid ambiguity in defining observed extant gene adjacencies. In **Step 3**, gene trees are inferred from the gene sequences, one gene tree per gene family (see Additional file 5: Figure S4 for more information on gene trees inference pipeline). Finally, in **Step 4** genes contained in gene families for which gene trees have not been inferred are discarded from the analysis (41 gene families containing representing 1,039 genes). The second part of the pipeline (Green one) processes sequencing data to obtain scaffolding adjacencies that will be used to improve extant genome assembly with the ADSEQ algorithm. **Step A** trims reads with TRIMMOMATIC to remove low qualities reads and remaining adapters. In **Step B**, trimmed reads are mapped onto their respective genome with BOWTIE2 considering all multiple mappings. In **Step C**, pairs of contigs for which paired-end reads suggest a possible contiguity along their chromosome are linked with the scaffolding software BESST, and the resulting potential scaffolding adjacencies are scored according to the BESST model. Then, in **Step 5** scaffolding gene adjacencies are determined from contigs adjacencies obtained from sequencing data processing part with genes present in gene trees. This results in scaffolds with observed scored scaffolding adjacencies that are used as input of DECOSTAR (**Step 6**). See Additional file 18: Table S2 for a description per species on dataset used for DECOSTAR.
