# Supplementary material for: Phylogenetic signal from rearrangements in 18 Anopheles species by joint scaffolding extant and ancestral genomes
Source: BMC Genomics. 2018 May 9;19(Suppl 2):96. doi: 10.1186/s12864-018-4466-7 (PMC5954271; doi:10.1186/s12864-018-4466-7)
Supplement: Supplementary file 5 — Figure S4. Pipeline to improve gene trees inference from homologous gene family. CDS sequences of genes in gene trees have been obtained from VectorBase database and homologous gene families deduced from the 14,981 gene trees resulting of step 2 of Additional file 5: Figure S4. Step A consists to multiple align homologous genes with Muscle with parameter “-maxiters 2” if a gene sequence have a size upper than 32,000 bp. In Step B, Gblocks was applied on alignments to select high confidence alignment sites. At this step, 41 gene families have been discarded due to sequences that were not present in a selected blocks. For Step C, RAxML have been used to infer maximum likelihood gene trees with the GTR-GAMMA model and 100 bootstrap iterations. Finally in Step D, the maximum likelihood gene trees are processed with ProfileNJ to potentially changing branches with bootstrap support lower than 100% in a DL reconciliation model (min(Duplication,Loss)) with the species tree [73]. (PDF 35 kb) [file 12864_2018_4466_MOESM5_ESM.pdf]

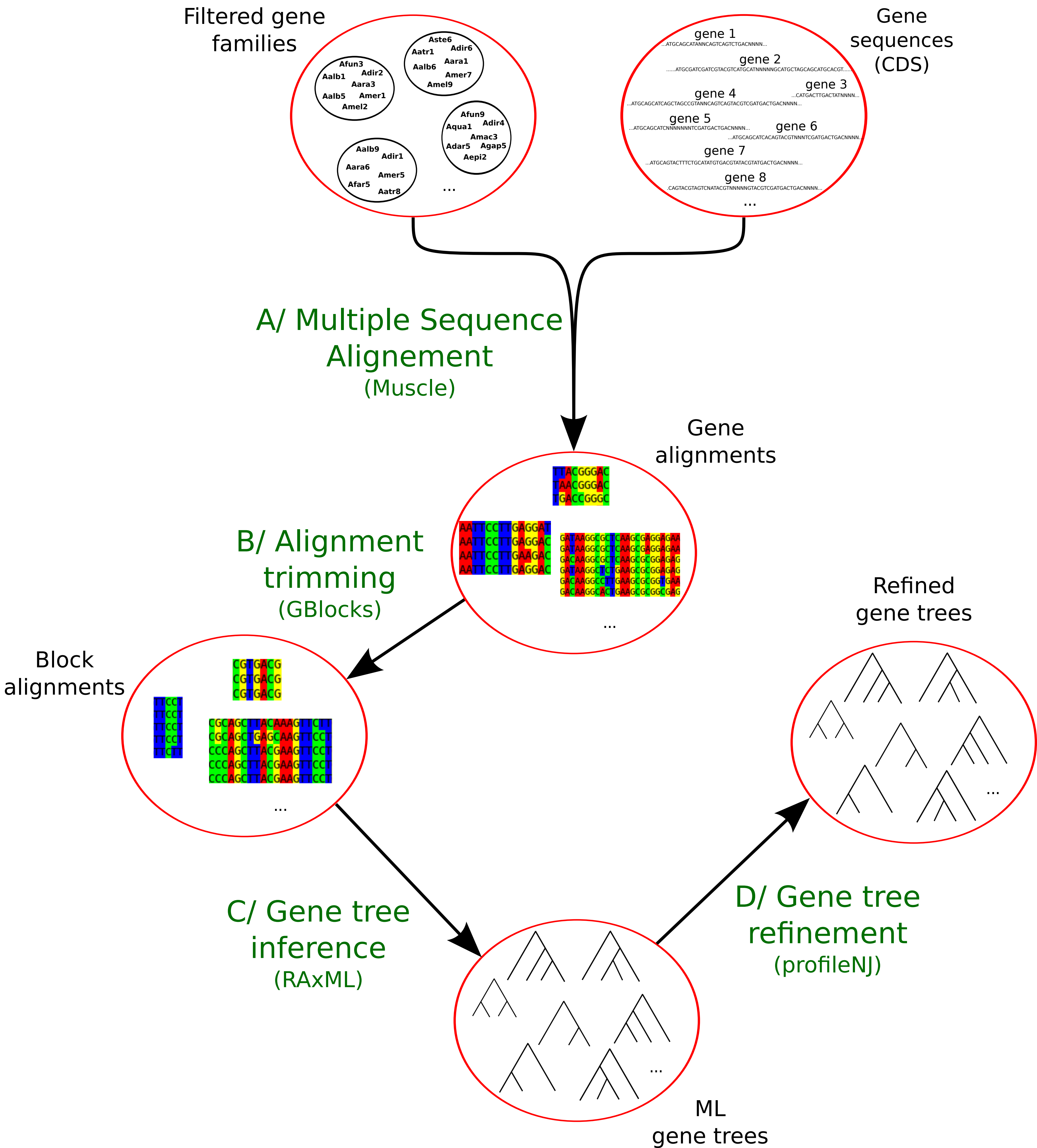
Filtered gene families
Gene sequences (CDS)
A/ Multiple Sequence Alignement
(Muscle)
Gene alignments
B/ Alignment trimming
(GBlocks)
Block alignments
C/ Gene tree inference
(RAxML)
ML gene trees
D/ Gene tree refinement
(profileNJ)
Refined gene trees
