# Supplementary material for: Phylogenetic signal from rearrangements in 18 Anopheles species by joint scaffolding extant and ancestral genomes
Source: BMC Genomics. 2018 May 9;19(Suppl 2):96. doi: 10.1186/s12864-018-4466-7 (PMC5954271; doi:10.1186/s12864-018-4466-7)
Supplement: Supplementary file 17 — Table S1. Summary of genome assemblies and sequencing data information. 16 on the 18 Anopheles species have been sequenced in [4] and data are available on the SRA database of the NCBI (see column 4 and 6 for BioProject and SRA ID). FASTQ files of paired sequencing data have been obtained with SRA-toolkit. After mapping of paired reads on reference genome assemblies (column 2), median insert size of libraries have been determined with package “CollectInsertSizeMetrics” of Picard Tools (v1.61) (column 7). Column “Library name” give information on the sequencing strategies employed in [4]. Where ’fragment’ library corresponds to a Paired-End library with an expected insert size of 180bp and FR orientation (→←). ’jump’ library corresponds to a Mate-Pair library with an insert size of 1.5kbp and RF orientation (←→). And ’fosill’ corresponds to a library generated from a pool of hundred mosquitoes to improve the scaffolding with an expected insert size around 38kbp and FR orientation (→←). Column 3 gives the ID of gene set used in this study. (PDF 38 kb) [file 12864_2018_4466_MOESM17_ESM.pdf]

Species name	Assembly name	Gene set	BioProject	Library name	SRA ID	Median insert size (bp)
An. albimanus	AalbS1	AalbS1.1	PRJNA67235	'fosill'	SRX200219	35,557
				'jump'	SRX111456	2,408
				'fragment'	SRX084279	194
An. arabiensis	AaraD1	AaraD1.1	PRJNA67207	'fosill'	SRX200218	36,444
				'jump'	SRX111457	2,051
				'fragment'	SRX084275	195
An. atroparvus	AatrE1	AatrE1.1	PRJNA67233	'fosill'	SRX209222	36,897
				'jump'	SRX209384	2,408
					SRX209606	2,382
				'fragment'	SRX209390	191
					SRX209612	191
An. christyi	AchrA1	AchrA1.1	PRJNA67213	'jump'	SRX110286	1,242
					SRX119723	1,229
				'fragment'	SRX084278	195
An. culicifacies	AculA1	AculA1.1	PRJNA163119	'jump'	SRX175835	546
					SRX334058	1,156
				'fragment'	SRX158118	181
					SRX182921	182
					SRX189771	183
					SRX272317	196
An. darlingi	AdarC2	AdarC2.2	NA	NA	NA	NA
An. dirus	AdirW1	AdirW1.1	PRJNA196855	'fosill'	SRX209221	36,451
				'jump'	SRX209379	2,378
					SRX209603	2,354
				'fragment'	SRX209381	191
					SRX209604	191
An. epiroticus	AepiE1	AepiE1.1	PRJNA191562	'jump'	SRX209380	854
					SRX209614	822
				'fragment'	SRX209391	191
					SRX209605	191
An. farauti	AfarF1	AfarF1.1	PRJNA67229	'fosill'	SRX349764	404
			PRJNA214011	'fosill'	SRX357088	405
					SRX357089	405
				'jump'	SRX111458	1,976
				'fragment'	SRX084280	175
An. funestus	AfunF1	AfunF1.1	PRJNA67223	'fosill"	SRX209224	36,450
				'jump'	SRX209389	2,010
					SRX209610	1,979
				'fragment'	SRX209387	192
					SRX209628	192
An. gambiae	AgamP3	AgamP3.8	NA	NA	NA	NA
An. maculatus	AmacM1	AmacM1.1	PRJNA67215	'jump'	SRX209385	709
					SRX209609	682
				'fragment'	SRX209386	191
					SRX209629	191
An. melas	AmelC1	AmelC1.1	PRJNA163117	'jump'	SRX175836	651
				'fragment'	SRX158119	176
					SRX184877	177
					SRX189770	179
An. merus	AmerM1	AmerM1.1	PRJNA67215	'fosill'	SRX349762	37,890
					SRX357090	37,880
					SRX357091	37,882
				'jump'	SRX110236	1,383
				'fragment'	SRX084276	195
An. minimus	AminM1	AminM1.1	PRJNA67225	'fosill'	SRX209223	36,838
				'jump'	SRX209388	2,296
					SRX209608	2,272
				'fragment'	SRX209383	192
					SRX209627	192
An. quadriannulatus	AquaS1	AquaS1.1	NA	NA	NA	NA
An. sinensis	AsinS1	AsinS1.1	PRJNA214011	'fosill'	SRX349763	38,486
					SRX357092	37,880
					SRX357093	37,882
				'jump'	SRX334057	2,373
				'fragment'	SRX334056	187
An. stephensi	AsteS1	AsteS1.1	NA	NA	NA	NA
