# Supplementary material for: Phylogenetic signal from rearrangements in 18 Anopheles species by joint scaffolding extant and ancestral genomes
Source: BMC Genomics. 2018 May 9;19(Suppl 2):96. doi: 10.1186/s12864-018-4466-7 (PMC5954271; doi:10.1186/s12864-018-4466-7)
Supplement: Supplementary file 18 — Table S2. Assembly statistics on the 18 Anopheles genomes. Statistics before before processing are displayed in columns 2-7 and after the pipeline to produce input data for the DeCoSTAR algorithm in columns 8-11 (see Additional file 3: Figure S2 for illustration of the data preprocessing step). For initial dataset assembly statistics, columns 2 and 3 present contigs number and N50 statistic in bp for all contigs in genome assemblies. In columns 4-7, only contigs with at least one gene are considered. Column 4 corresponds to contigs number with gene in reference assemblies. Columns 5 & 6 represent N50 statistics respectively in bp and in gene number. Column 7 represent the number of gene in reference genome assemblies. For genome assemblies used as input of DeCoSTAR, all contigs contains at least one gene. Column 8 gives the number of contigs after step 4 of Additional file 3: Figure S2. Columns 9 & 10 represent N50 statistics respectively in bp and in gene number. And column 11 represents the number of gene in genomes taken as input of DeCoSTAR. The input dataset of DeCoSTAR is composed of 14,940 gene trees (see Additional file 4: Figure S3 and Additional file 5: Figure S4 for more information on gene trees) and 68,876 gene adjacencies with sequence support (scaffolding gene adjacencies) (see Additional file 6: Figure S5 and Additional file 7: Figure S6 for more information on scaffolding adjacencies). (PDF 33 kb) [file 12864_2018_4466_MOESM18_ESM.pdf]

species name	initial dataset						post data preprocessing			
	all contigs		contigs with gene				contigs with gene			
	#CTG	N50 (bp)	#CTG	N50		#gene	#CTG	N50		#gene
				bp	#gene			bp	#gene	
An. albimanus	204	18,068,499	57	18,068,499	1,212	11,911	49	18,068,499	916	9,056
An. arabiensis	1,214	5,604,218	340	5,830,121	348	13,162	273	5,830,121	321	10,298
An. atroparvus	1,371	9,206,694	476	9,206,694	655	13,776	345	9,206,694	512	10,400
An. christyi	30,369	9,057	5,173	17,016	3	10,738	4,731	17,384	2	8,792
An. culicifacies	16,162	22,320	5,715	32,742	4	14,335	4,912	34,064	3	11,213
An. darlingi	2,160	115,168	2,161	115,168	10	10,457	1,951	118,843	9	8,617
An. dirus	1,266	6,906,475	302	7,656,907	543	12,781	231	7,656,907	406	9,883
An. epiroticus	2,673	366,526	1,052	417,110	29	12,078	963	425,117	24	9,855
An. farauti	550	1,196,527	376	1,235,781	84	13,217	349	1,235,781	64	10,239
An. funestus	1,392	671,960	619	702,105	46	13,344	562	703,988	36	10,077
An. gambiae	7	49,364,325	6	49,364,325	2,867	12,810	6	49,364,325	2,339	10,324
An. maculatus	47,797	3,841	12,776	4,751	1	14,835	9,473	5,042	1	10,552
An. melas	20,281	18,041	8,855	21,239	2	16,149	7,723	21,730	2	12,567
An. merus	2,753	342,196	1,078	391,600	886	13,887	997	400,239	23	10,736
An. minimus	678	10,313,149	142	10,313 149	886	12,560	114	10,313,149	682	9,792
An. quad.	2,823	1,641,272	647	1,794,736	95	13,349	538	1,846,441	74	10,289
An. sinensis	11,270	80,738	3,536	103,937	9	14,791	2,944	109,624	7	10,962
An. stephensi	1,110	837,295	502	851,727	57	13,113	473	851,727	44	10,028
All species	144,080	760,870	43,813	1,159,817	45	237,293	36,634	1,272,063	37	183,680
