# Supplementary material for: Phylogenetic signal from rearrangements in 18 Anopheles species by joint scaffolding extant and ancestral genomes
Source: BMC Genomics. 2018 May 9;19(Suppl 2):96. doi: 10.1186/s12864-018-4466-7 (PMC5954271; doi:10.1186/s12864-018-4466-7)
Supplement: Supplementary file 19 — Table S3. Assembly statistics at various stages of the gene annotation step of validation protocol of ADseq (step 4 of Additional file 8: Figure S7). For each species and annotation step, table gives different assembly statistics (column2): the number of contigs in the assembly, the size of the assembly in bp and in gene number, the N50 statistics of the assembly in bp and in gene number (if available) and gene trees number corresponding to gene present in the assembly (for the two last columns). Column 3 (Initial assembly) corresponds to the assembly statistics of reference genomes. Columns 4-8 of upper and lower table corresponds to Minia assembly statistics at different filtering step respectively with 50% reads sampling and without reads sampling. Column “initial” corresponds to assembly in output of Minia algorithm assembly. Minia contigs are then mapped on reference genome to annotate gene of reference assembly on Minia contigs. Assembly statistics after filter1 corresponds to Minia contigs that have been mapped on reference assembly with an identity and a coverage >=90%. Filter2 consists to keep only contig with an unique optimal alignment (to avoid uncertainty in gene annotation). Column 7 corresponds to Minia assembly statistics after merging of Minia contigs overlapping a same gene (simulating RNA-seq scaffolding). Then last column corresponds to statistics after filter3 that consists to discard gene families of genes that have not been mapped on Minia contigs. (PDF 51 kb) [file 12864_2018_4466_MOESM19_ESM.pdf]

Species name	Assembly stats	Initial assembly	MINIA assembly **(50% reads)**				
			initial	after filter1	after filter2	after merging	after filter3
An. albimanus	kmer size	NA	75				
	#CTG	204	93,906	86,698	86,307	5,555	5,547
	Size (bp)	170,508,315	170,159,531	167,477,606	167,368,303	69,154,374	69,071,024
	#gene)	NA				9,030	9,018
	N50 (bp)	18,068,499	4,833	4,908	4,911	17,015	17,013
	N50 (#gene)	NA				2	2
	#gene trees	NA				14,940	14,915
An. arabiensis	kmer size	NA	59				
	#CTG	1,214	302,287	243,251	238,596	7,974	7,968
	Size (bp)	246,567,867	231,833,497	219,419,350	218,591,584	64,718,036	64,675,874
	#gene	NA				10,274	10,268
	N50 (bp)	5,604,218	2,193	2,384	2397	11,132	11,123
	N50 (#gene)	NA				1	1
	#gene trees	NA				14,940	14,918
An. dirus	kmer size	NA	63				
	#CTG	1,266	220,053	164,611	160,972	5,892	5,888
	Size (bp)	216,307,690	217,905,932	202,370,679	201,700,338	89,145,793	89,115,176
	#gene	NA				9,789	9,781
	N50 (bp)	18,068,499	7,281	8,455	8,521	25,298	25,230
	N50 (#gene)	NA				2	2
	#gene trees	NA				14,940	14,846

Species name	Assembly stats	Initial assembly	MINIA assembly **(100% reads)**				
			initial	after filter1	after filter2	after merging	after filter3
An. albimanus	kmer size	NA	83				
	#CTG	204	71,361	64,512	64,179	4,852	4,845
	Size (bp)	170,508,315	169,477,186	166,370,462	166,259,421	77,887,807	77,807,527
	#gene	NA				9,012	9,000
	N50 (bp)	18,068,499	7,564	7,688	7,705	21,801	21,801
	N50 (#gene)	NA				2	2
	#gene trees	NA				14,940	14,898
An. arabiensis	kmer size	NA	72				
	#CTG	1,214	229,218	184,605	181,423	7,133	7,127
	Size (bp)	246,567,867	232,286,601	219,658,117	218,990,676	80,623,434	80,568,561
	#gene	NA				10,253	10,246
	N50 (bp)	5,604,218	4,322	4,838	4,864	17,147	17,147
	N50 (#gene)	NA				1	1
	#gene trees	NA				14,940	14,896
An. dirus	kmer size	NA	75				
	#CTG	1,266	210,188	155,771	153,031	5,836	5,829
	Size (bp)	216,307,690	220,937,663	202,341,639	201,719,647	91,254,277	91,196,154
	#gene	NA				9,759	9,748
	N50 (bp)	18,068,499	7,666	9,044	9,115	27,134	27,141
	N50 (#gene)	NA				2	2
	#gene trees	NA				14,940	14,816
