# Supplementary material for: Phylogenetic signal from rearrangements in 18 Anopheles species by joint scaffolding extant and ancestral genomes
Source: BMC Genomics. 2018 May 9;19(Suppl 2):96. doi: 10.1186/s12864-018-4466-7 (PMC5954271; doi:10.1186/s12864-018-4466-7)
Supplement: Supplementary file 20 — Table S4. Scaffolding statistics on the 18 Anopheles genomes before and after ADseq (with the X (upper table) and WG (lower table) species phylogenies). The columns 2-5 correspond to assemblies statistics before running the ADseq algorithm. Column 2 corresponds to the number of contigs in reference assemblies. The N50 statistic corresponding to the contig size where 50% of the total assembly length is comprised in contigs with size superior or equal to this value. This metric is computed with size considerd both in bp (in column 3) and in gene number (in column 4). Column 5 gives the number of genes in genome assemblies give as input to ADseq. Columns 6-9 and 10-13 represent scaffolding statistics of ADseq respectively for X chromosome species tree topology and Whole-Genome topology. Columns 6 & 10 represent scaffolds number after ADseq. Columns 7 & 11, and 8 & 12 represent N50 statistics respectively for size in bp and size in gene number. Columns 9 & 13 represent new adjacencies inferred by ADseq (#scaff adj) represent the number of new adjacencies that are scaffolding adjacencies (i.e. adjacencies with sequence signal proposed by BESST and inferred by ADseq). (PDF 56 kb) [file 12864_2018_4466_MOESM20_ESM.pdf]

Species name	Genome assemblies before ADseq				Genome scaffolds after ADseq **(X topology)**			
	contigs with gene				scaffolds with gene			
	#CTG	N50		#gene	#scaffolds	N50		#new adj (#scaff adj)
		bp	#gene			bp	#gene	
An. albimanus	49	18,068,499	916	9,056	47	18,068,499	916	2 (2)
An. arabiensis	273	5,830 121	321	10,298	216	9,217,108	410	57 (13)
An. atroparvus	345	9,206,694	512	10,400	306	10,083,987	647	39 (12)
An. christyi	4,731	17,384	2	8,792	1,396	95,212	12	3,335 (204)
An. culicifacies	4,912	34,064	3	11,213	1,339	202,550	19	3,574 (1,366)
An. darlingi	1,951	118,843	9	8,617	1,264	197,002	13	687 (NA)
An. dirus	231	7,656,907	406	9,883	176	17,377,229	778	55 (10)
An. epiroticus	963	425,117	24	9,855	369	1,611,558	78	594 (7)
An. farauti	349	1,235,781	64	10,239	169	2,391,621	146	180 (64)
An. funestus	562	703,988	36	10,077	231	2,772,343	127	331 (112)
An. gambiae	6	49,364,325	2,339	10,324	6	49,364,325	2,339	0 (NA)
An. maculatus	9,473	5,042	1	10,552	3,025	30,779	7	6,448 (295)
An. melas	7,723	21,730	2	12,567	2,685	92,676	9	5,038 (165)
An. merus	997	400,239	23	10,736	419	1,183,618	65	578 (391)
An. minimus	114	10,313,149	682	9,792	96	17,164,539	801	18 (7)
An. quadriannulatus	538	1,846,441	74	10,289	294	5,492,301	206	244 (0)
An. sinensis	2,944	109,624	7	10,962	1,325	293,848	20	1,619 (478)
An. stephensi	473	851,727	44	10,028	204	2,772,062	131	269 (0)
All species	36,634	1,272,063	37	183,680	13,567	3,261,557	94	23,068 (3,126)

Species name	Genome assemblies before ADseq				Genome scaffolds after ADseq **(Whole Genome topology)**			
	contigs with gene				scaffolds with gene			
	#CTG	N50		#gene	#scaffolds	N50		#new adj (#scaff adj)
		bp	#gene			bp	#gene	
An. albimanus	49	18,068,499	916	9,056	47	18,068,499	916	2 (2)
An. arabiensis	273	5,830 121	321	10,298	214	9,972,103	464	59 (14)
An. atroparvus	345	9,206,694	512	10,400	307	10,083,987	647	38 (12)
An. christyi	4,731	17,384	2	8,792	1,408	93,948	12	3,323 (207)
An. culicifacies	4,912	34,064	3	11,213	1,338	208,611	19	3,575 (1,363)
An. darlingi	1,951	118,843	9	8,617	1,265	197,190	14	686 (NA)
An. dirus	231	7,656,907	406	9,883	176	17,377,229	778	55 (10)
An. epiroticus	963	425,117	24	9,855	368	1,662,136	78	595 (7)
An. farauti	349	1,235,781	64	10,239	170	2,391,621	146	179 (63)
An. funestus	562	703,988	36	10,077	232	2,673,183	127	330 (112)
An. gambiae	6	49,364,325	2,339	10,324	6	49,364,325	2,339	0 (NA)
An. maculatus	9,473	5,042	1	10,552	3,023	31,226	7	6,450 (297)
An. melas	7,723	21,730	2	12,567	2,643	94,004	9	5,080 (162)
An. merus	997	400,239	23	10,736	406	1,260,898	65	591 (399)
An. minimus	114	10,313,149	682	9,792	96	17,164,539	801	18 (7)
An. quadriannulatus	538	1,846,441	74	10,289	297	4,868,888	206	241 (0)
An. sinensis	2,944	109,624	7	10,962	1,325	297,247	19	1,619 (475)
An. stephensi	473	851,727	44	10,028	204	2,792,811	131	269 (0)
All species	36,634	1,272,063	37	183,680	13,525	3,261,557	94	23,110 (3,130)
